# Superparamagnetic Nanoparticles with Phosphorescent Complexes as Hybrid Contrast Agents: Integration of MRI and PLIM

**DOI:** 10.1002/smsc.202300145

**Published:** 2024-01-12

**Authors:** Maria Belen Rivas Aiello, Thomas M. Kirse, Gabriel C. Lavorato, Bastian Maus, Iván Maisuls, Shivadharshini Kuberasivakumaran, Stefan Ostendorp, Alexander Hepp, Michael Holtkamp, Elin L. Winkler, Uwe Karst, Gerhard Wilde, Cornelius Faber, Carolina Vericat, Cristian A. Strassert

**Affiliations:** ^1^ Instituto de Investigaciones Fisicoquímicas Teóricas y Aplicadas (INIFTA) Universidad Nacional de La Plata – CONICET 1900 La Plata Buenos Aires Argentina; ^2^ CeNTech, CiMIC, SoN Universität Münster Heisenbergstraße 11 D‐48149 Münster Germany; ^3^ Institut für Anorganische und Analytische Chemie Universität Münster Corrensstraße 28/30 D‐48149 Münster Germany; ^4^ Klinik für Radiologie Translational Research Imaging Center (TRIC) Universität Münster Albert-Schweitzer-Campus 1 D‐48149 Münster Germany; ^5^ Institut für Materialphysik und CeNTech Universität Münster Wilhelm-Klemm-Str. 10 D‐48149 Münster Germany; ^6^ Instituto de Nanociencia y Nanotecnología CNEA--CONICET Laboratorio de Resonancias Magnéticas Centro Atómico Bariloche 8400 S. C. Bariloche Argentina

**Keywords:** magnetic nanohybrids, magnetic resonance imaging (MRI), nanoparticles, photoluminescence lifetime imaging micro(spectro)scopy (PLIM), phosphorescent Pt(II) complexes, time-resolved microscopy, time-resolved spectroscopy

## Abstract

Two different hybrid nanosystems are prepared by loading highly crystalline, monodisperse magnetite nanocubes (MNCs) with phosphorescent Pt(II) complexes (PtCxs). One involves the encapsulation of the hydrophobic PtCx1 within an amphiphilic comb polymer (MNC@poly(maleic anhydride‐*alt*‐1‐octadecene) [PMAO]–PtCx1), whereas the other involves the direct binding of the hydrophilic PtCx2 to the surface of the MNC mediated by a ligand‐exchange procedure (MNC@OH–PtCx2). Both systems are evaluated as potential candidates for multimodal imaging in magnetic resonance imaging (MRI) and photoluminescence lifetime imaging micro(spectro)scopy (PLIM). PLIM measurements on agarose phantoms demonstrate significantly longer excited‐state lifetimes compared to the short‐lived autofluorescence of biological background. Additionally, both nanosystems perform as effective MRI contrast agents (CAs): the *r*_2_* values are 3–4 times higher than for the commercial CA ferucarbotran. MNC@PMAO–PtCx1 particles also cause significant increases in *r*_2_. While the ligand exchange procedure efficiently anchors PtCxs to the MNC surface, the polymeric encapsulation ensures higher colloidal stability, contributing to differences in PLIM and MRI outcomes. In these results, the successful integration of two complementary noninvasive imaging modalities within a single nanosystem is confirmed, serving as the impetus for further investigation of such systems as advanced multimodal–multiscale imaging agents with dual orthogonal readouts.

## Graphical abstract

Two hybrid nanosystems are prepared by loading magnetite nanocubes with two tailor‐made phosphorescent Pt(II) complexes. The nanohybrids exhibit two orthogonal readouts in agarose phantoms: enhanced magnetic resonance imaging (MRI) contrast compared to ferucarbotran and phosphorescence in the microsecond range, which is a significant advantage when considering biological autofluorescence and using it in phosphorescence lifetime imaging microscopy (PLIM).

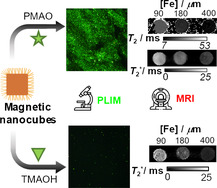

## 1 Introduction

Nowadays, a broad range of nanomaterials is being increasingly used in medicine for various purposes including multimodal bioimaging, diagnosis, prevention, and treatment of diseases.^[^ ^1^ ^]^ Iron‐oxide nanoparticles (NPs) represent an important class of inorganic nanomaterials whose unique physicochemical and magnetic properties, biocompatibility, stability, and low cost make them useful for many medical applications such as hyperthermia, magnetic resonance imaging (MRI), drug delivery, tissue engineering, and bacterial inactivation, among others.^[^ ^2^, ^3^, ^4^, ^5^ ^]^ In this frame, the possibility of developing multifunctional materials based on iron‐oxide NPs is particularly attractive, especially when synergetic effects can be achieved.^[^ ^6^, ^7^, ^8^ ^]^

Superparamagnetic magnetite (Fe_3_O_4_) NPs synthesized by thermal decomposition in organic phases are a type of iron‐oxide NPs that has been vastly studied, in part because of their multiple applications. Thermal decomposition in organic phases yields highly monodisperse and crystalline magnetite NPs stabilized by an oleate capping,^[^ ^9^, ^10^ ^]^ and the precise control of particle size and shape enables a fine‐tuning of the physicochemical properties. As Fe_3_O_4_ NPs have found several applications in aqueous media, among them in vivo imaging, dispersibility in aqueous phases must be enabled. For this purpose, several strategies have been proposed and successfully accomplished. One of them relies on ligand‐exchange methods, where a water‐soluble compound, like tetramethylammonium hydroxide (TMAOH) or 2,3‐dimercaptosuccinic acid, is used to replace the oleate ligands on the NP surface.^[^ ^11^, ^12^ ^]^ Another strategy is the coating of the NPs with amphiphilic comb polymers, a procedure that preserves the oleate capping and involves polymer self‐assembly, which results in the encapsulation of one or more magnetite NPs inside the polymer's hydrophobic domains.^[^ ^13^, ^14^, ^15^ ^]^ In both cases, the resulting hybrid nanomaterials allow the concomitant loading with different molecules, either anchored to their surface or encapsulated within the organic structure.^[^ ^7^, ^16^ ^]^

MRI is one of the most versatile diagnostic tools in medical imaging, providing high‐quality anatomical and functional information.^[^ ^17^ ^]^ This imaging technique is based on the phenomenon of nuclear magnetic resonance of hydrogen atoms. In biological tissues, image contrast depends on the concentration and mobility of H_2_O molecules and their specific chemical environment. Different chemical surroundings will alter the relaxation times of the hydrogen atoms bound in H_2_O molecules.^[^ ^18^ ^]^ To locally manipulate relaxation times and improve the contrast of MR images, two main types of contrast agents (CAs) can be used: a) *T*_1_‐CAs or positive CAs, which reduce longitudinal relaxation time (*T*_1_) increasing signal intensity in *T*_1_‐weighted images, and b) *T*_2_‐CAs or negative CAs, which reduce transversal relaxation time (*T*_2_ and also *T*_2_*) and thus reduce the signal intensity in *T*_2_‐weighted images.^[^ ^5^ ^]^ While Gd^3+^, Mn^2+^, and Dy^3+^ complexes are typical *T*_1_‐CAs, iron‐oxide NPs have been established as *T*_2_‐CAs.

In an MRI experiment, iron‐oxide NPs induce local magnetic field inhomogeneities (susceptibility effects) that increase the transversal relaxation rate (*r*_2_) of ^1^H‐nuclei in their vicinity. The signal generated by these nuclei decays faster than in the absence of iron‐oxide NPs, causing an enhanced contrast in the obtained image.^[^ ^5^ ^]^ Ferucarbotran is a commercially available, iron‐oxide‐NPs‐based CA with high transversal relaxivity, which consists of ≈4 nm magnetite/maghemite crystals coated with carboxydextran.^[^ ^19^ ^]^ The efficiency of iron‐oxide NPs to act as CAs will depend not only on the mean distance between water molecules and the magnetic iron core, but also on the NPs' magnetic properties (magnetic moment and saturation magnetization), which are directly related to the crystallinity, chemical composition, size, and shape.^[^ ^20^, ^21^ ^]^ Moreover, the nature of the organic coating of iron‐oxide NPs strongly influences the interaction of H_2_O molecules with the NPs.^[^ ^5^ ^]^ In this manner, a selective surface modification of the iron‐oxide NPs will additionally enhance their biocompatibility and accumulation in specific tissues, allowing studies of physiological dynamics in vivo by longitudinal MRI measurements.^[^ ^22^, ^23^ ^]^ Therefore, the method used to transfer magnetite NPs to aqueous media could determine their performance as CAs for *T*_2_‐weighted MRI. In this context, highest transverse relaxivities have been reported for iron‐oxide NPs with anisotropic shapes.^[^ ^24^, ^25^ ^]^

In addition, various biological processes can be studied, and in some cases followed in real time and at the subcellular level, by using endogenous or exogenous luminophores in light‐based imaging techniques. Although many different fluorescent molecules have been used as imaging agents, their applicability is frequently limited by self‐quenching, small Stokes shifts, and photobleaching. However, their major disadvantage is represented by their short excited‐state and emission lifetimes (0.1–10 ns), which falls within the same range as the unavoidable tissue autofluorescence (1–5 ns);^[^ ^26^, ^27^ ^]^ this fact together with their small Stokes shifts results in a poor signal‐to‐noise ratio, self‐absorption, and self‐quenching due to back‐scattering from biological samples. In contrast, phosphorescent emitters, such as late‐transition metal complexes, offer good chemical stabilities and improved photophysical properties, including large Stokes shifts, high photoluminescence quantum yields as well as high photostability.^[^ ^28^, ^29^, ^30^ ^]^ Due to the long excited‐state lifetime associated with the phosphorescence arising from the triplet states, their emission can be discriminated from the background autofluorescence of biological materials by time‐gated detection techniques. These properties can be exploited for microsecond‐scale lifetime mapping techniques, such as time‐resolved multiphoton micro(spectro)scopy.^[^ ^31^, ^32^, ^33^, ^34^ ^]^

Among coordination compounds, Pt(II) complexes (**PtCxs**) have recently attracted major attention, due to their appealing photophysical properties including tunable emission wavelength maxima (*λ*_em_, appearing blue‐ or redshifted upon demand‐driven design), high photoluminescence quantum yields (*Φ*_L_) and long excited‐state lifetimes (*τ*).^[^ ^35^ ^]^ For these **PtCx**, the emission of light occurs mainly from metal‐perturbed ligand‐centered triplet states (^3^MP–LC) as admixtures of ligand‐centered (LC, *π*–π*) and metal‐to‐ligand charge‐transfer (MLCT, d–π*) configurations for the monomeric species.^[^ ^36^, ^37^ ^]^ In addition, most of the late transition metal complexes offer a uniquely modular architecture, where the metal center acts as an anchor and different kinds of functionalities can be introduced by ligand engineering in the inner coordination sphere. Thus, the creation of **PtCxs** with desired functional groups to be attached to different nanosystems is feasible. In addition, such triplet emitters can also be implemented as oxygen sensors, as their excited‐state lifetime and luminescence intensity are quenched by diffusion‐controlled Dexter‐like energy transfer to triplet molecular dioxygen (^3^O_2_); in turn, singlet oxygen can be photogenerated, which is used in photodynamic therapy. Recently, we have shown that both **PtCx** ^[^ ^34^, ^35^, ^38^ ^]^ and Re(I)^[^ ^39^ ^]^ complex can potentially perform as oxygen sensors and luminescent labels for biomedical applications.

While MRI offers a field of view in the range of centimeters to meters due to its high tissue penetration,^[^ ^17^ ^]^ high spatial resolution usually requires long acquisition times. In contrast, luminescence‐based microscopy techniques offer excellent sensitivity and spatial resolution at cellular level. Their main drawback, however, is tissue penetration, that limits their use as whole‐body imaging techniques, which nonetheless can be partially overcome by multiphoton excitation.^[^ ^40^ ^]^ Therefore, MRI and luminescence‐based imaging turn out to be complementary noninvasive imaging modalities, and their joint presence in a single CA could eventually result in synergistic addition of multimodal–multiscale imaging capabilities with one single agent. While the combination of both techniques has been proposed earlier,^[^ ^41^, ^42^, ^43^, ^44^ ^]^ including the use of superparamagnetic colloids modified with fluorescent probes,^[^ ^45^ ^]^ to the best of our knowledge, no approach has been based on a single‐hybrid nanosystem built with iron‐oxide NPs loaded with phosphorescent Pt(II)‐based coordination compounds toward combined imaging involving *T*_2_‐weighted MRI and PLIM. Hence, the time‐gated detection and large Stokes shift from triplet emitters can be advantageous to remove background autofluorescence, whereas lifetime imaging maps with spectral resolution provide an optical readout based on two orthogonal observables (lifetime and wavelength). This particular combination of MRI and photoluminescence lifetime imaging micro(spectro)scopy (PLIM) is, to the best of our knowledge, an unexplored field of research. This also opens the gate for multiphoton intravital microscopy, based on the two‐photon excitability of triplet emitters with charge‐transfer excited‐state character.

Thus, we herein propose a convenient design strategy toward multimodal–multiscale imaging agents based on highly crystalline, magnetite nanocubes (MNC) with high *r*_2_(*) relaxivities,^[^ ^24^ ^]^ loaded with two different phosphorescent Pt(II)‐based coordination compounds. Two distinct strategies were used for the preparation of the hybrid nanosystems: one approach is based on the co‐encapsulation with a hydrophobic luminophore using the amphiphilic comb polymer poly(maleic anhydride‐*alt*‐1‐octadecene) (PMAO), whereas the second one involves a ligand‐exchange procedure at the MNC using TMAOH and a hydrophilic **PtCx** bearing three anchoring carboxylate units. The imaging capabilities of both nanosystems were assessed in agarose phantoms as a fundamental preliminary stage, by testing both systems as *T*_1_, *T*_2_, and *T*_2_^*^ CAs for MRI and as PLIM emitters.

## 2 Results and Discussion

### 2.1 Preparation of Hybrid Magnetic NPs Loaded with Pt(II)‐Based Coordination Compounds

The hybrid nanomaterials based on iron‐oxide NPs were designed to merge two orthogonal imaging techniques into a unified imaging agent. In particular, we selected MNCs due to their capability to act as MRI CAs that ensure a high *r*_2_ relaxivity.^[^ ^5^ ^]^ The MNCs were synthesized by a high‐temperature thermal decomposition method, yielding highly crystalline cubic particles and cuboctahedra with variable degree of truncation, with an average edge length of 16 ± 2 nm, as demonstrated by transmission electron microscopy (TEM) (Figure S1, Supporting Information). Furthermore, the fast Fourier transform (FFT) from high‐resolution TEM images confirms the spinel‐ferrite structure, which is associated to magnetite (Fe_3_O_4_) or maghemite (γ‐Fe_2_O_3_) crystal lattices.

In contrast, the superparamagnetic behavior of the nanocubes is confirmed by a negligible coercive field at room temperature (RT), as shown in Figure S1 (Supporting Information). The temperature dependence of the magnetization reveals typical zero‐field‐cooled–field‐cooled (ZFC–FC) curves expected for an ensemble of superparamagnetic NPs. No signs of the Néel temperature of wüstite or the Verwey temperature of Fe_3_O_4_ were found, suggesting that the MNCs are composed of partially oxidized magnetite (Fe_3–*x*_O_4_).^[^ ^46^ ^]^ The saturation magnetization was 62 A m^2^ kg^−1^ at RT and 69 A m^2^ kg^−1^ at 5 K, slightly lower than the values expected for bulk magnetite but within the range usually found in magnetite nanomaterials. Overall, the size of the MNCs ensures a superparamagnetic behavior while their shape is expected to provide high *r*_2_ relaxivities due to local field inhomogeneities associated with their anisotropic shape.

**Figure 1:**
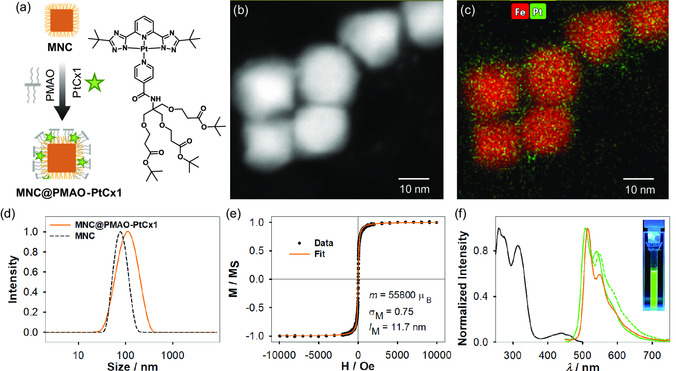
a) Schematic synthetic procedure toward monodisperse magnetite nanocubes (MNC)@poly(maleic anhydride‐*alt*‐1‐octadecene) (PMAO)–PtCx1. b) Scanning transmission electron microscopy high‐angle annular dark‐field (STEM‐HAADF) images and c) energy‐dispersive X‐ray spectroscopy (EDS) elemental mapping of Fe and Pt for MNC@PMAO–PtCx1. d) Size distribution of MNCs (black) and MNC@PMAO–PtCx1 (orange) obtained by DLS. e) Hysteresis loops of MNC@PMAO–PtCx1. f) Normalized excitation spectrum of **PtCx1** (black line) in dichloromethane (DCM) at RT and photoluminescence spectra in DCM at RT for **PtCx1** (green solid line), for MNC@PMAO–PtCx1 in sodium borate buffer (SBB) (orange line) and for **PtCx1** in the amorphous solid state (green dots). Inset: photograph of an MNC@PMAO–PtCx1 suspension in a quartz cuvette irradiated by light with a wavelength of 366 nm.

**Figure 2:**
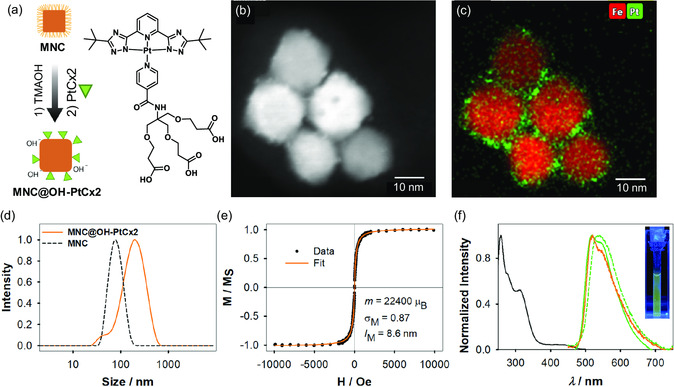
a) Schematic synthetic procedure toward MNC@OH–PtCx2. b) STEM‐HAADF images and c) EDS elemental mapping of Fe and Pt for MNC@OH–PtCx2. d) Size distribution of MNCs (black) and MNC@OH–PtCx2 (orange) obtained by DLS. e) Hysteresis loops of MNC@OH–PtCx2. f) Normalized excitation spectra of **PtCx2** (black line) in DCM at RT and photoluminescence spectra in DCM at RT for **PtCx2** (green solid line), for MNC@OH–PtCx2 in SBB buffer (orange line) and for **PtCx2** in the amorphous solid state (green dots). Inset: photograph of an MNC@OH–PtCx2 suspension in a quartz cuvette irradiated by light with a wavelength of 366 nm.

Transition metal complexes have become increasingly used in bioimaging applications because of the many advantages they present compared to traditional fluorescent emitters. For this reason, two Pt(II)‐based phosphorescent complexes, namely **PtCx1** and **PtCx2**, possessing the same luminophoric core but bearing different terminal groups at the ancillary ligand (as shown in **Figure** **1**a and **2**a) were designed and synthesized (the synthetic procedure and further details regarding the characterization can be found in Supporting Information). The rather unique structure of the previously described **PtCxs** allows their use in hydrophobic environments (due to the presence of the *tert*‐butylester [*t*bu]), and also for direct surface functionalization of the NPs upon hydrolysis of the ester groups, as the three free carboxylic acids in the periphery of the auxiliary ligand of **PtCx2** exhibit high affinity toward oxidic surfaces.

With the aim of loading hydrophobic MNCs with the **PtCxs**, we explored two different strategies, which offer the opportunity to transfer the NPs from organic solvent into aqueous media. The first one, previously addressed by our group,^[^ ^15^, ^47^ ^]^ is based on the assembly of MNCs with the amphiphilic polymer PMAO, which features the encapsulation of the liphophilic **PtCx1** in the hydrophobic domains formed by interdigitation of the comb polymer and the oleate molecules from the MNCs (see scheme in Figure 1).

The other approach relies on a ligand‐exchange procedure in which **PtCx2** is anchored on the surface of the MNCs by the –COOH groups of the auxiliary ligand, involving the previous replacement of oleic acid ligands by hydroxide anions (OH^−^) from the TMAOH phase‐transfer agent (see Figure 2). In this case, the NPs are solely stabilized by electrostatic repulsion, due to the adsorption of negatively charged hydroxide anions. For simplicity, the two different nanosystems are abbreviated as MNC@PMAO–PtCx1 and MNC@OH–PtCx2, respectively.

To ensure that the MNCs were effectively loaded with the Pt(II) compounds, STEM‐HAADF images and EDS spectrum images were acquired, as shown in Figure 1 and 2. For the MNC@PMAO–PtCx1, the Pt EDS signal from the hydrophobic **PtCx1** complexes is homogeneously distributed along the domains formed by the polymer hydrocarbon chains and the oleate capping of the MNC. Instead, for MNC@OH–PtCx2, the Pt signal indicates that **PtCx2** complexes are mostly located on the surface of the MNCs, as a consequence of the replacement of adsorbed OH^−^ ions by the −COO^−^ groups of the **PtCx2** during ligand exchange. These results confirm that both methods enable the formation of nanohybrids consisting of MNCs loaded with Pt complexes.

Next, TXRF measurements were performed to quantify the amounts of Fe and Pt in each nanohybrid. The results allowed us to calculate the number of complexes per surface area of MNC: 1.2 **PtCx1** nm^−2^ for MNC@PMAO–PtCx1 and 3.6 **PtCx2** nm^−2^ for MNC@OH–PtCx2, indicating that the direct covalent binding of the **PtCx** to the surface is more efficient compared to the polymer‐supported encapsulation. Also, for MNC@OH–PtCx2, STEM‐HAADF images suggest that some **PtCx2** molecules arrange in molecular aggregates, probably due to *π*–*π* stacking.^[^ ^48^ ^]^

RT hysteresis loops were obtained for both colloidal nanohybrids and were fitted by considering Langevin functions with a lognormal distribution of magnetic moments. The results indicate an average magnetic moment of 55 800 ± 1100 μ_B_ for MNC@PMAO–PtCx1 and a significantly lower value for MNC@OH–PtCx2 (22 400 ± 400 μ_B_). Such a remarkable difference can be ascribed to the reduction in the apparent magnetic moment due to demagnetizing interactions.^[^ ^15^ ^]^ The latter is a consequence of the aggregation induced by the adsorption of **PtCx2**, which affects the electrostatic double layer after the TMAOH ligand exchange, although an increased oxidation of magnetite to maghemite in MNC@OH–PtCx2 cannot be ruled out.^[^ ^49^ ^]^ Such differences in the colloidal stability of both systems are supported by dynamic light scattering (DLS) measurements. As expected for MNC@PMAO–PtCx1, an average hydrodynamic size of around 120 nm was obtained (Figure 1d), which is larger than those obtained for MNCs in cyclohexane (≈80 nm, Figure 1d and 2d) due to the formation of the hybrid nanoassembly. It is noteworthy that the NPs do not aggregate inside the assemblies. Instead, they are individually wrapped by the hydrocarbon chains of PMAO.^[^ ^15^ ^]^ This is suggested by the increase in the interparticle distance between adjacent cube facets from 1.9 nm for self‐assembled oleate‐capped NPs (Figure S1, Supporting Information) to above 4 nm for the MNC@PMAO–PtCx1 sample (Figure S2, Supporting Information). These results are consistent with previous reports on the formation of NP–polymer assemblies,^[^ ^15^, ^50^ ^]^ which allow a rough estimation of 2–3 nm for the polymer thickness.

In contrast to MNC@PMAO–PtCx1, DLS measurements on MNC@OH–PtCx2 show higher hydrodynamic diameters (≈205 nm) (Figure 2d), due to the loss of the colloidal stability of the particles caused first by the phase transfer and then, and more importantly by the ligand‐exchange procedure to attach the **PtCx2** complex. The latter is supported by results from *ζ*‐potential measurements, used to assess the changes in particle surface charge after loading MNCs with the Pt complexes. While the negative surface charge remained unaltered around −40 ± 10 and −40 ± 20 mV for MNC@PMAO and MNC@PMAO–PtCx1, respectively, a decrease was observed after loading MNCs with **PtCx2**: −51 ± 8 mV for MNC@OH and −17 ± 6 mV for MNC@OH–PtCx2. An absolute value of 30 mV is usually considered as a limit for particle colloidal stability;^[^ ^51^ ^]^ hence, even if the hybrid nanosystem can be dispersed in aqueous solutions, the electrostatic repulsion between the MNC@OH–PtCx2 is not strong enough to prevent some aggregation/agglomeration.

To study the colloidal stability of the systems, *ζ*‐potential and DLS measurements of the hybrid nanomaterials were performed at different pH values and in biologically relevant media (see Figure S3, Supporting Information). In all cases, MNC@PMAO–PtCx1 was more stable than MNC@OH–PtCx2 owing to the noticeable decrease in *ζ*‐potential upon adsorption of **PtCx2** on MNC@OH to yield MNC@OH–PtCx2, as previously mentioned. It should be stressed that while PMAO stabilizes the MNC both by electrostatic (charged carboxylate groups in the polymer backbone) and steric repulsion, TMAOH stabilizes the MNC only by electrostatic effects. Upon **PtCx2** loading, this stabilization is hindered by the surface charge shielding. In the case of **PtCx1**, the complex was loaded in the hydrophobic domains of the 2D structure formed by the hydrocarbon chains of PMAO and oleic acid and therefore neither the electrostatic nor the steric stabilization is affected.

### 2.2 Photophysical Characterization of the Pt(II)‐Based Compounds and the Magnetic Nanosystems

The normalized UV–vis absorption spectra of the **PtCxs** and the nanosystems (both with and without complexes) are shown in Figure S4 (Supporting Information). The absorption spectra of the **PtCxs** show a characteristic vibrational progression as previously reported for related compounds.^[^ ^34^, ^35^, ^52^, ^53^ ^]^ The most intense bands are observed between 217 and 340 nm, which can be assigned to spin‐allowed transitions into LC singlet states (^1^LC) (i.e., *π*–π* configurations), whereas the weaker absorption bands between 340 and 490 nm can be attributed to transitions into singlet excited states related to a mixture of LC and MLCT configurations (Figure S4a,b, green curves, Supporting Information). Instead, NP suspensions of MNC@PMAO–PtCx1 and MNC@OH–PtCx2 (Figure S4a,b, orange curves, Supporting Information) do not show any resolved progression but only a broad band attributed to the absorption and scattering of the magnetic cores, which is also observable for the pure NP samples (Figure S4, black lines, Supporting Information).

Consistently for both complexes, the excitation spectra (Figure 1f and 2f) are in agreement with their respective absorption spectra (Figure S4, Supporting Information). The photoluminescence spectra of the two different complexes and the corresponding NPs in aqueous solution and in the amorphous solid state are depicted in Figure 1f and 2f. The photoluminescence spectra of the complexes in liquid DCM solution at RT showed emission maxima *λ*_max_ = 508 nm for **PtCx1** and *λ*_max_ = 519 nm for **PtCx2**. In the solid state, the emission of the complexes appears slightly redshifted with *λ*_max_ = 514 nm for **PtCx1** and *λ*_max_ = 537 nm for **PtCx2**. The characteristic vibrational progression in the photoluminescence spectra of the complexes can be attributed to the emission from an ^3^MP–LC state, as a combination of ^3^LC and ^3^MLCT character (*π*–π* and d–π* configurations, respectively), as it was previously reported for comparable compounds.^[^ ^34^, ^35^ ^]^ Regarding the photoluminescence spectra of the nanohybrids, a slightly broader emission maximum is found for MNC@PMAO–PtCx1 (*λ*_max_ = 514 nm), while for MNC@OH–PtCx2 the maximum is found at *λ*_max_ = 519 nm, indicating that the emission profile does not vary drastically if compared with the luminescence of **PtCxs** in solution. The combination of these results with the preceding findings confirms a successful loading of both nanohybrids.

Further photophysical data including photoluminescence quantum yields (*Φ*_L_) and excited‐state lifetimes (*τ*) are presented in Table S1 (Supporting Information). In all cases, multiexponential lifetimes were observed, and therefore also the amplitude‐weighted average lifetimes (*τ*_av_amp_) are reported. In general, the complexes in solution can have different thermally accessible conformations which present different deactivation rates; therefore, multiexponential decays are observed. This effect is also noticed in amorphous solids and biological matrices, among others, where different molecular microenvironments are sensed and multiexponential decays are detected.^[^ ^34^, ^54^ ^]^ In macromolecular microenvironments, multiexponential decays are also generally obtained, justifying the use of amplitude‐weighted average lifetimes to estimate radiative and radiationless deactivation rates.^[^ ^55^ ^]^

The hydrolysis of the *t*bu–ester groups does not lead to a significant impact on the photophysical properties of the compounds. In both cases, *τ*_av_amp_ increases upon purging the solution with Ar to remove ^3^O_2_. For **PtCx2**, the excited‐state lifetime is almost doubled upon Ar purging, whereas for **PtCx1** the lifetime is prolonged by a factor of three. In the case of amorphous solids, the excited‐state lifetimes are comparable with those in Ar‐purged solutions. A similar trend can be observed for the photoluminescence quantum yields. As shown in Table S1 (Supporting Information), a small change in the structure of both complexes does not have a significant impact on their photophysical properties. For **PtCx1**, we measured an amplitude‐weighted average photoluminescence lifetime (*τ*_av_amp_) of 138 ns with *Φ*_L_ = 0.02 in aerated DCM (within the instrumental uncertainty, i.e., <2%), while upon deaeration, they reached a *τ*_av_amp_ = 398 ns with *Φ*_L_ = 0.25. In contrast, for **PtCx2,** we observed a *τ*_av_amp_ of 126 ns with *Φ*_L_ = 0.04 in air‐equilibrated samples, and a *τ*_av_amp_ = 247 ns with *Φ*_L_ = 0.11 in Ar‐purged solutions. Regarding the characterization of the amorphous solid, we observed a *τ*_av_amp_ = 392 ns with *Φ*_L_ = 0.12 for **PtCx1**, and a *τ*_av_amp_ = 308 ns with *Φ*_L_ = 0.08 for **PtCx2**. All these results show that lifetimes and quantum yields are longer in deoxygenated liquid samples (or in the amorphous solid) compared to those in aerated DCM solutions, proving that the emission of the complex is significantly quenched by molecular dioxygen, in agreement with phosphorescence from excited triplet states. As for the emission lifetimes, an enhancement was observed for both nanosystems in comparison with the complexes in solution, with similar values both in air‐equilibrated and in Ar‐purged samples: *τ*_av_amp_ = 782 ns (air) versus *τ*_av_amp_ = 761 (Ar) for MNC@PMAO–PtCx1; *τ*_av_amp_ = 801 ns (air) and *τ*_av_amp_ = 895 ns (Ar) for MNC@OH–PtCx2. This result proves that binding to the NPs at least partially avoids physical quenching due to interactions with H_2_O and ^3^O_2_. This outcome also shows that these materials suppress the photoproduction of cytotoxic singlet oxygen (^1^O_2_). In addition, the photophysical properties of MNC@PMAO and MNC@OH were also studied. No photoluminescence signals were found from the systems without the respective **PtCxs** (data not shown).

The previously shown results therefore motivated the evaluation of the nanohybrids as multimodal agents as MRI CAs and PLIM emitters.

### 2.3 PLIM

**Figure 3:**
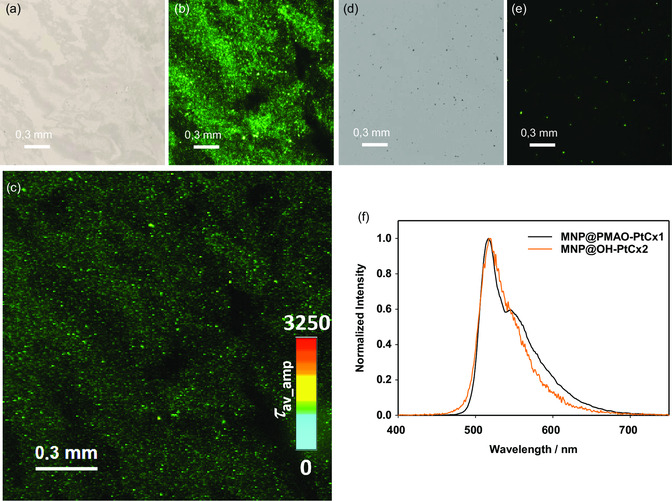
a) Bright‐field images, b) photoluminescence microscopy image, and c) photoluminescence lifetime map of MNC@PMAO–PtCx1 in 1% agarose. d) Phase‐contrast image and e) photoluminescence microscopy image of MNC@OH–PtCx2 in 1% agarose. f) Normalized photoluminescence spectra of the particles in the phantom gels (*λ*_ex_ = 375 nm) measured using a photoluminescence spectrometer coupled to a confocal microscope (*λ*_ex_ = 375 nm, low‐pass (LP) cut‐off filter 514 LP).

To assess the capabilities of the assemblies for time‐resolved photoluminescence micro(spectro)scopy, phantoms made of agarose gels were loaded with both nanosystems and subsequently studied by means of PLIM; hence, luminescence microscopy images, photoluminescence lifetime maps, and spatially resolved emission spectra were obtained and evaluated. As can be observed, MNC@PMAO–PtCx1 are evenly distributed all over the gel, as evidenced by their strong and homogenous green luminescence (**Figure** **3**a,b). In contrast, for MNC@OH–PtCx2, only discrete aggregates with lower luminescence intensity were seen (Figure 3d,e). In addition, the lifetimes of the complexes in the nanomaterials incorporated into the gels were measured by PLIM (Figure 3c), yielding a homogeneous amplitude‐weighted average photoluminescence lifetime map with long phosphorescence decays (*τ*_av_amp_ = 1.00 ± 0.03 μs). Emission spectra were also measured for different regions of the gels, giving a consistent spectral profile in agreement with MNC@PMAO–PtCx1 in water dispersion (Figure 3f). These results evidence that the intrinsic photophysical properties of this nanosystem are not affected upon immobilization in the agarose phantoms, as no significant differences in excited‐state lifetimes and/or photoluminescence spectra are observed. In contrast, for MNC@OH–PtCx2, only discrete aggregates with lower emission intensity were observed (Figure 3d,e); thus, while it was possible to obtain the spectra of the particles in agarose (Figure 3f), their photoluminescence lifetime maps could not be measured reliably as their brightness was too sparse to enable a reasonable signal‐to‐noise ratio.

These results indicate that MNC@PMAO–PtCx1 constitutes an interesting nanoassembly for photoluminescence‐based microscopy techniques, not only due to the homogeneous distribution, but also owing to the preserved photophysical properties in the phantoms. Moreover, the greatest advantage is the long lifetime of the excited state of the complex in the nanohybrid, which is at least two orders of magnitude longer if compared with cellular autofluorescence (typically below 10 ns).

### 2.4 MRI

To evaluate the ability to act as magnetic CAs, MRI experiments were performed with agarose phantoms containing either MNC@PMAO–PtCx1, MNC@OH–PtCx2, or the commercially available MRI CA ferucarbotran (manufactured under the trademark Resovist), which consists of iron‐oxide NPs coated with carboxydextran.

High‐resolution images of individual phantoms revealed a homogeneous distribution in sample cross‐sections for MNC@PMAO–PtCx1, whereas individual spots of signal voids were identified for MNC@OH–PtCx2 as a result of their agglomeration inside the phantoms (Figure S5c,d, Supporting Information). These results are in agreement with PLIM experiments (Figure 3) and with the lower colloidal stability of such samples, as discussed before (vide supra). Samples containing only 1% agarose display homogeneously distributed signal intensities in their cross sections, with only a few, dispersed signal voids (Figure S5, Supporting Information) that show the minimal presence of trapped air following our sample preparation.

**Figure 4:**
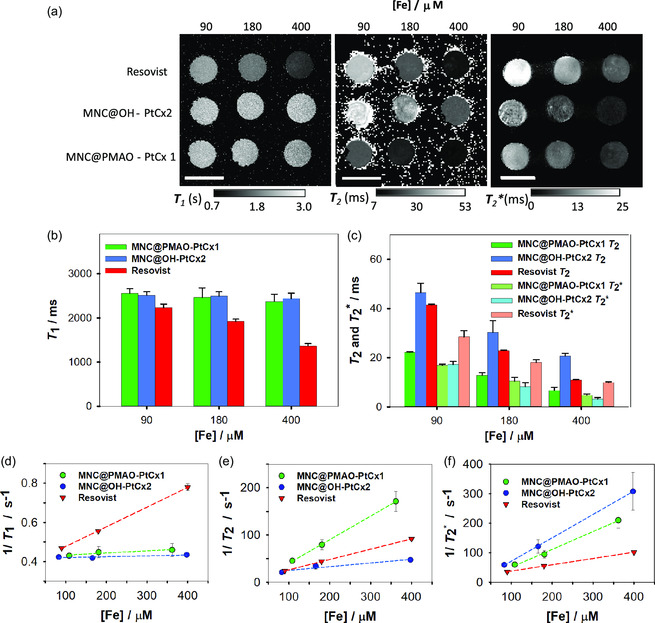
a) Maps of relaxation times *T*_1_, *T*_2_, and *T*_2_*. Scale bars: 1 cm. The displayed maps are partly stitched together from multiple scans but all panels have been adjusted to the same windowing. b) *T*_1_ relaxation times. c) *T*_2_ and *T*_2_* relaxation times. d–f) Relaxivities *r*_1_, *r*_2_, and *r*_2_*, respectively. Relaxivities are represented by the slopes of the linear regressions. The exact [Fe] values in (a–c) correspond to those of Resovist (see Table S2 for further details, Supporting Information). Values are means ± standard deviation.

Maps for relaxation times and relaxivities for the different samples are presented in **Figure** **4** and Table S3 (Supporting Information). MRI confirmed lower signals with increasing [Fe] for all compounds, especially in the case of *T*_2_*‐weighted images (Figure 4a).

A detectable influence of [Fe] on *T*_1_ relaxation times was only confirmed for Resovist, with *r*_1_ = 0.933 L mmol^−1^ s^−1^. Instead, MNC@PMAO–PtCx1 and MNC@OH–PtCx2 did not show any marked effect on *T*_1_ relaxation times (*r*_1_ = 0.105 and 0.042 L mmol^−1^ s^−1^, respectively). This finding agrees with the result obtained by Dash et al., who reported similar findings on 15 nm diameter iron cores at 9.4 T.^[^ ^56^ ^]^ Tuning the core size and the hydrodynamic diameter of iron‐oxide NPs coated with carboxydextran were shown to reduce the impact of [Fe] on *T*_1_ while maintaining short *T*_2_(*).^[^ ^57^ ^]^ In line with these reports, large hydrodynamic diameters of MNC@PMAO–PtCx1 and aggregated MNC@OH–PtCx2 are responsible for low *r*_1_.

In comparison to ferucarbotran, our hybrid magnetic nanomaterials magnetic nanosystems especially shortened *T*_2_* (Figure 4, Table S3, Supporting Information): *r*_2_* for MNC@PMAO–PtCx1 and MNC@OH–PtCx2 were 3–4 times larger. MNC@PMAO–PtCx1 particles also caused severe reductions in *T*_2_, with *r*_2_ more than twice as high as ferucarbotran. This in turn had an *r*_2_ three times higher than MNC@OH–PtCx2 particles, which only had weak impact on *T*_2_ at the measured concentrations.

Magnetic properties remain the defining characteristic of the particles to affect MRI relaxation times and thus determine their ability to act as CAs. These properties depend on many factors, most importantly size, shape, chemical composition, and aggregation. While iron‐oxide NPs can be used as positive CAs for *T*_1_‐weighted MRI with ultrashort *T*_E_,^[^ ^58^ ^]^ the near‐zero *r*_1_ of MNC@PMAO–PtCx1 and MNC@OH–PtCx2 leads to a high *r*_2_/*r*_1_ relaxivity ratio. Both magnetic hybrid systems can therefore be classified as exclusive *T*_2_ CAs, without any *T*_1_‐enhancing effects at 9.4 T. They are also much more effective *T*_2_ CAs than Resovist if simply judged by their *r*_2_/*r*_1_ ratios.^[^ ^19^ ^]^ The high transversal relaxivities *r*_2_ of MNC@PMAO–PtCx1 particles not only exceed the effects of Resovist but rather lie in the range of dysprosium‐ or holmium‐based NPs reported for 400–600 L mmol^−1^ s^−1^ at 9.4 T^[^ ^59^, ^60^ ^]^ and are comparable to other highly efficient *T*_2_ CAs based on ferrite NPs reported before.^[^ ^24^, ^25^ ^]^

Key differences between our systems and Resovist include distinct particle sizes (16 vs 4.2 nm), shape (cubes vs spheres), crystallinities (high vs low), and the coating (which is carboxydextran in the case of Resovist^[^ ^19^ ^]^). The well‐defined iron‐oxide core and the MNCs aggregation can be regarded as the main features for reducing transversal relaxation times in MRI, signified by their high *r*_2_*.^[^ ^61^ ^]^ As mentioned before, different degrees of aggregation have been confirmed by DLS, PLIM, and MRI. The strong adsorption of **PtCx2** on the MNCs in MNC@OH–PtCx2 lowers the surface charge due to the displacement of OH^−^ ions by –COO^−^‐anchored complexes of **PtCx2**. This hinders the electrostatic stabilization of the colloid and promotes the formation of dense aggregates, as evidenced by the large hydrodynamic values found in DLS measurements (Figure 2). Densely packed, multicore aggregates cause severe local susceptibility artifacts, leading to greatly enhanced *r*_2_* for these particles. In comparison, the functionalization of MNCs with PMAO yields stable assemblies (≈120 nm hydrodynamic diameter), where individual MNCs are separated by a few nanometers within an organic matrix.^[^ ^15^ ^]^ Interestingly, even such individual MNCs in PMAO‐based nanohybrids produce pronounced *r*_2_* effects compared to ferucarbotran.

The similarity of *r*_2_ and *r*_2_* for MNC@PMAO–PtCx1, as well as the respective discrepancy in relaxivities for MNC@OH–PtCx2, can be explained by different diffusion regimes around the NPs in solution. These are determined mainly by their hydrodynamic diameter, which likely places MNC@PMAO–PtCx1 in the static dephasing regime with high *r*_2_ and high *r*_2_* (*T*_2_* limit). The larger hydrodynamic size of MNC@OH–PtCx2 NPs leads to an echo‐limited regime, where *r*_2_ becomes smaller, with unaffectedly, high *r*_2_*.^[^ ^62^, ^63^ ^]^ The coating itself may also impact on transversal relaxation rates, since protons with different dephasing may exchange between carboxyl groups exposed by PMAO‐coated NPs and the H_2_O pool. These chemical exchange processes are, however, difficult to disentangle from overall water diffusion around the iron‐oxide NPs. Individual roles would need to be tested in future experiments.

Altogether, our findings provide experimental evidence of the multifunctional response of MNC@PMAO–PtCx1 and MNC@OH–PtCx2 nanohybrids. Although the amount of complex loaded in the latter system is higher, hysteresis loops showed that their magnetic moment is lower than the value obtained for MNC@PMAO–PtCx1, mainly due to interparticle interactions arising from particle aggregation after the absorption of **PtCx2**, but also because of magnetite oxidation, as a result of the phase transfer procedure. DLS and *ζ*‐potential measurements (Figure S3, Supporting Information) also agree with the results from magnetic measurements, confirming that, in the case of MNC@PMAO–PtCx1, MNCs inside the assemblies are individually coated by the hydrocarbon chains of PMAO, whereas for MNC@OH–PtCx2 the results suggest particle aggregation. Consequently, MNC@PMAO–PtCx1 presents superior colloidal stability, which agrees with PLIM and MRI results. Instead, all experiments with MNC@OH–PtCx2 showed particle aggregation in the agarose phantoms and a lower efficiency in photoluminescence emission. In addition to colloidal stability and Pt‐complex loading, the NP capping may also impact on the stability of the MNCs. For example, Lartigue and coworkers^[^ ^50^ ^]^ showed that the NP coating modulates their biodegradation in lysosomal‐type media, and the MNCs encapsulated with PMAO were more resistant to this process than MNCs functionalized with poly(ethylene glycol)–gallol.

Moreover, the surface chemistry of the nanohybrids and the chemical stability of both the iron‐oxide cores and the **PtCxs** are factors that condition the possible toxicity of these nanosystems, a key issue for their applications both in vitro and in vivo. In this regard, viability assays have been performed for different cell lines and demonstrated that MNP@PMAO are not cytotoxic for Fe concentrations up to 1750 mm, which are considerably higher than those used in the present PLIM and MRI experiments.^[^ ^64^, ^65^ ^]^ PMAO‐coated iron‐oxide NPs with similar size have been successfully applied for in vivo imaging.^[^ ^66^ ^]^ Iron‐oxide NPs with similar surface charge to MNC@OH–PtCx2 displayed low cytotoxicity and in vivo MRI contrast enhancement.^[^ ^67^ ^]^ In any case, the superior *r*_2_ and *r*_2_* values compared to ferucarbotran could reduce the necessary volume per kg body weight to achieve contrast enhancement, therefore reducing potential side effects.^[^ ^68^ ^]^ As for the **PtCxs**, a structurally analogous complex has been adsorbed in a different nanosystem, and subsequent investigations have demonstrated the absence of cytotoxicity at the highest concentration employed (4.5 mm Pt).^[^ ^48^ ^]^ Notably, this concentration is similar to that of the **PtCx1** complex in the 400 μm Fe sample, which is the highest used in the phantoms in our studies. Despite this, the potential toxicity of **PtCx1** would be further diminished by the fact that it is encapsulated within the hydrophobic domains of the MNC@PMAO nanosystem.

Even though some multifunctional materials have been suggested for combining the two noninvasive imaging modalities described in this work, to the best of our knowledge, this is the first report where MNCs are loaded with **PtCxs** for combining *T*_2_‐weighted MRI with photoluminescence‐based imaging techniques. Previous studies are usually based on highly paramagnetic Gd(III) complexes (*T*_1_ CAs).^[^ ^41^, ^42^, ^43^, ^44^ ^]^ Iron‐oxide NPs are environmentally friendly and could become an alternative to Gd(III) complexes. Iron uptake and accumulation after intravenous injection has been previously reported for mouse brain, kidney, liver, and spleen, with highest iron accumulation in liver and spleen (≈10 mm).^[^ ^23^ ^]^ The iron concentrations used in the presented study are far below these peak values and rather correspond to iron uptake in kidney (900 μm) or brain tissue (80 μm). Thus, new compounds may open new opportunities for *T*_2_(*)‐weighted contrasts in tissues with relatively low iron uptake, thanks to the compounds’ high *T*_2_ and especially *T*_2_* relaxivities.

## 3 Conclusions

Two different multifunctional assemblies based on superparamagnetic iron‐oxide nanocubes were prepared. For the loading of the MNCs with the phosphorescent Pt(II)‐based coordination compounds, two molecular designs (**PtCx1** and **PtCx2**) were employed, which present different binding properties, owing to the new monodentate ligand structures.

The photophysical characterization showed that both architectures present large Stokes shifts and long photoluminescence lifetimes. It should be highlighted that the excited‐state lifetimes of the nanohybrid materials are in the range of 760–900 ns, which is a substantial advantage when it comes to suppression of the short‐lived background autofluorescence in biological samples, if compared with commonly used fluorophores. Another important result is the similar lifetimes observed in aerated and in Ar‐purged solutions, suggesting that the complexes are less prone to diffusional quenching by H_2_O and O_2_ when they are in the nanohybrid assemblies.

Additionally, the magnetic characterization showed that MNC@OH–PtCx2 possesses less average magnetic moment, a consequence of particle aggregation, and an increasing oxidation of magnetite after the ligand‐exchange procedure. This result was in concordance with DLS measurements, where a higher hydrodynamic diameter for MNC@OH–PtCx2 compared to MNCs in cyclohexane was found.

Luminescence imaging techniques with the nanohybrids in agarose phantoms showed better results for MNC@PMAO–PtCx1, yielding a homogeneous distribution all over the samples’ extent and good photoluminescence intensity, with a *τ*_av_amp_ = 1.00 μs. On the other hand, for MNC@OH–PtCx2, inhomogeneous particle distribution with discrete luminescent spots inside the phantoms were observed, due to the high agglomeration of the particles. This indicates that MNC@PMAO–PtCx1 presents consistent photophysical properties also in agarose phantoms, a gel matrix that mimics certain properties of biological tissues, making the nanosystem a potential candidate for future biological applications.

MRI experiments showed that, unlike the commercial CA Resovist (ferucarbotran), MNC@PMAO–PtCx1 and MNC@OH–PtCx2 did not show any effect on *T*_1_ relaxation times, as expected for their size. In contrast, MNC@PMAO–PtCx1 and MNC@OH–PtCx2 cause substantial reductions in *T*_2_*, with the latter performing better. This is due to the fact that MNC@OH–PtCx2 generates multicore aggregates that significantly improve *r*_2_*. In contrast, only MNC@PMAO–PtCx1 causes substantial reductions in *T*_2_* and *T*_2_.

In conclusion, the study highlights the potential of our nanosystems, particularly MNC@PMAO–PtCx1, as interesting candidates for noninvasive imaging applications. By effectively integrating two complementary imaging modalities within a single nanovehicle, these nanosystems enable simultaneous micro‐ and macroscopic observations through two orthogonal readouts: phosphorescence and magnetic contrast, respectively. These findings pave the way for future advancements in in vitro and in vivo imaging techniques.

## Conflict of Interest

The authors declare no conflict of interest.

## Author Contributions

M.B.R.A and T.M.K.: conceptualization, investigation (synthesis, photophysical and structural characterization), formal analysis; G.C.L.: conceptualization, investigation (synthesis and magnetic characterization), formal analysis; E.W.L.: investigation (magnetic characterization); B.M. and S.K.: investigation (agarose phantoms, MRI measurements); B.M. and C.F: formal analysis (MRI data); I.M.: investigation (PLIM), formal analysis; S.O. and G.W.: investigation (TEM measurements); A.H.: investigation (NMR measurements); M.H. and U.K.: investigation (μXRF measurements); C.V., C.A.S.: conceptualization, supervision, project administration; C.A.S.: funding acquisition; M.B.R.A, T.M.K, G.C.L., B.M., I.M., C.F., C.V., C.A.S.: Writing and revision–original draft and final version.

## Supporting information

Supplementary Material

## Data Availability

The data that support the findings of this study are available in the supplementary material of this article.
