# Supplementary material for: Superparamagnetic Nanoparticles with Phosphorescent Complexes as Hybrid Contrast Agents: Integration of MRI and PLIM
Source: Small Sci. 2024 Jan 12;4(3):2300145. doi: 10.1002/smsc.202300145 (PMC11935033; doi:10.1002/smsc.202300145)
Supplement: Supplementary file 1 — Supplementary Material [file SMSC-4-2300145-s001.pdf]

Supporting Information

Superparamagnetic Nanoparticles with Phosphorescent Complexes as Hybrid Contrast Agents: Integration of MRI and Time-Resolved Photoluminescence Lifetime Imaging Micro(spectro)scopy

*Rivas Aiello, Maria Belen[1][2]¥; Kirse, Thomas M.[2][3]¥; Lavorato, Gabriel C.[1]; Maus, Bastian[4]; Maisuls, Iván[2][3]; Kuberasivakumaran, Shivadharshini [4]; Maisuls, Iván[2][3]; Ostendorp, Stefan[5]; Hepp, Alexander[3]; Holtkamp, Michael[3]; Winkler; Elin L.[6]; Karst, Uwe[3]; Wilde, Gerhard[5]; Faber, Cornelius[4]; Vericat, Carolina[1]; Strassert, Cristian A.[2][3]**

[1] Instituto de Investigaciones Fisicoquímicas Teóricas y Aplicadas (INIFTA), Universidad Nacional de La Plata – CONICET, 1900 La Plata, Buenos Aires, Argentina.

[2] CeNTech, CiMIC, SoN, Westfälische Wilhelms-Universität Münster, Heisenbergstraße 11, D-48149 Münster, Germany.

[3] Institut für Anorganische und Analytische Chemie, Westfälische Wilhelms-Universität Münster, Corrensstraße 28/30, D-48149 Münster, Germany.

[4] Klinik für Radiologie, Translational Research Imaging Center (TRIC), Westfälische Wilhelms-Universität Münster, Albert-Schweitzer-Campus 1, D-48149 Münster, Germany.

[5] Institut für Materialphysik und CeNTech, Westfälische Wilhelms-Universität Münster, Wilhelm-Klemm-Str. 10, D-48149 Münster, Germany.

[6] Instituto de Nanociencia y Nanotecnología, CNEA--CONICET, Laboratorio de Resonancias Magnéticas, Centro Atómico Bariloche, 8400 S. C. Bariloche, Argentina.

E-Mail: ca.s@wwu.de

¥These authors contributed equally to this work

Table of Contents

Section 1: Materials and methods	S3
Section 2: Synthesis and characterization of **PtCx1**, **PtCx2** and ligand precursors	S10
Section 3: Synthesis and characterization of MNC	S16
Section 4: Photophysical properties	S20
Section 5: MRI measurements	S23
Section 6: NMR and mass spectra of **PtCx1**, **PtCx2** and ligand precursors	S26
Section 7: Time-resolved photoluminescence decays and fitting parameters	S40
Section 8: References	S45

Section 1: Materials and methods

1.1 Materials

Iron(III) acetylacetonate ($Fe(acac)_3$, 99%) and tetramethylammonium hydroxide pentahydrate (TMAOH, 98%) were obtained from ACROS. Oleic acid (90%), sodium oleate (82%), benzyl ether (98%), 1-tetradecene (92%), 1-octadecene (90%), poly(maleic anhydride-alt-1-octadecene) (PMAO, Mn=30.000-50.000), were purchased from Sigma Aldrich. Silica gel 60 (particle size 35-70 µm, 230-400 mesh) for column chromatography was purchased from Merck (mentioned as silica) and used, unless otherwise stated.

1.2. NMR-Spectroscopy and Mass-spectrometry

NMR spectra were obtained at the Institut für Anorganische und Analytische Chemie (WWU), using a Bruker AVANCE NEO/ Bruker AVANCE I/ Bruker AVANCE III (400 MHz) or a Bruker AVANCE NEO (500 MHz). All measurements were performed at 300 K unless mentioned otherwise. The ^{1}H-NMR and ^{13}C-NMR chemical shifts (δ) of the signals are given in parts per million and are referenced to the residual signal of the deuterated solvent. The signal multiplicities are abbreviated as follows: s, singlet; d, doublet; t, triplet; q, quartet; m, multiplet. ^{1}H NMR chemical shifts are given relative to TMS and are referenced to the solvent signal. Spectra of other nuclides like ^{13}C or ^{19}F are referenced according to the proton resonance of TMS as the primary reference for the unified chemical shift scale.[1] The signal multiplicities are abbreviated as follows: s, singlet; d, doublet; t, triplet; q, quartet; m, multiplet. All coupling constants (J) are given in Hertz (Hz). Exact mass (EM) determination by mass spectrometry (MS) was carried out at the Organisch-Chemisches Institut Münster (WWU) using a LTQ Orbitap LTQ XL (Thermo-Fisher Scientific, Bremen) with electron spray injection (ESI). Attenuated total reflection

1.3 Photophysical measurements

Absorption spectra were measured with a Shimadzu UV-3600 I plus UV-VIS-NIR spectrophotometer. Photoluminescence quantum yields were measured with a Hamamatsu Photonics absolute PL quantum yield measurement system (C9920-02) equipped with a L9799-01 CW Xe light source (150 W), a monochromator, a C7473 photonic multi-channel analyzer, an integrating sphere and employing U6039-05 software (Hamamatsu Photonics, Ltd., Shizuoka, Japan).

Steady-state excitation and emission spectra were recorded on a FluoTime 300 spectrometer from PicoQuant equipped with: a 300 W ozone-free Xe lamp (250-900 nm), a 10 W Xe flash-

lamp (250-900 nm, pulse width *ca.* 1 μs) with repetition rates of 0.1 – 300 Hz, double excitation monocromators (Czerny-Turner type, grating with 1200 lines/mm, blaze wavelength: 300 nm), diode lasers (pulse width < 80 ps) operated by a computer-controlled laser driver PDL-828 "Sepia II" (repetition rate up to 80 MHz, burst mode for slow and weak decays), two double-grating emission monochromators (Czerny-Turner, selectable gratings blazed at 500 nm with 2.7 nm/mm dispersion and 1200 lines/mm, or blazed at 1200 nm with 5.4 nm/mm dispersion and 600 lines/mm) with adjustable slit width between 25 μm and 7 mm, Glan-Thompson polarizers for excitation (after the Xe-lamps) and emission (after the sample). Different sample holders (Peltier-cooled mounting unit ranging from -15 to 110 °C or an adjustable front-face sample holder), along with two detectors (namely a PMA Hybrid-07 from PicoQuant with transit time spread FWHM < 50 ps, 200 – 850 nm, or a H10330C-45-C3 NIR detector with transit time spread FWHM 0.4 ns, 950-1700 nm from Hamamatsu) were used. Steady-state spectra and photoluminescence lifetimes were recorded in TCSPC mode by a PicoHarp 300 (minimum base resolution 4 ps) or in MCS mode by a TimeHarp 260 (where up to several ms can be traced). Emission and excitation spectra were corrected for source intensity (lamp and grating) by standard correction curves. For samples with lifetimes in the ns order, an instrument response function calibration (IRF) was performed using a diluted Ludox® dispersion. Lifetime analysis was performed using the commercial EasyTau 2 software (PicoQuant). The quality of the fit was assessed by minimizing the reduced chi squared function (χ^2) and visual inspection of the weighted residuals and their autocorrelation.

All solvents used were of spectrometric grade (Uvasol®, Merck). Solutions of **PtCx1** and **PtCx2** were prepared in dichloromethane (DCM) and purged by bubbling for 30 min with DCM-saturated Ar ($c = 10^{-5}$ M).

1.4 Confocal fluorescence and PLIM micro(spectro)scopy

The agarose phantoms with the highest iron concentration (*i.e.*, [Fe]) of each particle (202.0 mg/L for MNP@PMAO-PtCx1 and 92.5 mg/L for MNP@OH-PtCx2, see Table S2) were carefully re-heated in a H_2O bath until the entire sample was melted. Before drop-casting, the corresponding samples were ultra-sonicated at 80 °C to ensure a homogeneous distribution. 20 μL droplets were then poured on microscope slides (Menzel Gläser SuperFrost Plus, Thermo Scientific, Braunschweig, Germany) and covered with cover slips. The slides were stored at 4 °C before being used for microscopy.

The agarose dispersion with the highest NP concentration contains 42.0 μM or 55.6 μM of Pt for MNC@PMAO-PtCx1 or MNC@OH-PtCx2, respectively. At an average of 20 μL solution

per slide, each slide should contain 0.84 nmol or 1.11 nmol Pt (MNC@PMAO-PtCx1 or MNC@OH-PtCx2, respectively).

Photoluminescence lifetime imaging microscopy (PLIM) was recorded on a fluorescence microscope (IX 73 from Olympus) with a complete confocal system and a laser combining unit (LCU), an inverted microscope body and a multichannel detection unit (MicroTime 200, PicoQuant) equipped with diode lasers (providing adjustable output power and repetition rates up to 80 MHz inside a compact fibre couple unit with wavelengths between 375 and 900 nm). For beam diagnostics, a charge-couple device (CCD) camera and a photodiode were available in the main optical unit (MOU) of the microscope. The MOU is equipped with two detectors, namely a hybrid photomultiplier-based single photon counting module (PMA Hybrid 40, PicoQuant) and a SPAD-based photon counting module (SPCM-AQR-14, Perkin-Elmer). Different band-pass (BP) and low-pass (LP) filters were placed before these detectors on demand to acquire lifetime maps. Data acquisition is based on the unique time-tagged time-resolved (TTTR) measurement mode, where simultaneous data acquisition on two channels is possible. Data were processed and analysed with the SymphoTime 64 (PicoQuant) software. In order to couple the MicroTime 200 and the FluoTime 300 instruments, a fiber coupler was employed. In this way, the spectrometer can be used to record either steady-state or time-resolved luminescence spectra and decays from a sample mounted on the microscope. Luminescence micrographs were acquired using the same microscope mentioned above, equipped with a X-CiteQ Lamp module (Excelitas Technologies) as excitation source and a UI-5580SE (IDS) digital camera. Different band pass (BP) and low pass (LP) cubes were using accordingly.

1.5 Structural characterization of the Nanohybrids

Size, morphology, crystal structure and chemical composition of the MNC, MNC@PMAO-PtCx1 and MNC@OH-PtCx2 were characterized by transmission electron microscopy (TEM) experiments using a ThermoFisher Scientific FEI Themis 300 G3 transmission electron microscope (TEM) equipped with a high brightness field emission gun (X-FEG), a monochromator, an image Cs-corrector, a quadrupole energy-dispersive, X-ray spectroscopy (EDS) system (Super-X EDX detector), a high-angle annular dark-field (HAADF) detector (Fischione model 3000) and a fast CMOS camera (Ceta, 4k x 4k). The microscope was operated at an acceleration voltage of 300 kV. 15 μL of diluted sample solutions were drop-casted on TEM grids (holey carbon films on 200 mesh copper grids, Plano GmbH) and allowed to dry for

24 hours before the measurement. The size distribution of the MNCs was analyzed by measuring the edge length of around 120 particles and constructing number-weighted histograms. Average sizes were obtained by assuming lognormal distribution of edge lengths (a): $f(a) = (\sqrt{2\pi}\sigma a)^{-1} \mathrm{e}^{\frac{-\ln^2\left(\frac{a}{a_0}\right)}{2\sigma^2}}$, where a_0 is the median edge length and σ the deviation. The particle size ($< a > \pm SD$) is then reported according to the mean edge length $< a >= a_0 \mathrm{e}^{\sigma^2/2}$ and the standard deviation $SD =< a > \sqrt{\mathrm{e}^{\sigma^2} - 1}$.

Dynamic light scattering (DLS) measurements were performed to investigate the colloidal stability of the particles. For each sample, average values from three independent measurements of hydrodynamic size distributions were obtained in a Malvern Nano ZS apparatus at 25 °C with a scattering angle of 173°. MNCs samples were prepared in cyclohexane solution, while MNC@PMAO-PtCx1 and MNC@OH-PtCx2 were prepared in SBB and H_2O, respectively. ζ potential measurements were also determined using the Malvern Nano ZS apparatus using a DTS1070 folded capillary cell.

1.6 Iron and platinum quantification

Total reflection X-ray fluorescence analysis (TXRF) was carried out in a S2-PICOFOX instrument (Bruker Nano, Berlin, Germany) with an air-cooled molybdenum anode for X-ray generation to determine the amount of Fe and Pt in the samples. The excitation settings were set to 50 kV and 750 µA and quartz glass disks were used as sample carriers. An arsenic solution with a concentration of 10 mg/L was used as internal standard.

A sample volume of 100 µL was mixed with the same volume of the 10 mg/L arsenic standard solution. 5 µL aliquots of MNC@PMAO-PtCx1 and MNC@OH-PtCx2 samples were placed on the sample carriers and evaporated to dryness. The signals of iron ($K_{\alpha 1}$ = 6.405 keV) and platinum ($K_{\alpha 1}$ = 9.442 keV) were used for quantification whereas the signal of arsenic ($K_{\alpha 1}$ = 10.543 keV) was used as internal standard. The analysis was performed by signal integration over 500 s.

1.7 Magnetic characterization

Dried powders of oleate-capped hydrophobic MNCs were pressed in gelatin capsules for magnetic measurements. Magnetization *vs*. temperature curves were acquired by measuring zero-field cooled (ZFC)-field-cooled (FC) curves between 5 and 310 K with an applied field of 50 Oe in a SQUID magnetometer (MPMS Quantum Design). Hysteresis curves were measured in the same equipment by applying fields up to ±30 kOe at 5 K and 295 K. To obtain

magnetization values expressed per mass of iron oxide, they were corrected by considering the organic mass fraction (≈30 wt%) obtained from thermogravimetric analysis in a Shimadzu DTG-60H apparatus. Measurements were performed by heating ≈2 mg of hydrophobic MNCs in an alumina crucible up to 600 ºC under Ar atmosphere and the results are shown in Figure S1.

RT hysteresis loops of MNC@PMAO-PtCx1 and MNC@OH-PtCx2 colloids (0.2-0.4 g_{Fe}/L) were measured in a vibrating sample magnetometer (VSM Lakeshore 7300) by placing 30 µL in a glass holder and applying fields up to ±10 kOe. Diamagnetic signals from the sample holder (-7.33·10-8 emu/Oe) and from the dispersion medium (≈-6·10-8 emu/Oe) were subtracted and the normalized hysteresis curves were fitted by assuming a lognormal distribution of magnetic moments that follow Langevin functions.[2,3]

1.8 Magnetic resonance imaging

In vitro MRI phantoms were prepared in 1% agarose (w/v; Carl Roth, Karlsruhe, Germany) at pH between 5 and 7. For both nanosystems, different nanoparticle concentrations were assayed: 90, 180 and 400 µM (expressed as [Fe] concentrations). The dissolution of agarose in pure H_2O was facilitated using magnetic stirrers and heating plates. Phantoms were prepared in 2 mL Eppendorf tubes. The stock solution was vortexed and sonicated for approximately 15 min before mixing it with agarose to ensure a homogeneous particle distribution in the aqueous stock solution. To compare the nanosystems with an established iron oxide-based MRI contrast agent, agarose phantoms of Resovist (ferucarbotran, Bayer AG, Leverkusen, Germany) were prepared in the same way and stored at 4 °C before and after scanning. (Table S2).

Since air trapped in the phantoms appears similar to iron oxide particles in T_2*-weighted images, an additional set of pure agarose phantoms was treated with ultrasound to remove residue air while the agar was still warm and liquid. The samples appeared visually similar to the untreated tubes presented and yielded nearly identical relaxation times (not shown). It can thus be concluded that the applied protocol does not contaminate MRI phantoms with air to a degree that impacts the measurement of relaxation times.

Magnetic resonance imaging was performed on a 9.4 T horizontal bore animal scanner (Bruker BioSpec 94/20; Bruker BioSpin, Ettlingen, Germany), equipped with a 720 mT/m actively shielded gradient system. Samples were placed manually in the center of the scanner and their position was confirmed with tri-pilot localizer scans after adjustments of basic frequency, pulse power and magnetic field homogeneity. Individual samples were scanned with a cryogenic

mouse head coil, while multiple samples were scanned with a linear birdcage resonator. All MRI measurements were performed at RT (20 °C).

T_1 relaxation times (longitudinal relaxation) were measured with a spin echo sequence with variable repetition times (RAREVTR; T_E = 6.5 ms, RARE factor = 2, T_R = 200, 400, 800, 1500, 3000, 5500 ms; 256×256 matrix). T_2 relaxation times (transversal relaxation) were measured with a spin echo sequence with variable echo times (MSME; T_{Emin} = 6.5 ms, echo spacing = 6.5 ms, 16 echoes, T_R = 2000 ms; 128×128 matrix). T_2* relaxation times (transversal relaxation under local magnetic field disturbances) were measured with an ultra-short T_E sequence to account for the rapid signal decay in samples with high [Fe] (UTE; T_{Emin} = 0.45 ms; T_{Emax} = 40 ms; T_R = 120 ms; flip angle = 20°; 256×256 matrix). A single slice (0.7 mm thickness) was used for all relaxation time measurements. When necessary, the field of view was adjusted to accommodate multiple sample tubes. In-plane resolution was usually <0.2 mm isotropic. Maps of relaxation times where calculated in the scanner operating software ParaVision 6.0.1.

Signal intensities of a sample tube cross section were retrieved from magnitude images (ImageJ 1.53) and plotted against T_R (for T_1) or T_E (for T_2 and T_2*). T_1 was calculated by fitting the signal intensity (SI) vs. T_R with

$$SI = A + C \cdot \left(1 - e^{-\frac{T_R}{T_1}}\right) \quad [1]$$

while T_2 and T_2* were calculated by fitting the SI vs. T_E of the MSME or UTE sequences, respectively, with

$$SI = A + C \cdot e^{-\frac{T_E}{T_{2(*)}}} \quad [2]$$

where:

A = absolute bias/offset

C = signal intensity/proton density

Fitting was done using the nonlinear regression function of SPSS (v28, IBM, Armonk, NY, USA). Estimates in SPSS were based on sequential quadratic programming with a step limit of 2 and default optimality tolerance and function precision.

A compound's relaxivity is related to its MRI contrast ability. Thus, based on relaxation times (T_1, T_2, T_2*) determined at different particle concentrations in agarose gel, the different relaxivities (r_1, r_2, r_2* in L mmol^{-1} s^{-1}) were calculated from weighted-least-squares linear regressions in SPSS with

$$r_i = \frac{\frac{1}{T_i} - A}{c} \quad [3]$$

where:

T_i = respective relaxation time, either T_1, T_2 or T_2* in s

A = offset (s^{-1})

c = [Fe] in mmol L^{-1}

Section 2: Synthesis and characterization of PtCx1, PtCx2 and ligand precursors

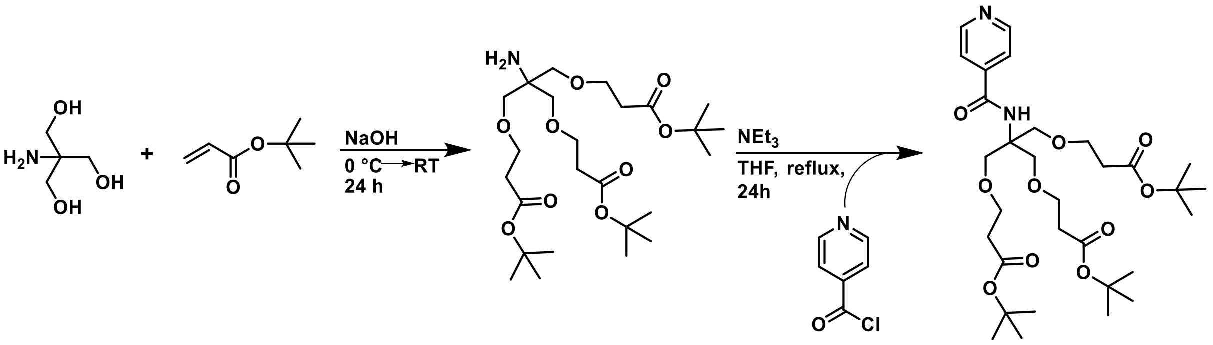

Scheme S1. Synthetic procedure for the monodentate ligand precursor (**2**).

2.1 Synthesis of tris{[2-(*tert*-butoxycarbonyl)ethoxy]methyl}methylamine (1)

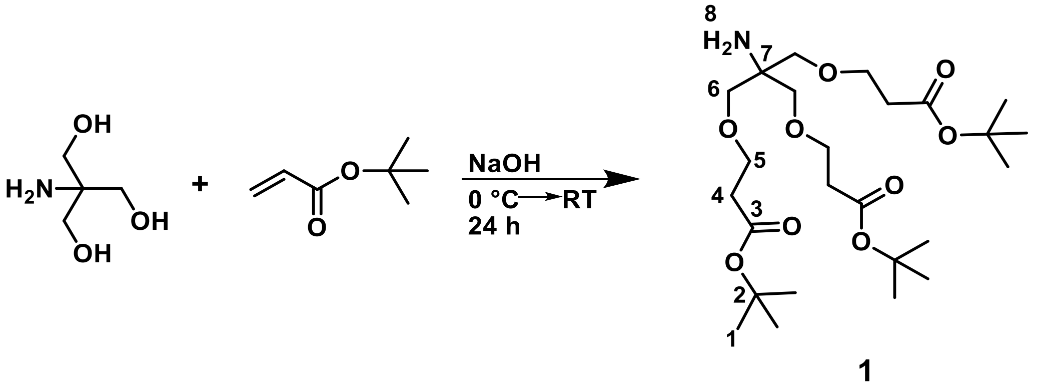

1

1 was synthesized by a modified literature procedure.[4] Briefly, to a solution of tris(hydroxymethyl)aminomethane (tris) (3.63 g, 30 mmol, 1 eq.) in DMSO (6 ml), NaOH (aq. 5 M, 0.6 mL) was added under Ar atmosphere. Afterwards, the solution was frozen (~0°C) and *tert*-butyl acrylate (13.05 mL, 102 mmol, 15 mL, 3.4 eq.) was added dropwise. The solution was allowed to warm to room temperature naturally for overnight. The next day, the water was removed under reduced pressure and the crude product was purified by column chromatography (EtOAc/CyH, 0/4 -3/2 + 0.05 vol% NH_4OH). The product was obtained as colorless oil (3.90 g, 7.71 mmol, 77%).

^{1}H-NMR (400 MHz, $CDCl_3$): δ (ppm) = 3.61 (t, $^{3}J_{HH}$ = 6.4 Hz, 6H, H5), 3.28 (s, 6H, H6), 2.41 (t, $^{3}J_{HH}$ = 6.4 Hz, 6H, H4), 1.60 (s, 2H, H8), 1.41 (s, 27H, H1).

^{13}C-NMR (101 MHz, $CDCl_3$): δ (ppm) = 170.8 (C3), 80.3 (C2), 72.8 (C6), 67.1 (C5), 55.9 (C7), 36.3 (C4), 28.0 (C1).

^{15}N-NMR (41 MHz, $CDCl_3$): δ (ppm) = 29 (N8).

EM-MS-ESI (MeOH, $C_{25}H_{47}NO_9$, m/z): calcd. for $[C_{25}H_{47}NO_9+H]^+$ = 506.33236, found for $[\mathbf{1}+H]^+$ = 506.33199.

2.2 Synthesis of di-*tert*-butyl 3,3'-((2-((3-(*tert*-butoxy)-3-oxopropoxy)methyl)-2-(isonicotinamido)propane-1,3-diyl)bis(oxy))dipropionate (2)

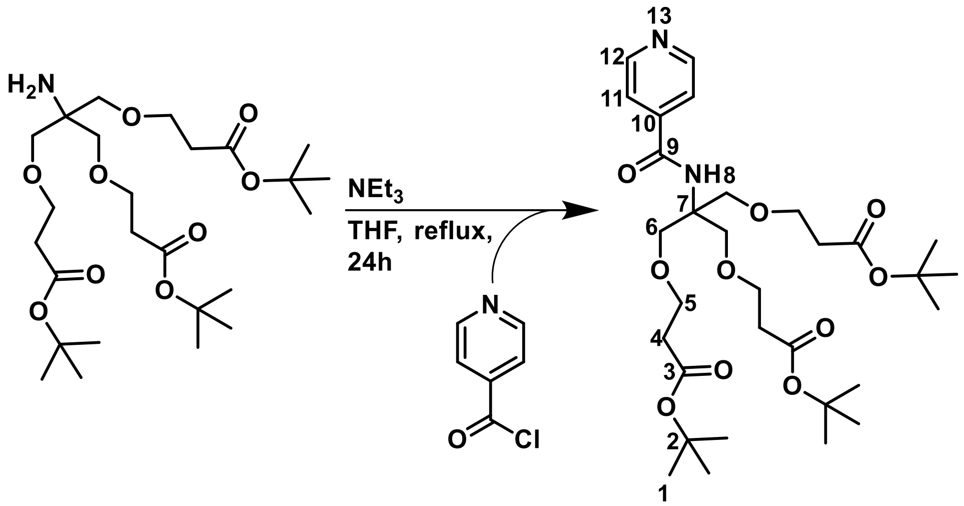

1 2

In an oven dried SCHLENK tube, isonicotinic acid (5.84 g, 4.75 mmol, 1.2 eq.) was dissolved in thionylchloride (4.71 g, 39.6 mmol, 2.87 ml, 10 eq.) under Ar atmosphere. After refluxing for 48 h, the thionylchloride was evaporated *in vacuo.* The resulting residue was dissolved in THF (dry, 20 ml), triethylamine (0.80 g, 7.91 mmol, 1.1 ml, 2 eq.) as well as **1** (2.00 g, 3.96 mmol, 1.0 eq.) were added and the solution was refluxed for 24 h. Afterwards, the solvent

was evaporated under reduced pressure and the crude product was purified by column chromatography (CyH/EtOAc, 10:0-6:4). The product was obtained as colorless oil (2.18 g, 3.56 mmol, 90%).

^{1}H-NMR (400 MHz, $CDCl_3$): δ (ppm) = 8.66 (m, 2H, H12), 7.64 (m, 2H, H11), 6.82 (s, 1H, H8), 3.80 (s, 6H, H6), 3.65 (t, $^{3}J_{HH}$ = 6.2 Hz, 6H, H5), 2.42 (t, $^{3}J_{HH}$ = 6.2 Hz, 6H, H4), 1.37 (s, 27H, H1).

^{13}C-NMR (101 MHz, $CDCl_3$): δ (ppm) = 170.8 (C3), 165.5(C9), 150.2(C12), 142.3(C10), 121.2 (C11), 80.4 (C2), 69.8 (C6), 67.0 (C5), 60.3 (C7), 36.0 (C4), 28.0 (C1).

^{15}N-NMR (41 MHz, $CDCl_3$): δ (ppm) = 319 (N13), 114 (N8).

EM-MS-ESI (MeOH, $C_{31}H_{50}N_2O_{10}$, m/z): calcd. for $[C_{31}H_{50}\ N_2O_{10}+Na]^+$ = 633.33577, found for [**2**+Na]$^+$ = 633.33588.

2.3 Synthesis of PtCx1

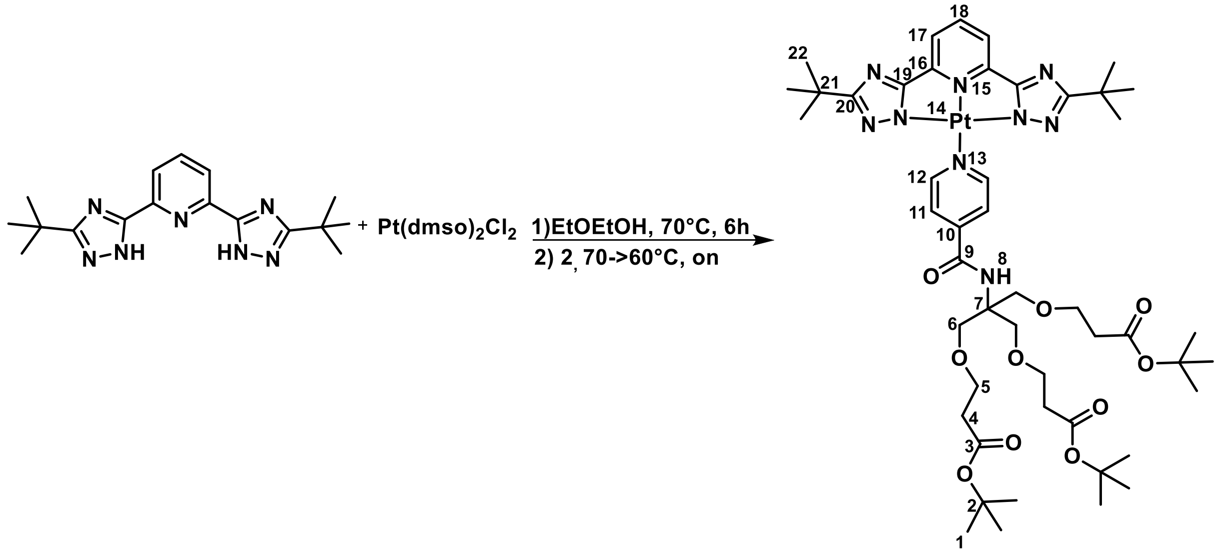

PtCx1

2,6-bis(3-(*tert*-butyl)-1*H*-1,2,4-triazol-5-yl)pyridine (53.28 mg, 0.164 mmol, 1 eq.) and $Pt(dmso)_2Cl_2$ (69.14 mg, 0.164 mmol, 1.0 eq) were dissolved in 2-ethoxyethanol (10 ml). The solution was subsequently deoxygenated with Ar for 10 min and stirred at 70°C for 6 h. After lowering the temperature to 60°C **2** (100 mg, 0.164 mmol, 1 eq.) was added and the solution was further stirred at 60°C for overnight. The crude product was precipitated by addition of H_2O and was further purified by colomn chromatography (CyH/EtOAc, 10:0 - 4:6). The product was obtained as yellow solid (90.7 mg, 0.08 mmol, 49%).

^{1}H-NMR (500 MHz, CD_2Cl_2): δ (ppm) = 10.33 (m, 2H, H12), 7.92 (m, 2H, H11), 7.85 (t, $^3J_{HH}$ = 8.0 Hz, 1H, H18), 7.62 (t, $^3J_{HH}$ = 8.0 Hz, 2H, H17), 7.10 (s, 1H, H8), 3.85 (s, 6H, H6), 3.70 (t, $^3J_{HH}$ = 6.1 Hz, 6H, H5), 2.46 (t, $^3J_{HH}$ = 6.1 Hz, 6H, H4), 1.43 (s, 18H, H22), 1.39 (s, 27H, H1).

^{13}C-NMR (126 MHz, CD_2Cl_2): δ (ppm) = 171.8 (C3), 171.3 (C20), 164.6 (C9), 163.0 (C19), 155.1(C12), 150.8(C16), 145.1 (C10), 142.9 (C18), 124.3 (C11), 116.3(C17), 80.8 (C2), 69.3 (C6), 67.6 (C5), 61.4 (C7), 36.5 (C4), 33.7 (C21), 30.2 (C22), 28.3(C1).

^{15}N-NMR (51 MHz, CD_2Cl_2): δ (ppm) = 208 (N15), 206 (N13), 116 (N8).

^{195}Pt-NMR (86 MHz, CD_2Cl_2): δ (ppm) = -2802 (Pt14).

EM-MS-ESI (MeOH, $C_{48}H_{71}N_9O_{10}Pt$, m/z): calcd. for $[C_{48}H_{71}N_9O_{10}Pt+Na]^+$ = 1151.48671, found for [**PtCx1**+Na]$^+$ = 1151.48624.

2.4 Synthesis of PtCx2

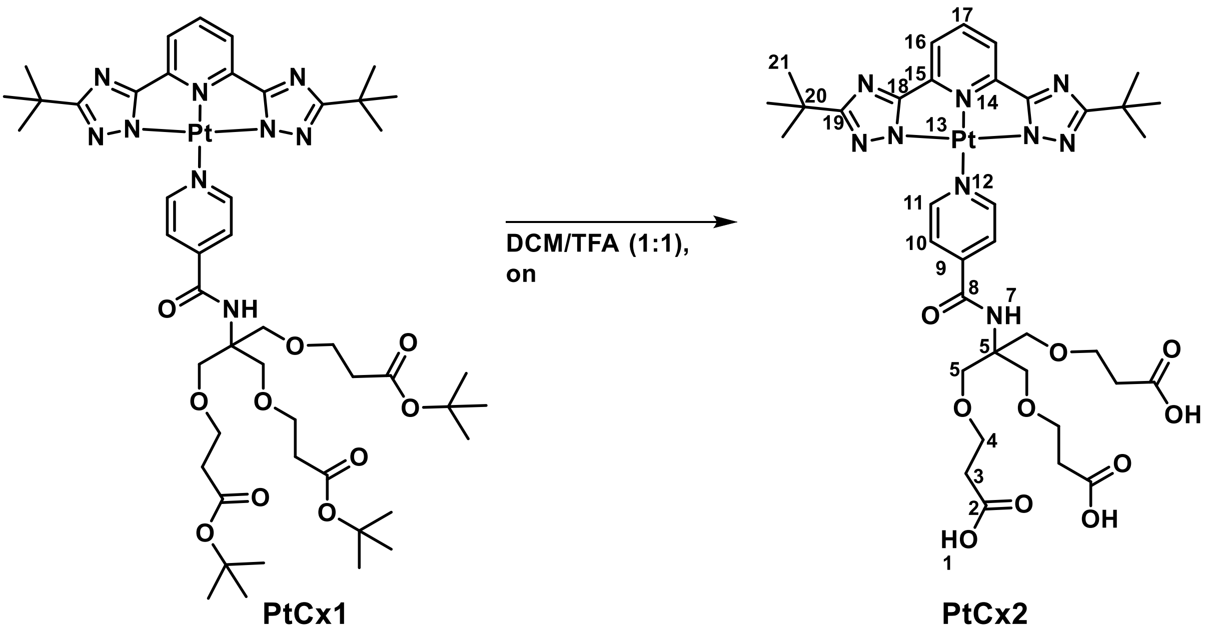

A solution of **PtCx1** (35 mg, 0.0031 mmol, 1 eq.) was prepared in DCM (5 mL); after deoxygenating the solution for 5 min by bubbling with Ar, an equivolumetric amount of trifluoroacetic acid was added and the solution was further deoxygenated for 5 min. After stirring overnight, the solvent was evaporated under reduced pressure. The resulting solid was dried *in vacuo* to obtain **PtCx2** as yellowish solid (29.78 mg, 0.031 mmol, quantitative 100%).

^{1}H-NMR (400 MHz, DMF-d_7): δ (ppm) = 10.15 (m, 2H, H11), 8.21 (t, $^3J_{HH}$ = 7.9 Hz, 1H, H17), 8.11 (m, 2H, H10), 7.74 (t, $^3J_{HH}$ = 7.9 Hz, 2H, H16), 3.88 (s, 6H, H5), 3.76 (t, $^3J_{HH}$ = 6.1 Hz, 6H, H4), 2.57 (t, $^3J_{HH}$ = 6.1 Hz, 6H, H3), 1.44 (s, 18H, H21).

^{13}**C-NMR** (101 MHz, DMF-d_7): δ (ppm) = 173.3 (C2), 171.2 (C19), 164.9 (C8), 163.0 (C18, overlap.), 154.8 (C11), 150.2 (C15), 145.9 (C9), 144.7 (C17), 125.0 (C10), 117.2 (C16), 68.8 (C5), 67.8 (C4), 62.1 (C6), 35.3 (C3), 33.8 (C20), 30.1 (C21).

^{195}**Pt-NMR** (86 MHz, DMF-d_7): δ (ppm) = -2801 (Pt13).

EM-MS-ESI (MeOH, $C_{36}H_{47}N_9O_{10}Pt$, m/z): calcd. $[C_{36}H_{47}N_9O_{10}Pt\text{-}H]^-$ = 959.30232, found for [**PtCx2**-H]$^-$ = 959.29982.

Section 3: Synthesis and characterization of MNC, MNC@PMAO-PtCx1 and MNC@OH-PtCx2

3.1 Synthesis of iron oxide nanocubes

A modified literature procedure was employed for the synthesis of monodisperse oleate-capped hydrophobic iron oxide nanocubes (MNCs).[5] $Fe(acac)_3$ (1.7 mmol), sodium oleate (0.75 mmol) and oleic acid (5 mmol) were dissolved in 1-octadecene (10 mL), 1-tetradecene (3 mL) and benzyl ether (10 mL). The mixture was placed in a round-bottom flask equipped with a condenser and heated at 60 °C for 1 h under vacuum. Afterwards, the temperature was raised to 290 °C with a heating rate of 15 °C/min and the mixture was kept at that temperature for one hour. The entire procedure was conducted under a constant Ar flow and magnetic stirring. As soon as the solution was cooled down to room temperature (RT), the MNCs were precipitated with isopropanol and acetone, separated via centrifugation (10 min, 2000 rcf), resuspended in chloroform, washed again twice with isopropanol and finally once with ethanol. Afterwards, the particles were resuspended in cyclohexane, and this was used as the stock MNCs solution.

3.2 Synthesis of MNC@PMAO-PtCx1

In this approach, a phase-transfer method based on the formation of nanoparticle-polymer assemblies was used.[6,7] Briefly, MNCs (7 mg), PMAO (25 mg, 85 monomer units/nm^2) and **PtCx1** (7.08 μmol) were dispersed in 25 mL of chloroform and left under stirring in the absence of light. After 60 min, the solvent was evaporated under reduced pressure to obtain a dried film at the bottom of the flask. Afterwards, 10 mL of sodium borate buffer (SBB) (50 mM, pH = 9) were added and the mixture was sonicated at 60 °C for 2.5 h until the particles were completely dispersed. To purify the product, the obtained colloid was centrifuged twice (5 min, 2700 rcf) to eliminate any large aggregates in the pellet. Then, the supernatant was centrifuged twice for 20 min at 14000 rcf and the resulting pellet was finally resuspended in SBB to obtain a stock solution of MNC@PMAO-PtCx1.

3.3 Synthesis of MNC@OH-PtCx2

TMAOH was first used to remove the oleic acid capping layer and to transfer the NPs to aqueous media by a ligand-exchange procedure.[8,9] To this end, MNCs (20 mg) were mixed with TMAOH (110 mg) in ethanol (1.5 mL), sonicated for 60 min, washed twice with ethanol and finally centrifuged (10 min, 2800 rcf). The particles were then mixed overnight at 60 °C with 10 mL of a solution of **PtCx2** in ethanol (8.33 nM) and washed by magnetic decantation with H_2O several times (using a Nd-based permanent magnet) to obtain aqueous colloids of MNC@OH-PtCx2, which were stored at 4 ºC until further used.

Blank samples without the Pt(II) complexes (MNC@PMAO and MNC@OH), were prepared following the same procedure described above.

3.4 MNC characterization

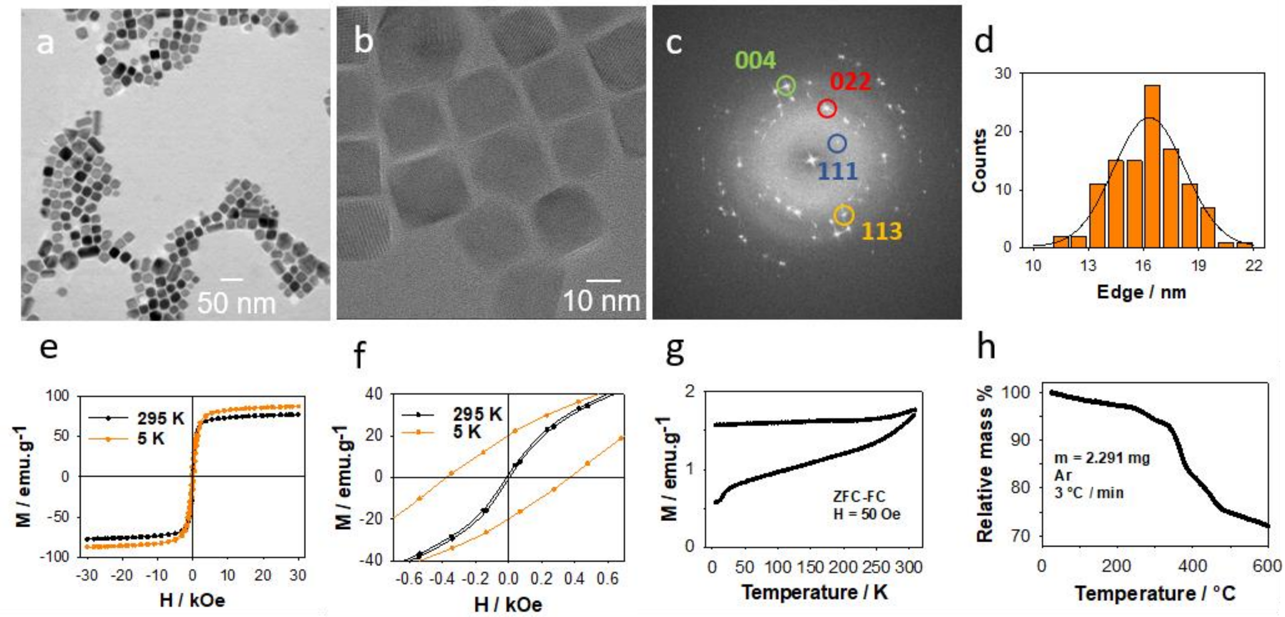

Fig S1. (a) TEM image (b) HR-TEM image, (c) fast-Fourier-transform (FFT) image indexed with reflections from spinel ferrite (Fd-3m space group), (d) size distribution with lognormal fit, (e) (f) hysteresis loops, (g) zero field cooled and field-cooled temperature dependence of the magnetization, and (h) thermogravimetric (TG) curves of oleate-capped MNCs.

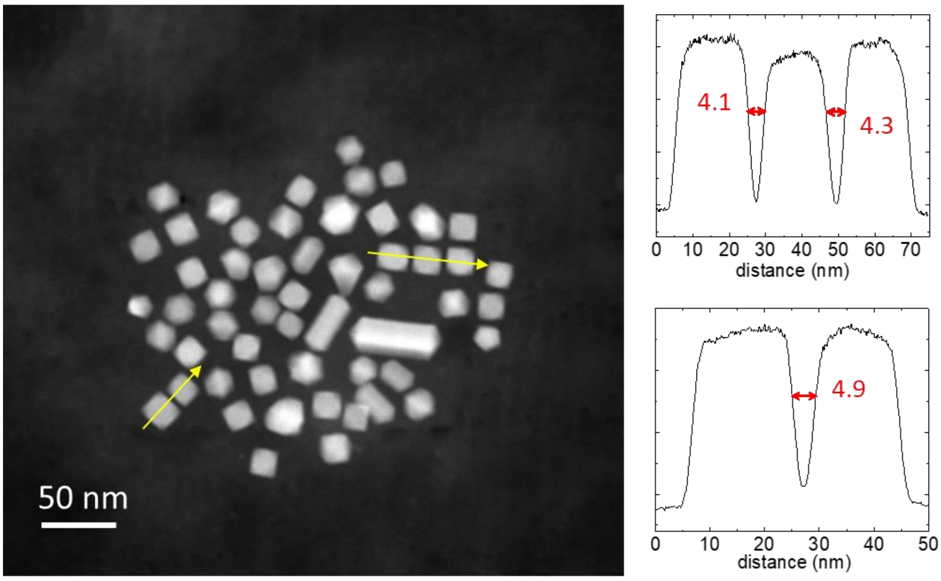

Fig S2. STEM-HAADF image of MNC@PMAO-PtCx1 showing minimum interparticle distances above 4 nm.

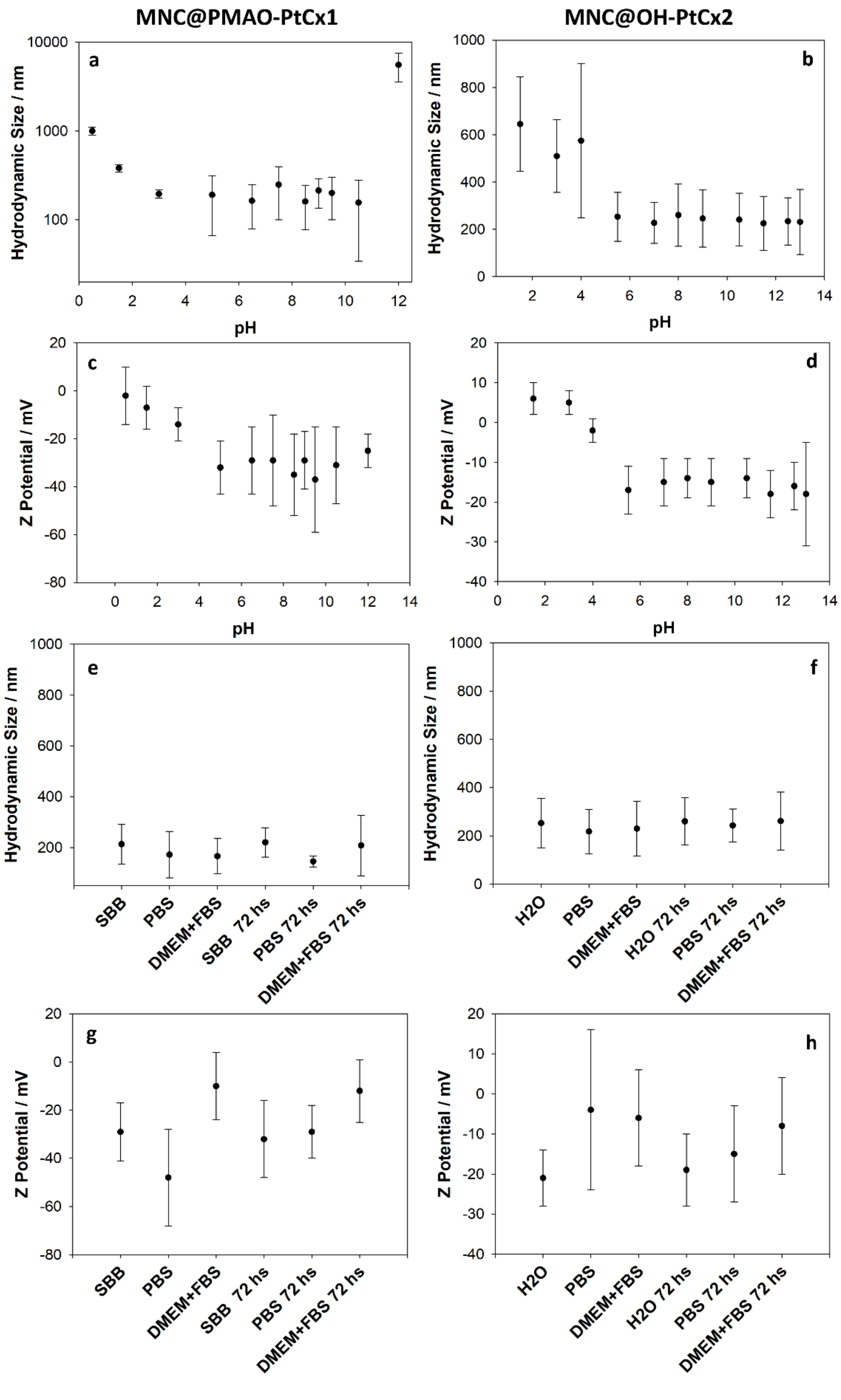

Figure S3: DLS and Z potential measurements of MNC@PMAO-PtCx1 (panels a and c) and MNC@OH-PtCx2 (panels b and d) at different pHs in the synthesis media. DLS and Z potential measurements of MNC@PMAO-PtCx1 (panels e and g) and MNC@OH-PtCx2 (panels f and h) in different biologically-relevant media immediately after mixing and after 72 hours.

Section 4: Photophysical properties

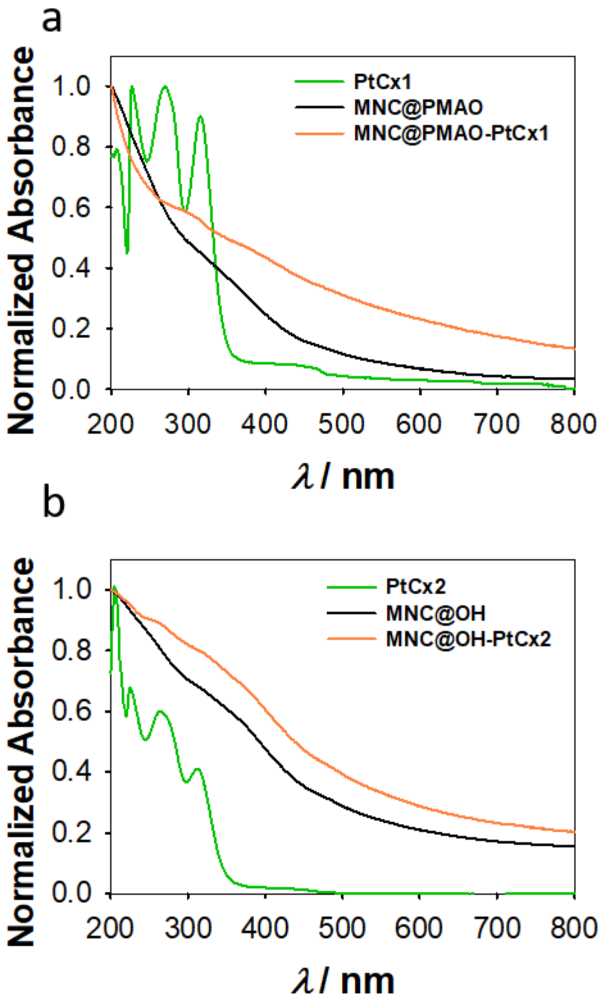

Fig S4. Normalized UV- vis absorption spectra at RT for: a) **PtCx1** in DCM, MNC@PMAO and MNC@PMAO-PtCx1 in SBB buffer; b) **PtCx2** in DCM, MNC@TMAOH and MNC@OH-PtCx2 in H_2O.

Table S1. Photophysical properties of **PtCx1**, **PtCx2**, MNC@PMAO-PtCx1 and MNC@OH-PtCx2 in fluid solution at RT and in the solid state (amorphous powder) at room temperature. For multi-exponential photoluminescence decays, the amplitude-weighted average lifetimes (τ_{av_amp}) are also shown along with the individual components and their respective weighing factors. Raw time-resolved photoluminescence decays and fitting parameters are shown in the S31-S40.

	**τ_L / ns**		**Φ_L ± 0.02**	
	**Air**	**Ar**	**Air**	**Ar**
**PtCx1**<br>**in DCM solution**	τ_1 = 371.7 ± 0.7 (36%)<br>τ_2 = 18 ± 3 (7%)<br>τ_3 = 3.7 ± 0.4 (57%)<br>τ_{av_amp} = 138 ± 13	τ_1 = 1880 ± 40 (4%)<br>τ_2 = 1121± 8 (28%)<br>τ_3 = 6.4 ± 0.8 (68%)<br>τ_{av_amp} = 400 ± 40	0.02	0.25
**PtCx1 amorphous solid**	τ_1 = 1660 ± 40 (10%)<br>τ_2 = 620 ± 30 (26%)<br>τ_3 = 181 ± 11 (27%)<br>τ_4 = 30.8 ± 0.7 (37%)<br>τ_{av_amp} = 392 ± 5	-	0.12	-
**MNC@PMAO-PtCx1**<br>**in SBB buffer solution**	τ_1 = 1900 ± 500 (11%)<br>τ_2 = 880 ± 140 (55%)<br>τ_3 = 300 ± 130 (25%)<br>τ_4 = 60 ± 30 (9%)<br>τ_{av_amp} = 782 ± 9	τ_1 = 2000 ± 200 (8%)<br>τ_2 = 910 ± 60 (53%)<br>τ_3 = 350 ± 60 (28%)<br>τ_4 = 76 ± 16 (11%)<br>τ_{av_amp} = 761.1 ± 1.8	< 0.02	-

**PtCx2 in DCM solution**	$\tau_1 = 611.4 \pm 3.6$ (17%)<br>$\tau_2 = 177 \pm 4$ (9%)<br>$\tau_3 = 6.84 \pm 0.13$ (74%)<br>$\tau_{av\_amp} = 126 \pm 6$	$\tau_1 = 1643 \pm 19$ (13%)<br>$\tau_2 = 408 \pm 18$ (9%)<br>$\tau_3 = 51 \pm 15$ (5%)<br>$\tau_4 = 4.5 \pm 0.2$ (73%)<br>$\tau_{av\_amp} = 247 \pm 13$	0.04	0.11
**PtCx2 amorphous solid**	$\tau_1 = 1860 \pm 30$ (8%)<br>$\tau_2 = 578 \pm 14$ (19%)<br>$\tau_3 = 152 \pm 7$ (28%)<br>$\tau_4 = 23.8 \pm 1.4$ (45%)<br>$\tau_{av\_amp} = 308 \pm 3$	-	0.08	-
**MNC@OH-PtCx2 in aqueous solution**	$\tau_1 = 3000 \pm 1300$ (12%)<br>$\tau_2 = 800 \pm 300$ (24%)<br>$\tau_3 = 152 \pm 18$ (64%)<br>$\tau_{av\_amp} = 800 \pm 90$	$\tau_1 = 7000 \pm 1000$ (5%)<br>$\tau_2 = 618 \pm 103$ (22%)<br>$\tau_3 = 165 \pm 11$ (57%)<br>$\tau_4 = 2200 \pm 400$ (16%)<br>$\tau_{av\_amp} = 895 \pm 17$	< 0.02	-

Section 5: MRI measurements

Table S2. Iron-concentration ([Fe]) in 1% agarose MRI phantoms. Samples in the row named **2/3/4** were used for the comparison of relaxation times, due to their similar [Fe].

	**MNP@PMAO-PtCx1**		**MNP@OH-PtCx2**		Resovist®
Solution	[Fe] / mg/L	[Fe] / µM	[Fe] / mg/L	[Fe] / µM	[Fe] / µM
Stock	404.0	7234	185.0	3313	500000
**1**	202.0	3617	92.5	1656	
**2**	20.2	**362**	22.2	**398**	**400**
**3**	10.1	**181**	9.3	**166**	**180**
**4**	6.1	**109**	4.7	**83**	**90**

Table. S3. Relaxation times and relaxivities of different contrast agents. [Fe] are grouped for Resovist concentrations (see bold values in Table S2). All scans were performed on 1% agarose phantoms at 9.4 T field strength. Values for relaxation times are given as means ± sd, values for relaxivities are mean ± standard error of the regression.

	MNP@PMAO-PtCx1			MNP@OH-PtCx2			Resovist®		
[Fe] / µM	T_1 /ms	T_2 / ms	T_2* / ms	T_1 / ms	T_2 / ms	T_2* / ms	T_1 / ms	T_2 / ms	T_2* / ms
90	2330 ± 30	22.04 ± 0.14	16.7 ± 0.6	2360 ± 19	48 ± 5	17.0 ± 1.4	2135 ± 9	41.3 ± 0.4	28 ± 3
180	2240 ± 140	12.7 ± 1.6	10.8 ± 1.5	2390 ± 30	30 ± 5	8.5 ± 1.8	1800 ± 9	22.8 ± 0.3	18.1 ± 1.1
400	2180 ± 170	5.9 ± 0.7	4.8 ± 0.6	2300 ± 60	21.2 ± 1.2	3.3 ± 0.7	1280 ± 30	10.9 ± 0.3	9.9 ± 0.4
Relaxivities									
r_1 / L mmol^{-1} s^{-1}	0.11 ± 0.08			0.04 ± 0.02			1.01 ± 0.02		
r_2 / L mmol^{-1} s^{-1}	500 ± 40			76 ± 14			218 ± 3		
r_2* / L mmol^{-1} s^{-1}	610 ± 50			795 ± 87			213 ± 7		

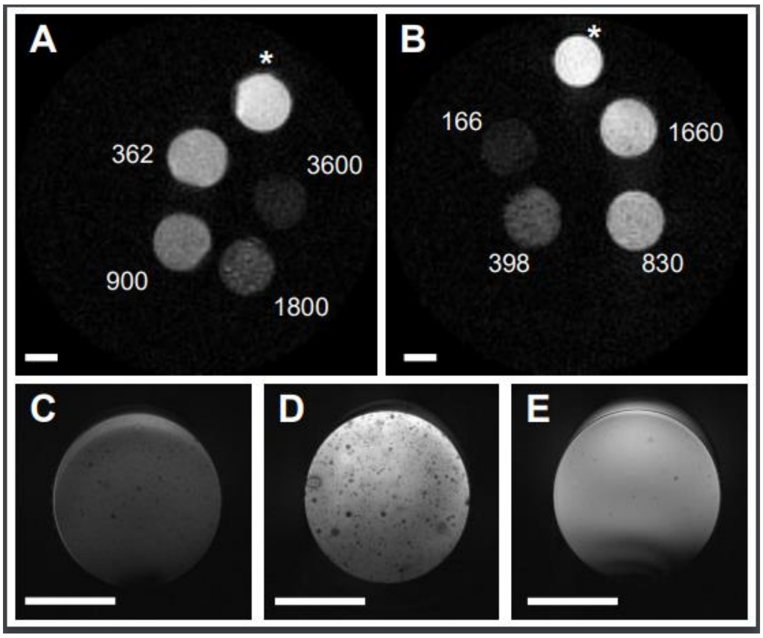

Fig S5. Magnetic resonance images. T_2*-weighted MRI of different concentrations of MNP@PMAO-PtCx1 (A) and MNP@OH-PtCx2 (B). Difference in UTE sequence parameters from text: T_E = 1.35 ms, T_R = 80 ms, 128×128 matrix. Numbers next to the tube cross-sections refer to iron concentrations in µM. The ensembles contain one pure water sample as a reference (*). High-resolution T_2*-weighted images of IONP, acquired with a cryogenic surface coil ([Fe] = 180 µM, see tab. 1, T_E = 15 ms) for MNP@PMAO-PtCx1 (C) and MNP@OH-PtCx2 (D) and pure 1% agarose solution as reference (E). Scale bars = 50 mm.

Section 7: NMR and mass spectra of PtCx1, PtCx2 and ligand precursors

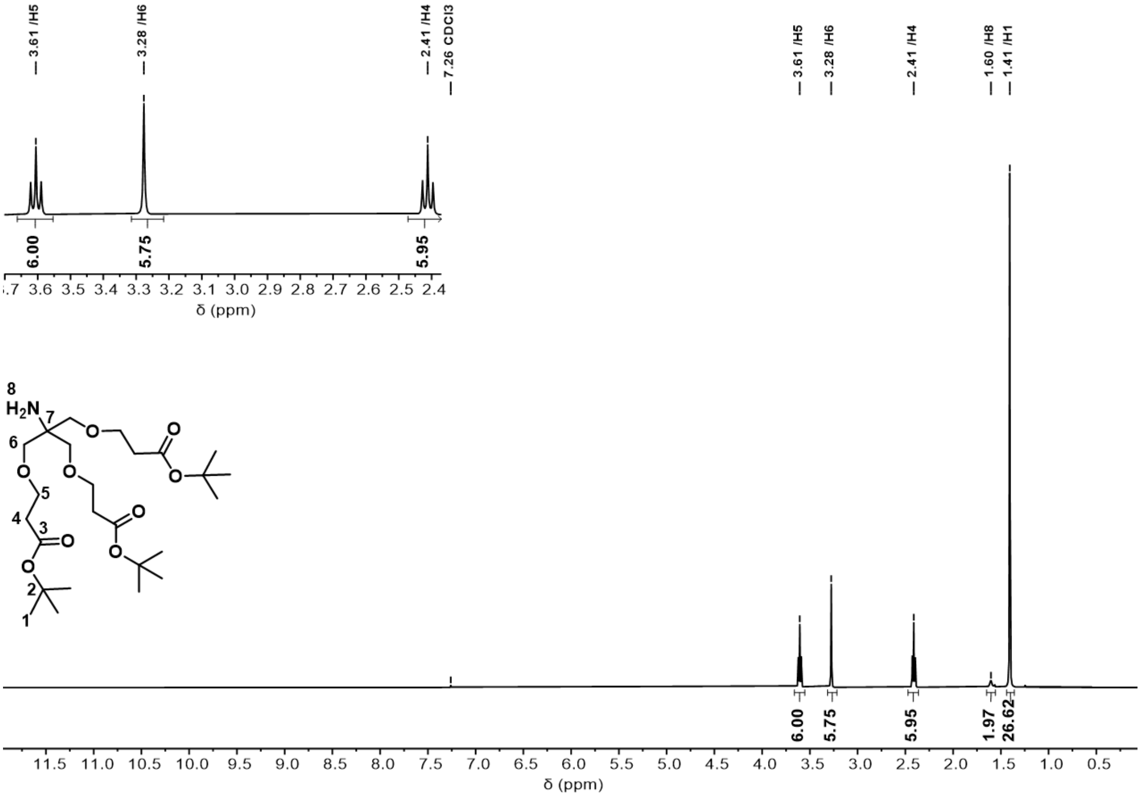

Fig S6. ^{1}H-NMR spectrum (400 MHz, $CDCl_3$) of **1**.

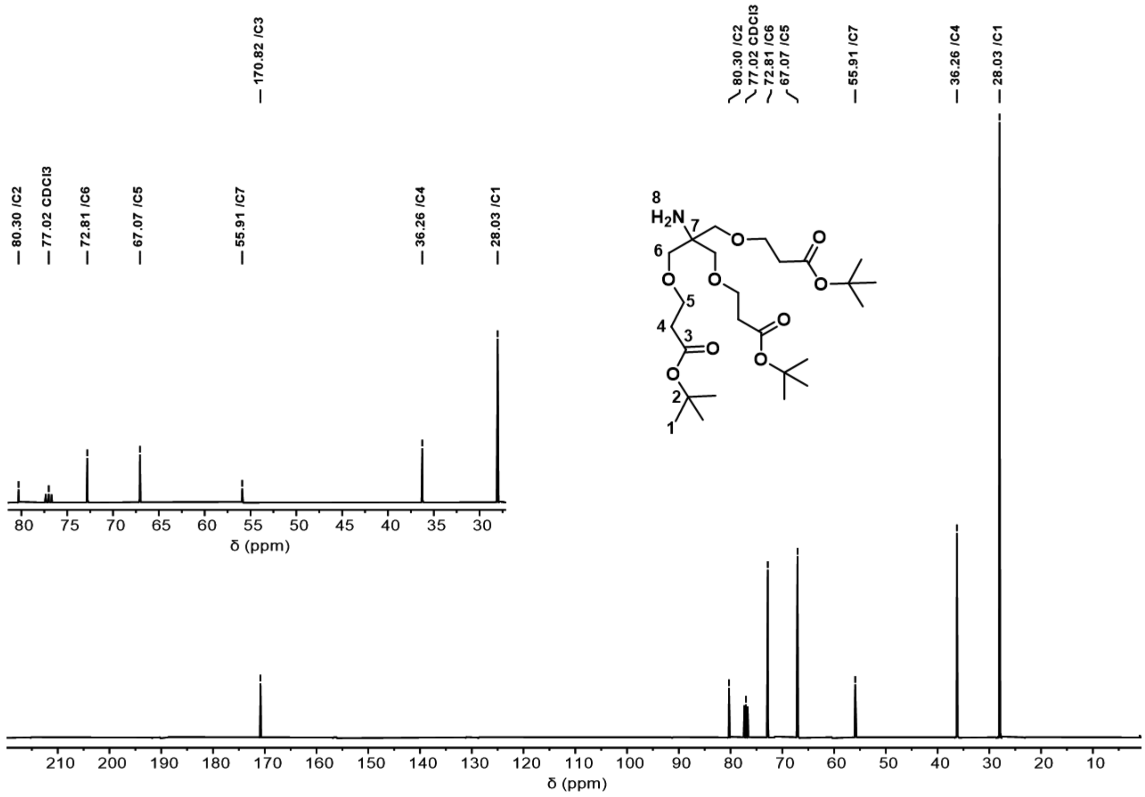

Fig S7. ^{13}C-NMR spectrum (101 MHz, $CDCl_3$) of **1**.

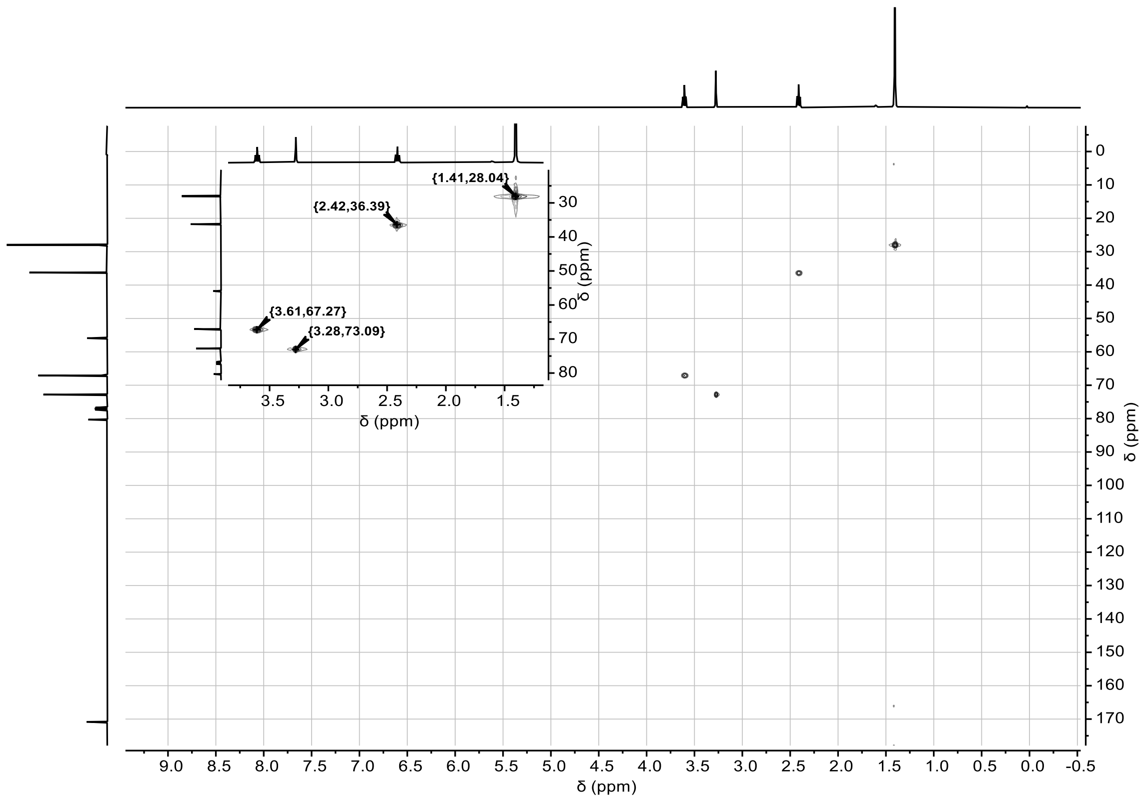

Fig S8. ^{1}H,^{13}C-HSQC-NMR spectrum (400 MHz, 101 MHz, $CDCl_3$) of **1**.

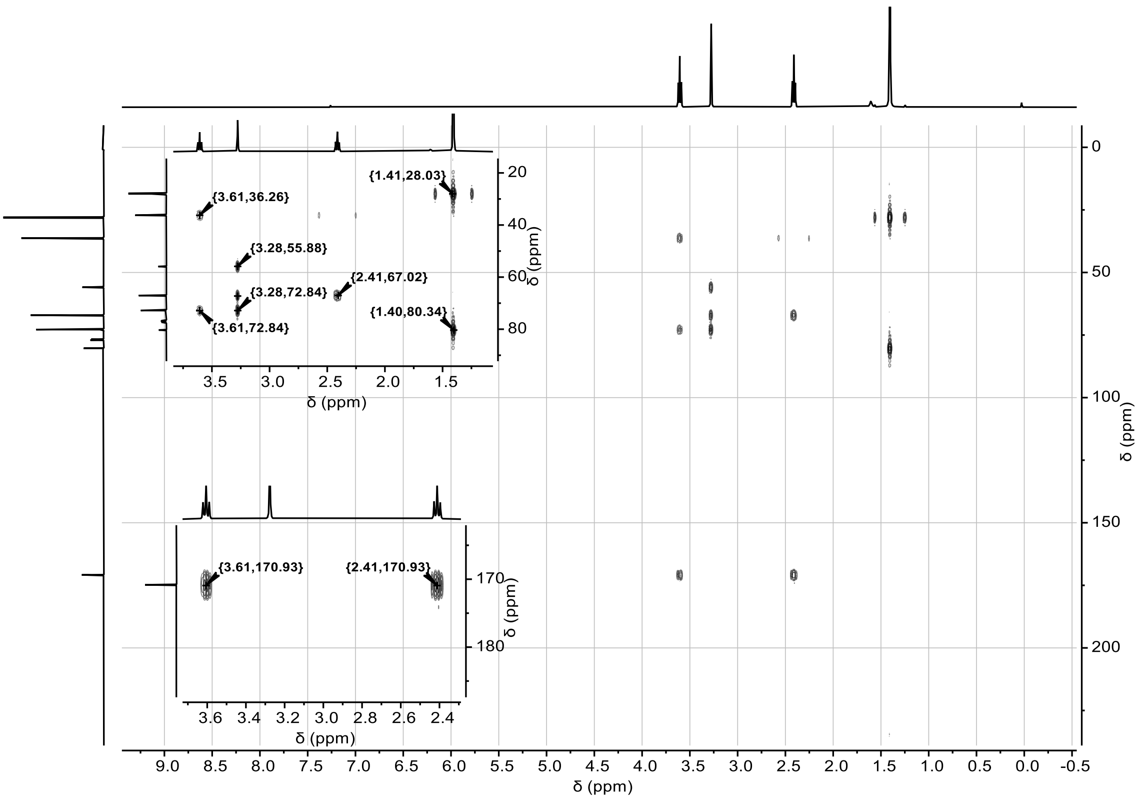

Fig S9. ¹H,¹³C-HMBC-NMR spectrum (400 MHz, 101 MHz*,* $CDCl_3$) of **1**.

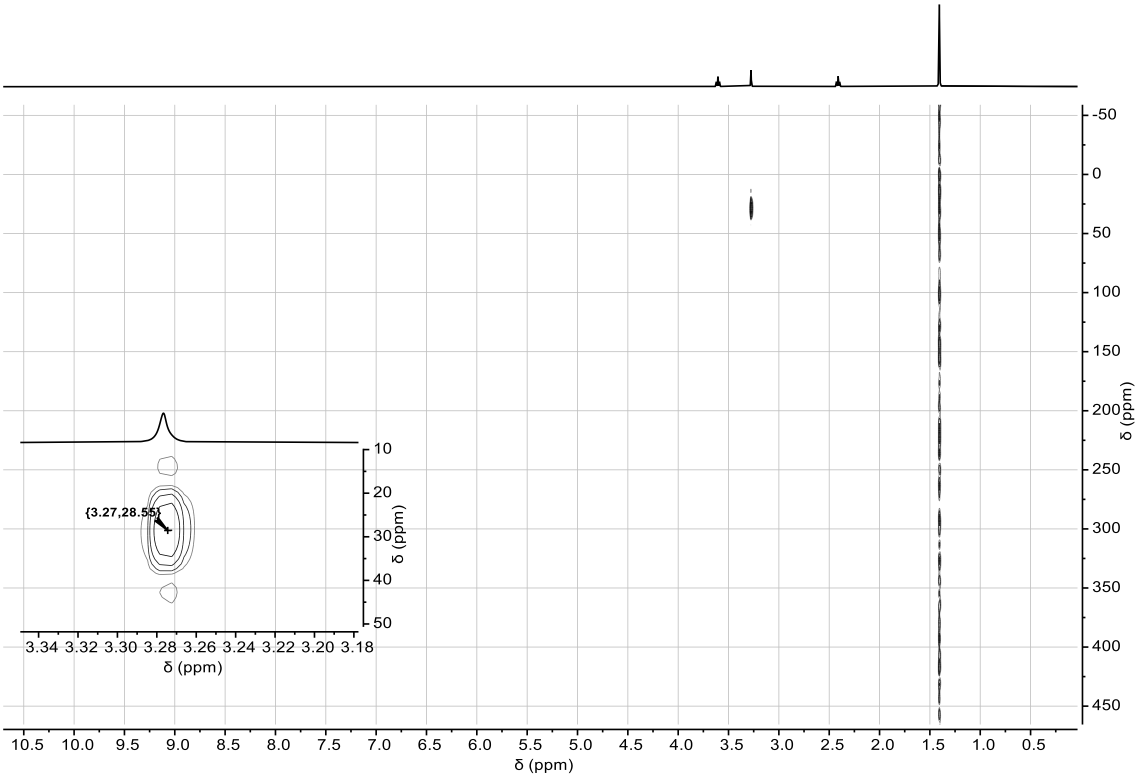

Fig S10. ¹H,¹⁵N-HMBC-NMR spectrum (400 MHz, 41 MHz*,* $CDCl_3$) of **1**.

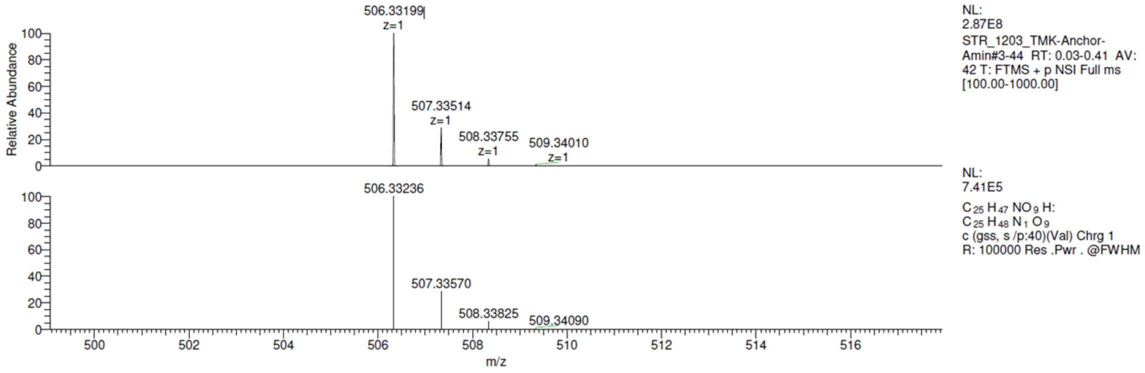

Fig S11. Mass spectrum of **1** (top). Additional simulation of the [**1**+H]$^+$ adduct (bottom).

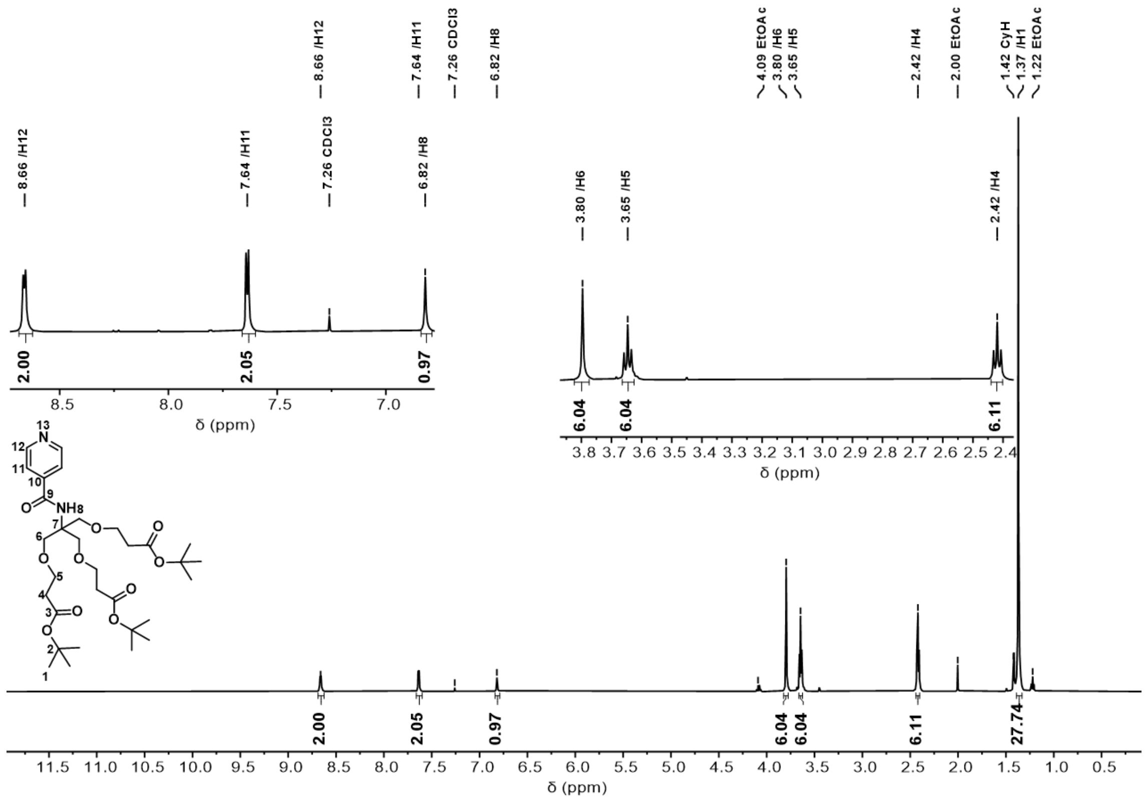

Fig S12. ^{1}H-NMR spectrum (400 MHz, $CDCl_3$) of **2**.

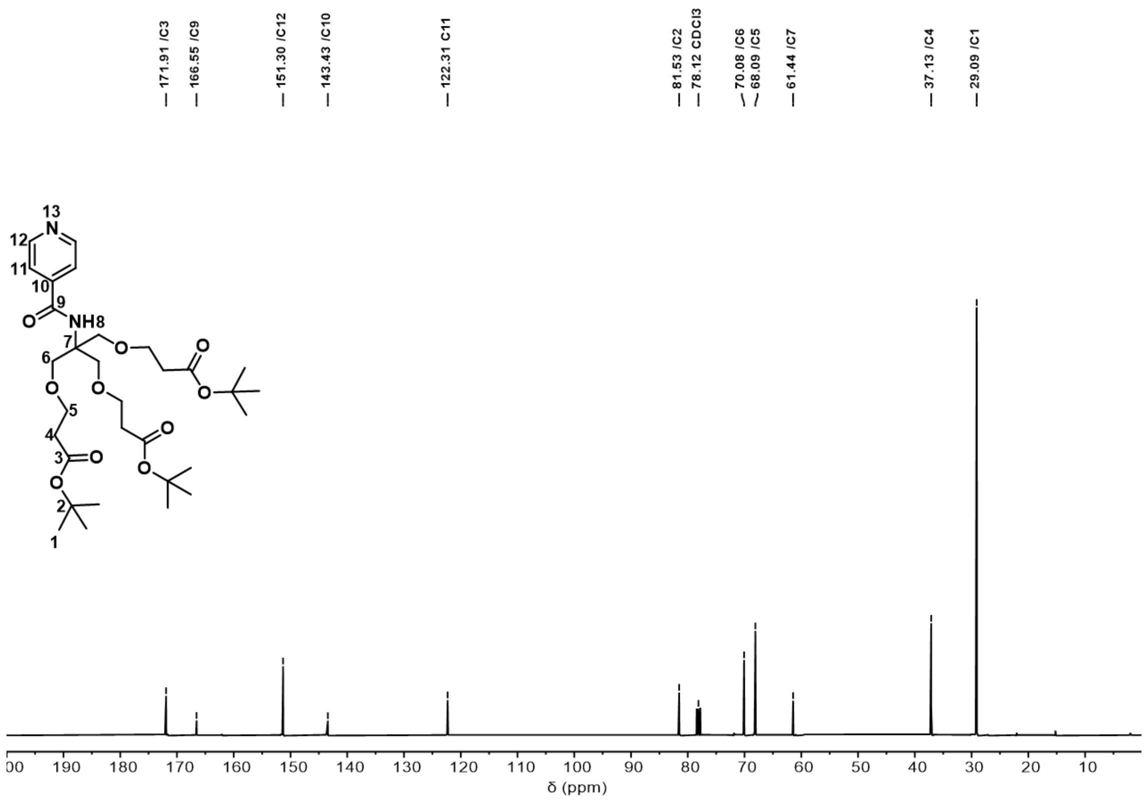

Fig S13. ^{13}C-NMR spectrum (101 MHz, $CDCl_3$) of **2**.

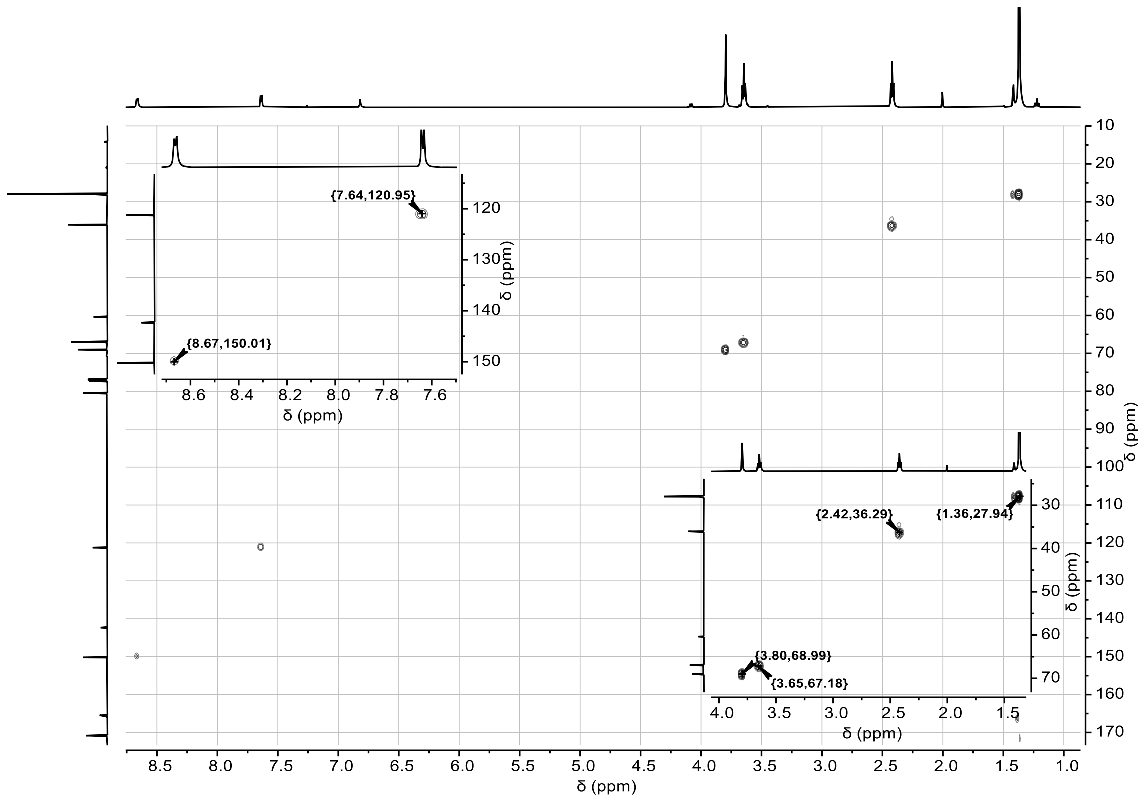

Fig S14. ^{1}H,^{13}C-HSQC-NMR spectrum (400 MHz, 101 MHz, $CDCl_3$) of **2**.

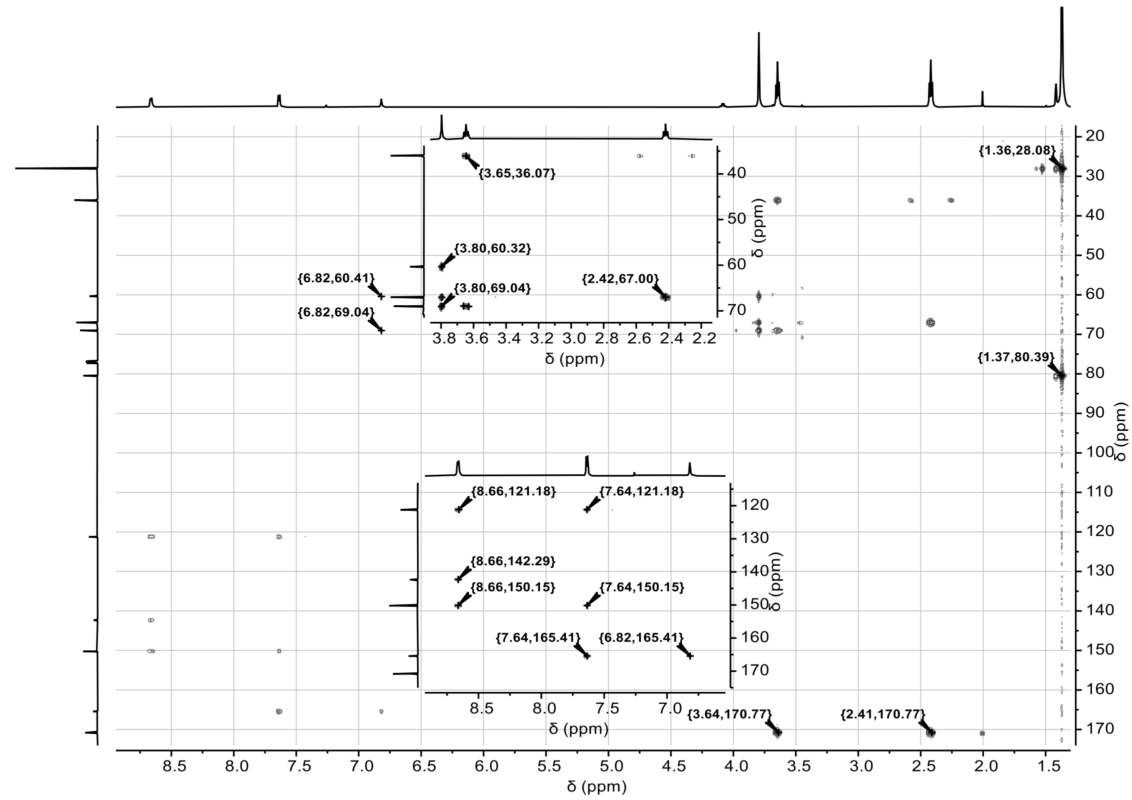

Fig S15. ^{1}H,^{13}C-HMBC-NMR spectrum (400 MHz, 101 MHz, $CDCl_3$) of **2**.

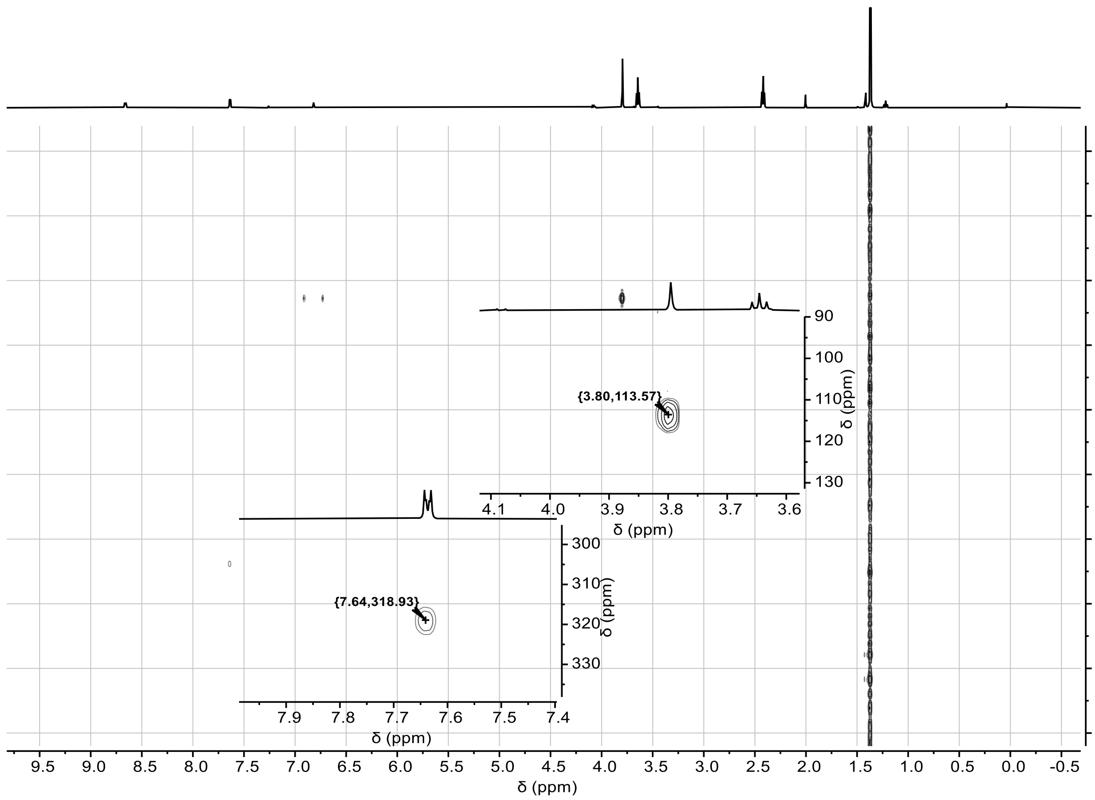

Fig S16. ^{1}H,^{15}N-HMBC-NMR spectrum (500 MHz, 41 MHz, $CDCl_3$) of **2**.

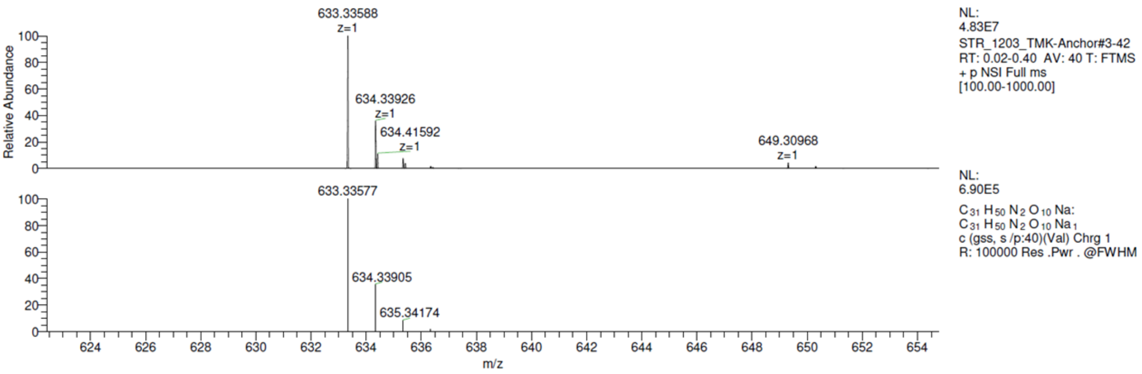

Fig S17. Mass spectrum of **2** (top). Additional simulation of the [**2**+Na]$^+$ adduct (bottom).

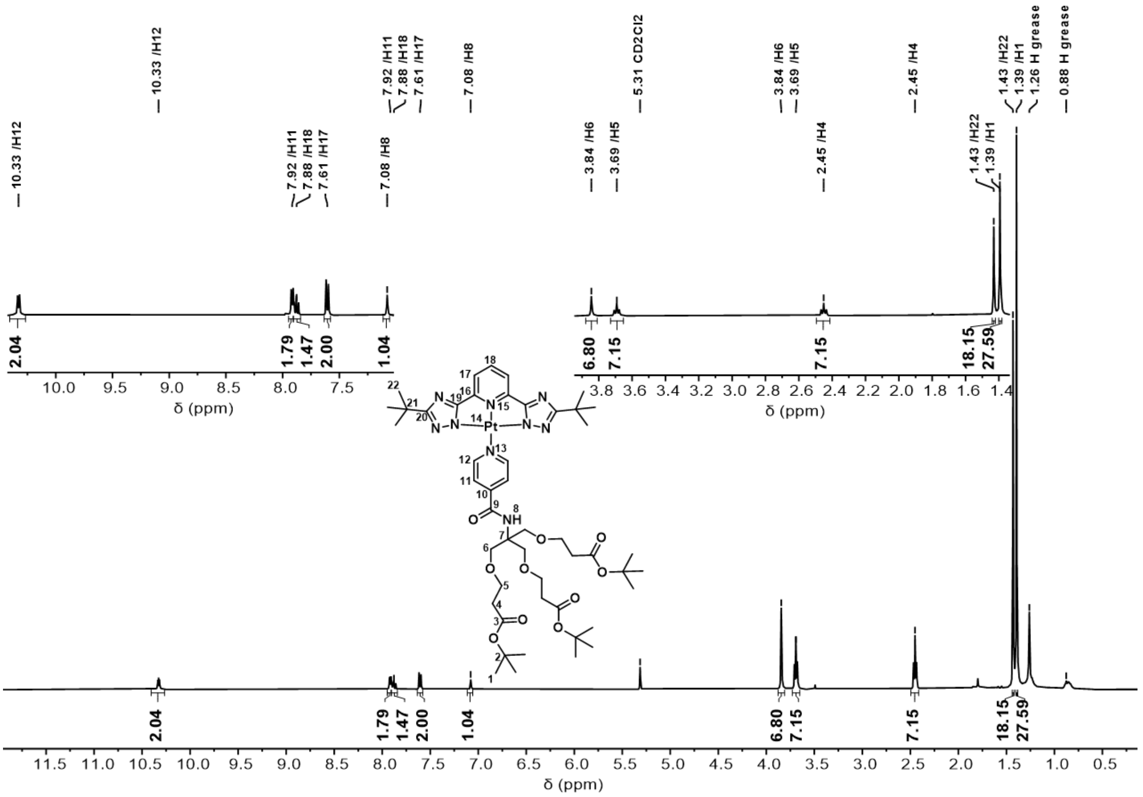

Fig S18. ^{1}H-NMR spectrum (500 MHz, CD_2Cl_2) of **PtCx1**.

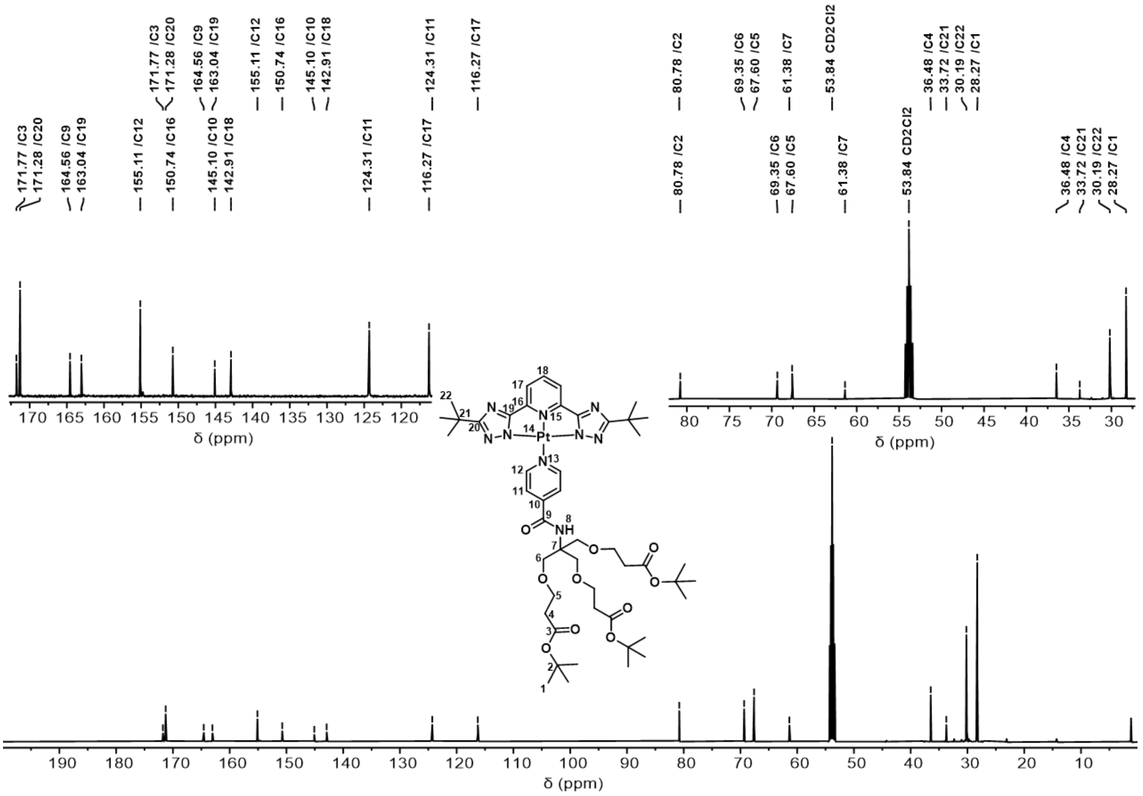

Fig S19. ^{13}C-NMR spectrum (126 MHz, CD_2Cl_2) of **PtCx1**.

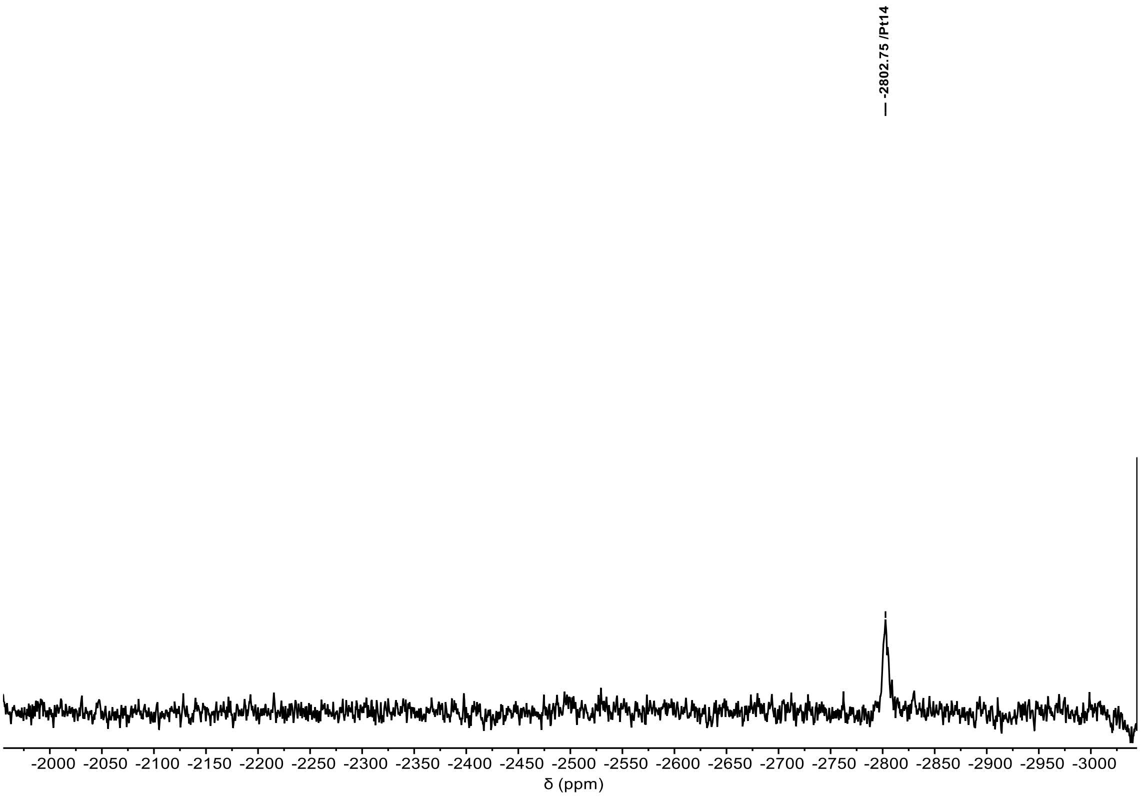

Fig S20. ^{195}Pt-NMR spectrum (86 MHz, CD_2Cl_2) of **PtCx1**.

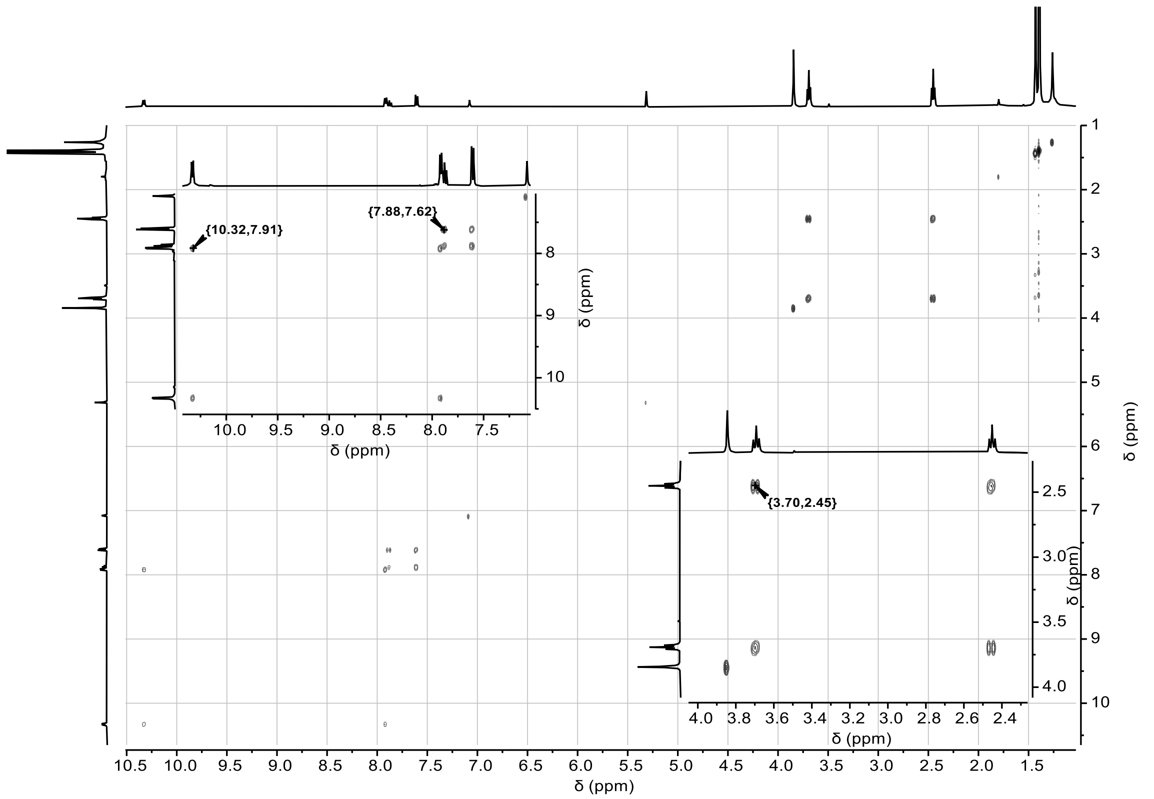

Fig S21. $^{1}H,^{1}H$-COSY-NMR spectrum (500 MHz, CD_2Cl_2) of **PtCx1**.

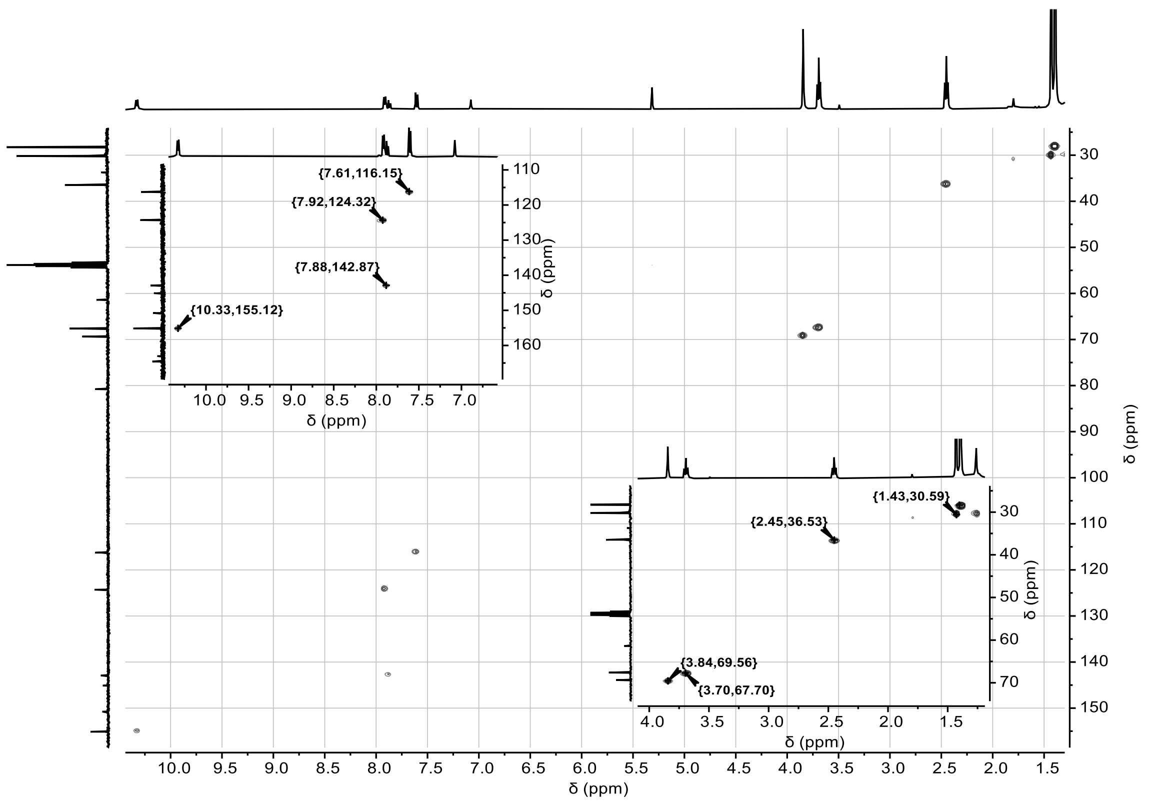

Fig S22. $^{1}H,^{13}C$-HSQC-NMR spectrum (500 MHz, 126 MHz, CD_2Cl_2) of **PtCx1**.

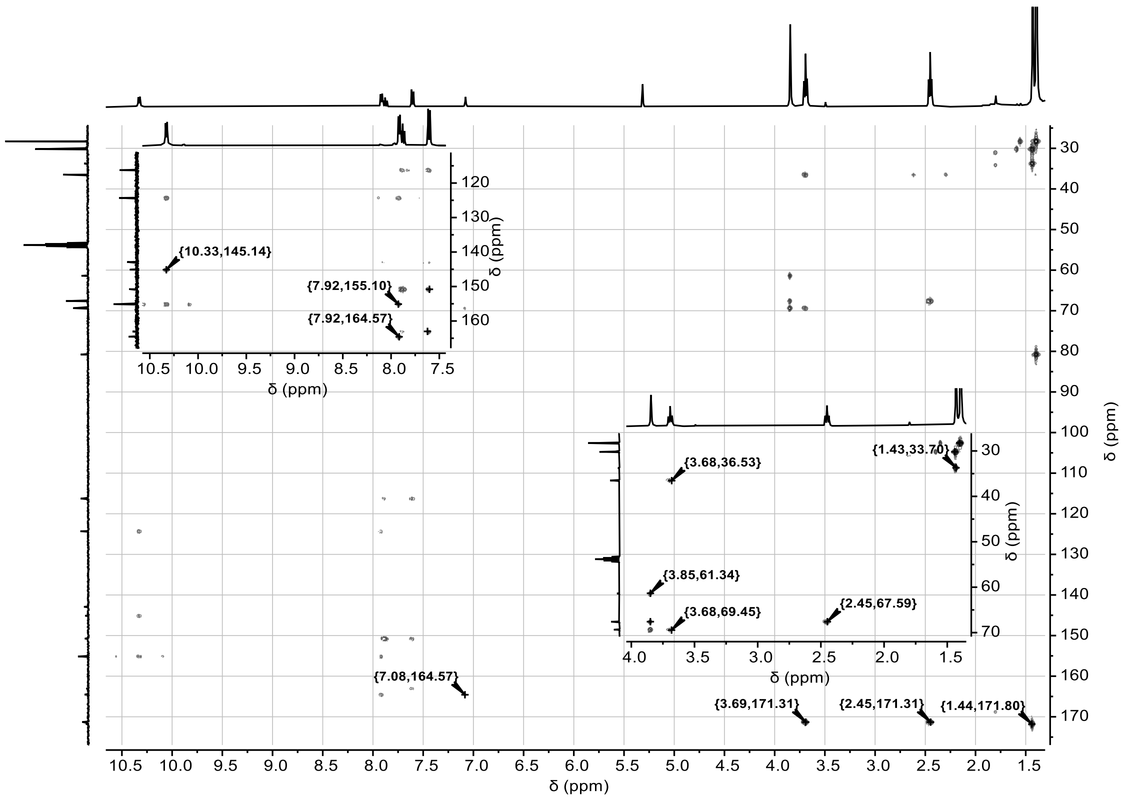

Fig S23. $^{1}H,^{13}C$-HMBC-NMR spectrum (500 MHz, 126 MHz, CD_2Cl_2) of **PtCx1**.

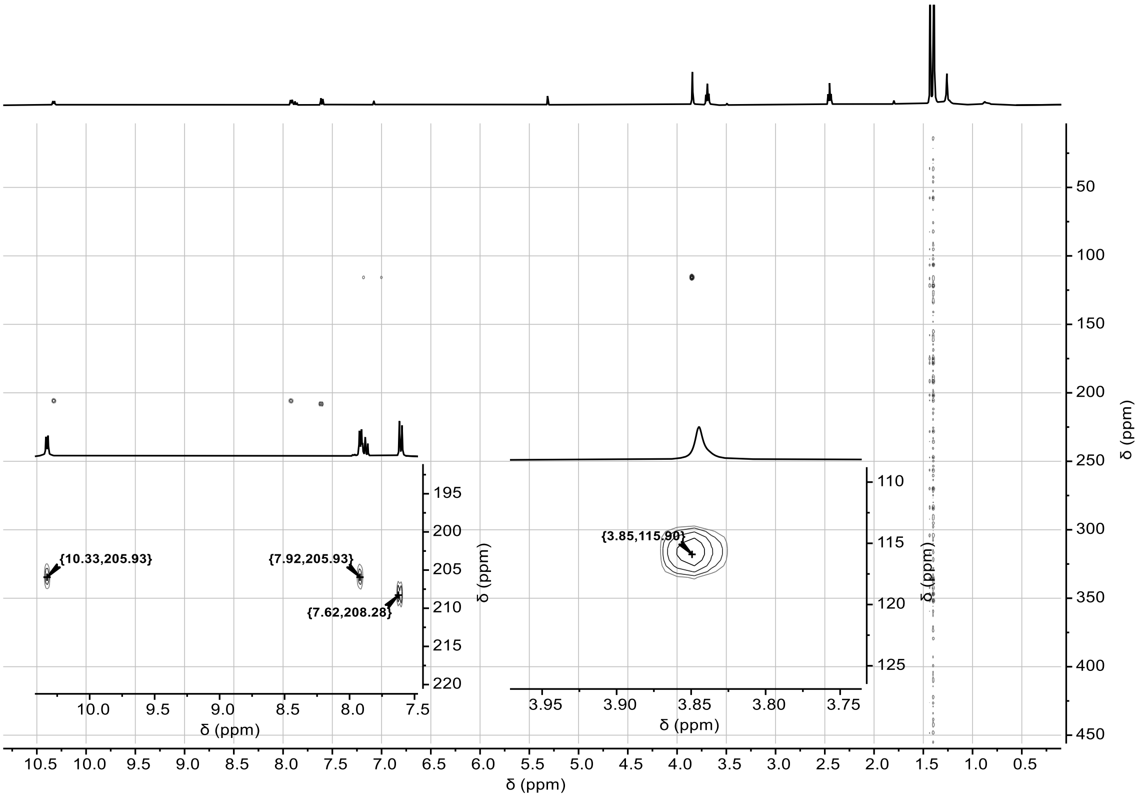

Fig S24. $^{1}H,^{15}N$-HMBC-NMR spectrum (500 MHz, 51 MHz, CD_2Cl_2) of **PtCx1**.

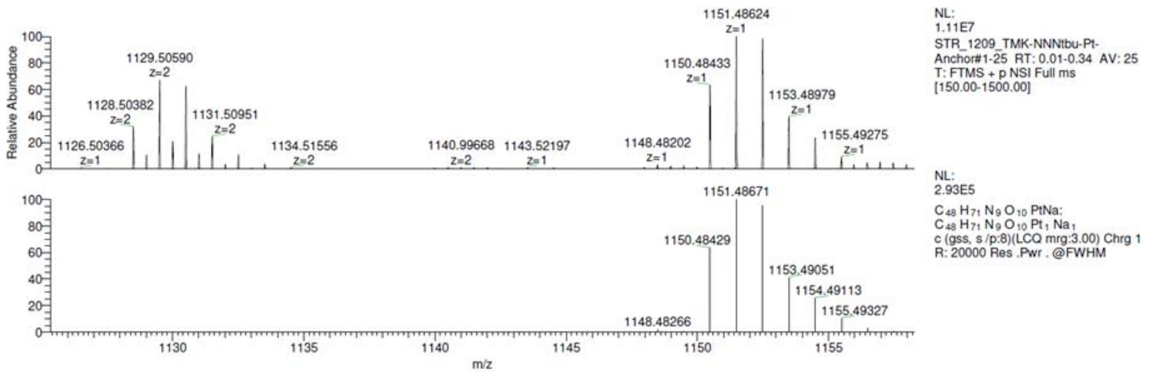

Fig S25. Mass spectrum of **PtCx1** (top). Additional simulation of the [**PtCx1**+Na]$^{+}$ adduct (bottom).

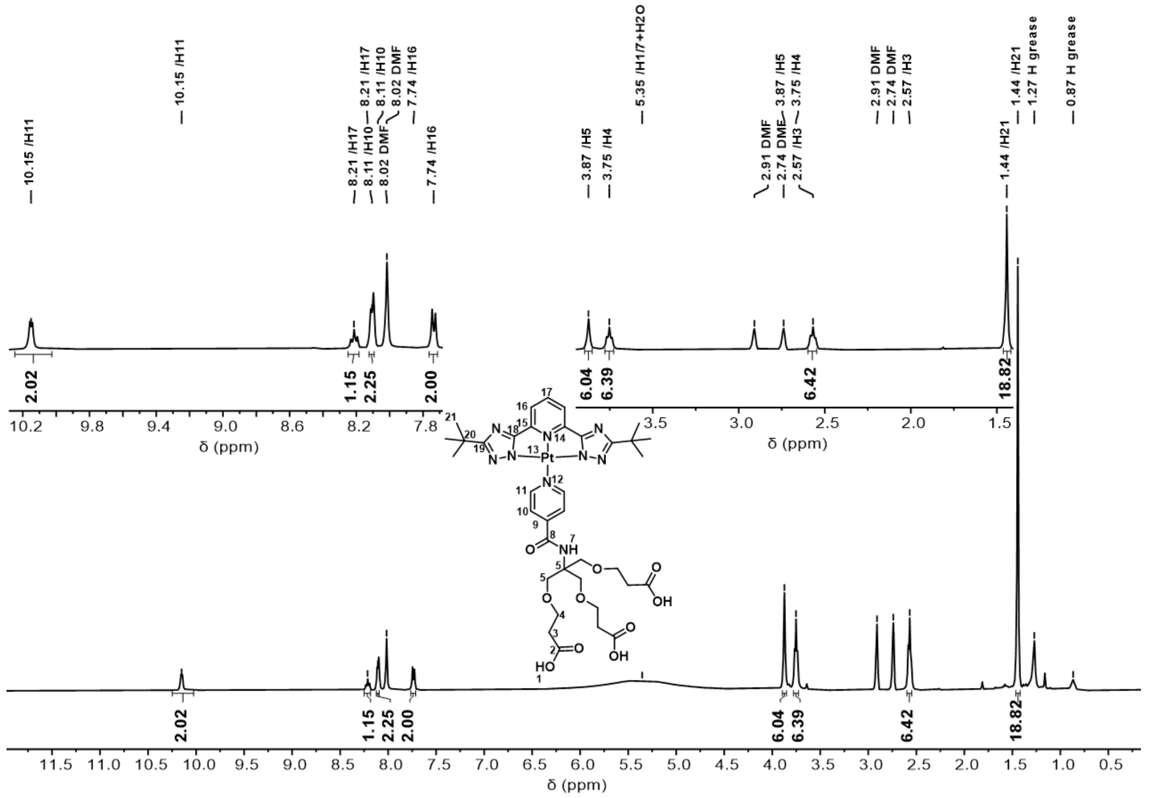

Fig S26. ^{1}H-NMR spectrum (400 MHz, DMF-d_7) of **PtCx2**.

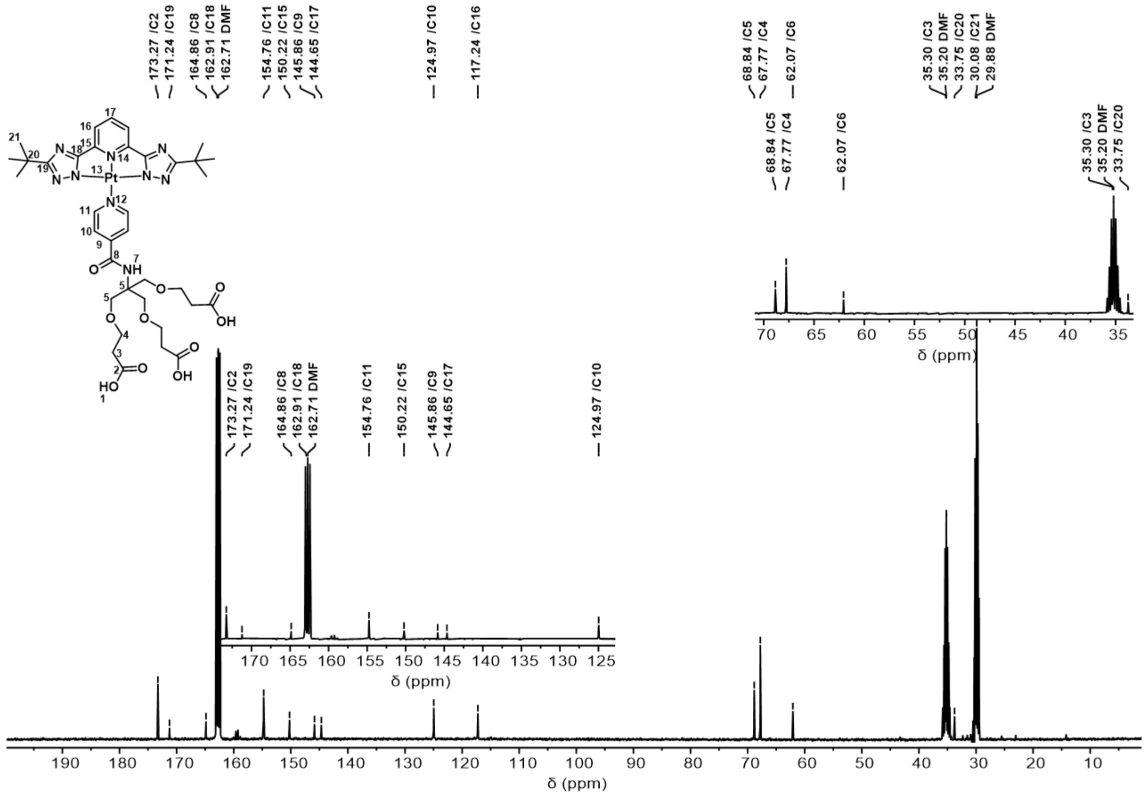

Fig S27. ^{13}C-NMR spectrum (101 MHz, DMF-d_7) of **PtCx2**.

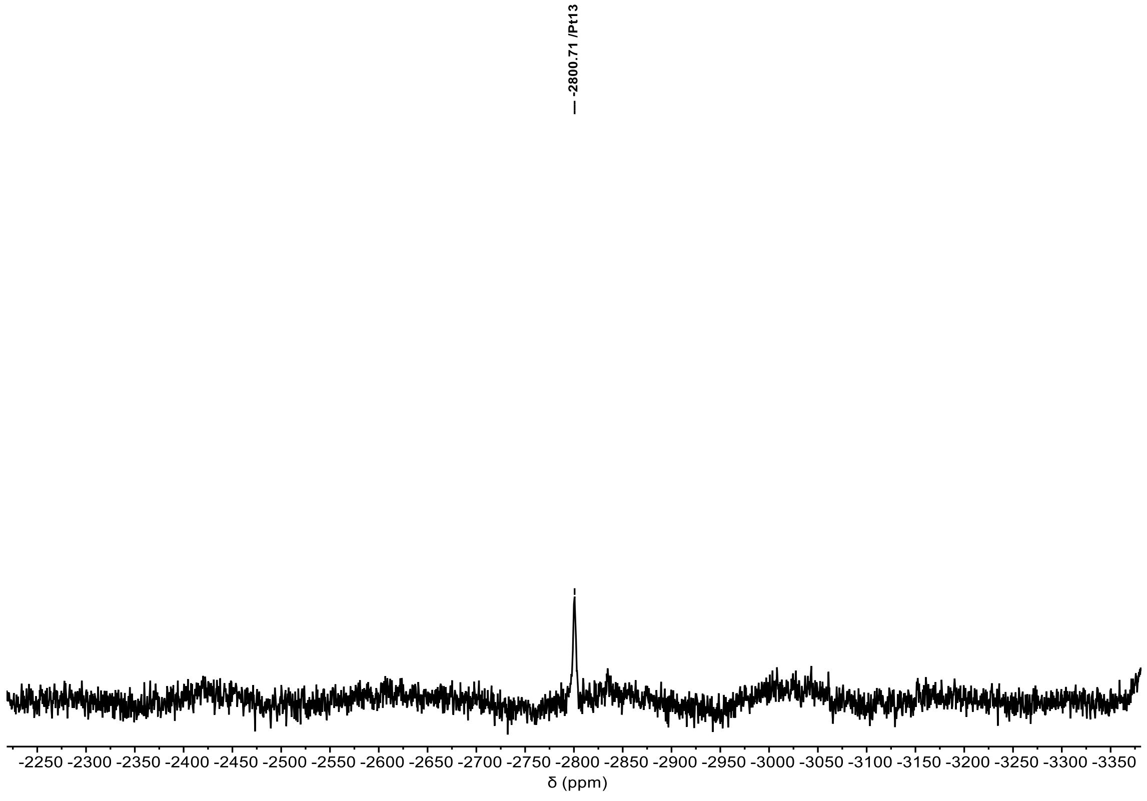

Fig S28. ^{195}Pt-NMR spectrum (86 MHz, DMF-d_7) of **PtCx2**.

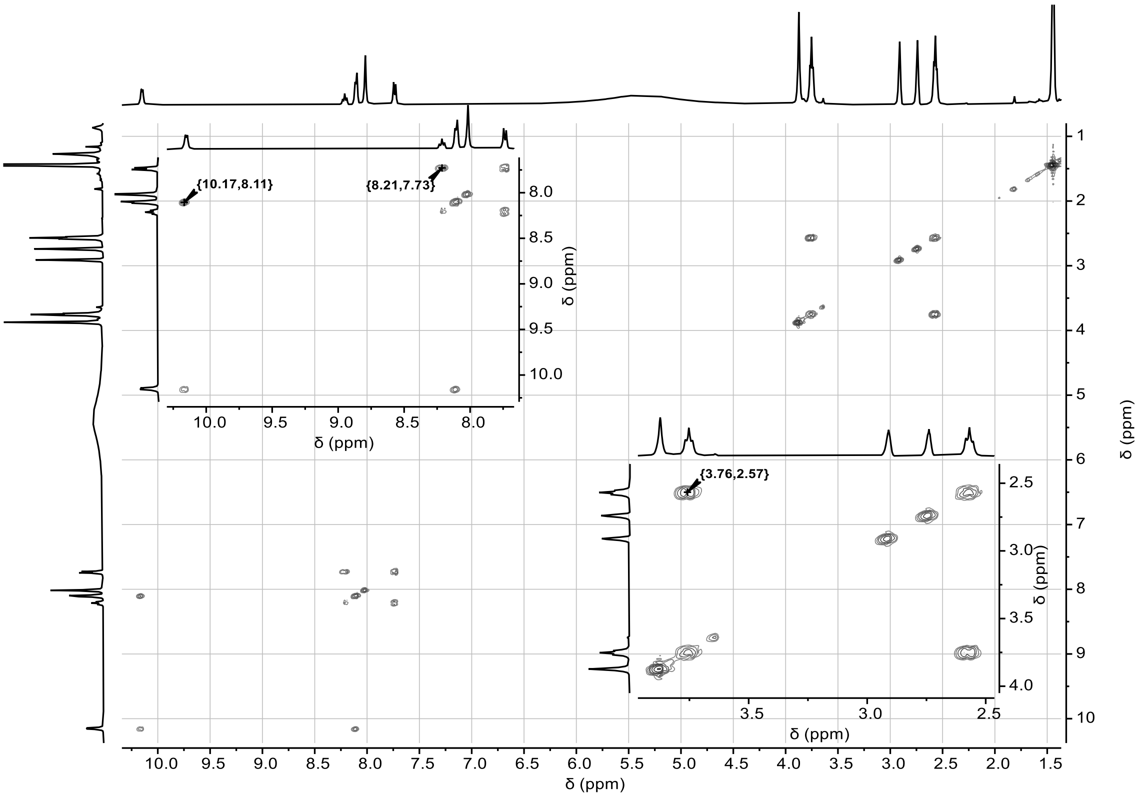

Fig S29. ^{1}H,^{1}H-COSY-NMR spectrum (400 MHz, DMF-d_7) of **PtCx2**.

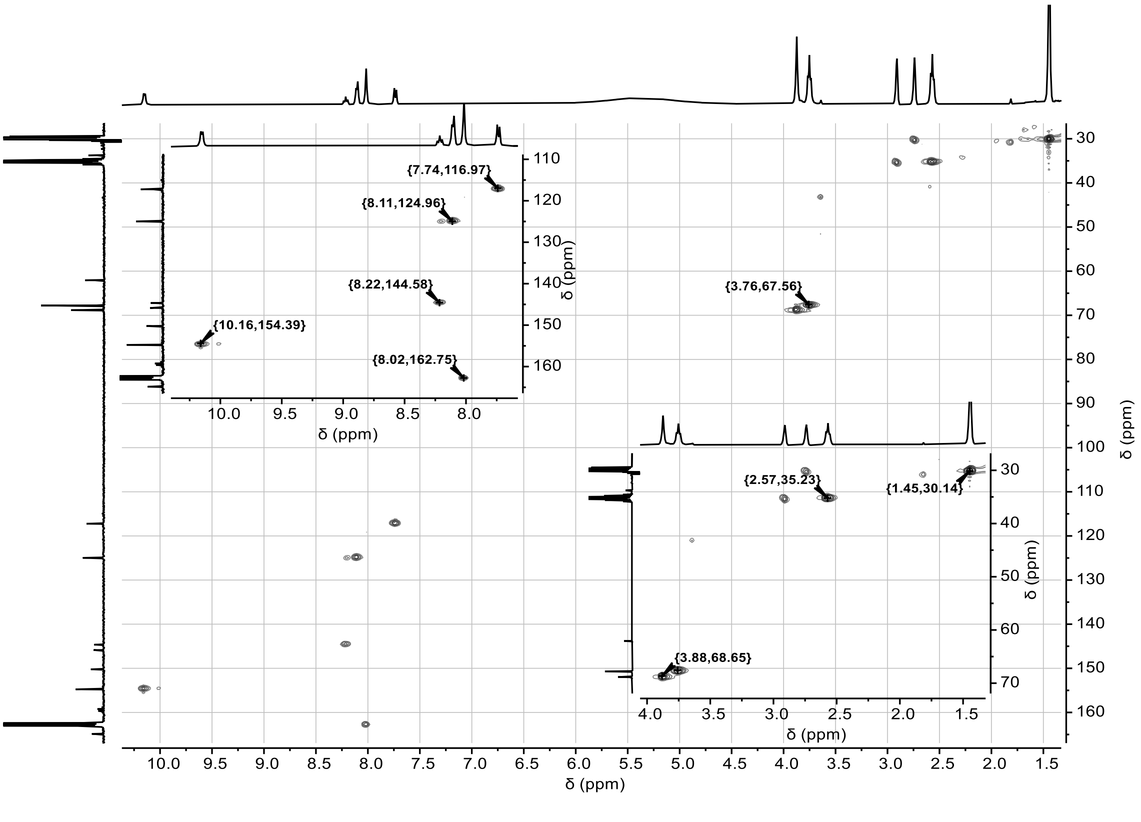

Fig S30. ^{1}H,^{13}C-HSQC-NMR spectrum (400 MHz, 101 MHz, DMF-d_7) of **PtCx2**.

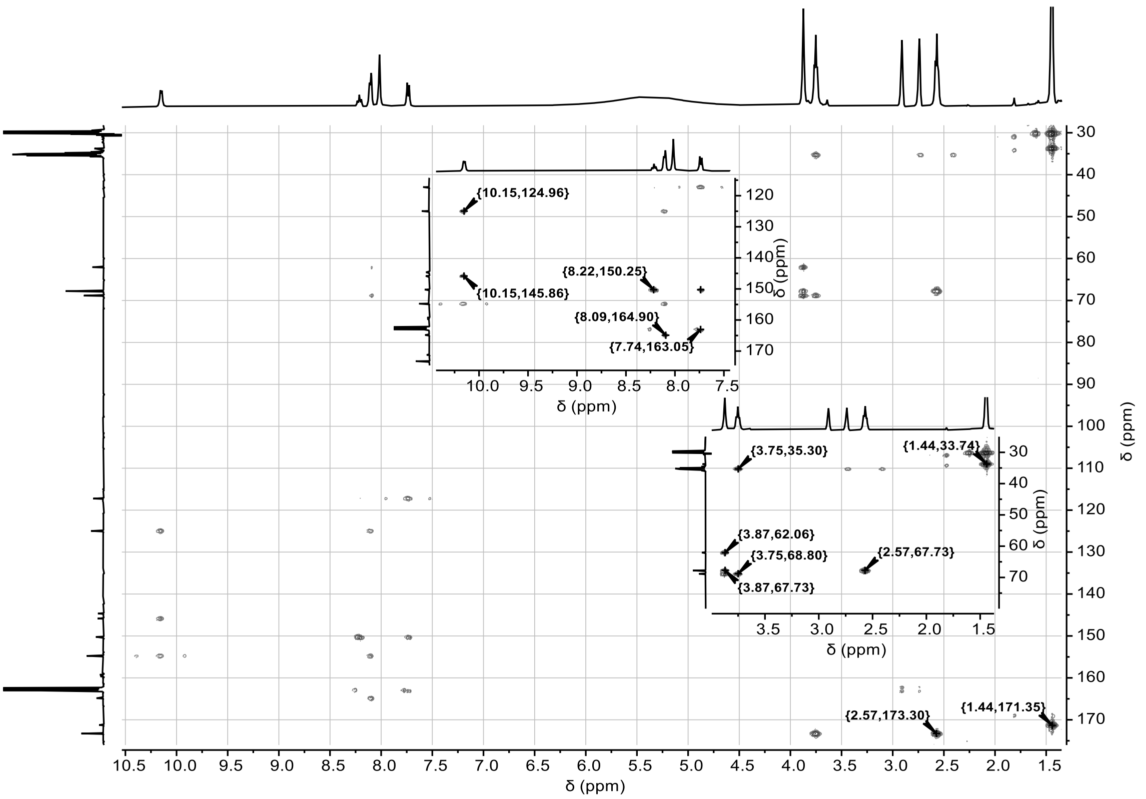

Fig S31. ^{1}H,^{13}C-HMBC-NMR spectrum (400 MHz, 101 MHz, DMF-d_7) of **PtCx2**.

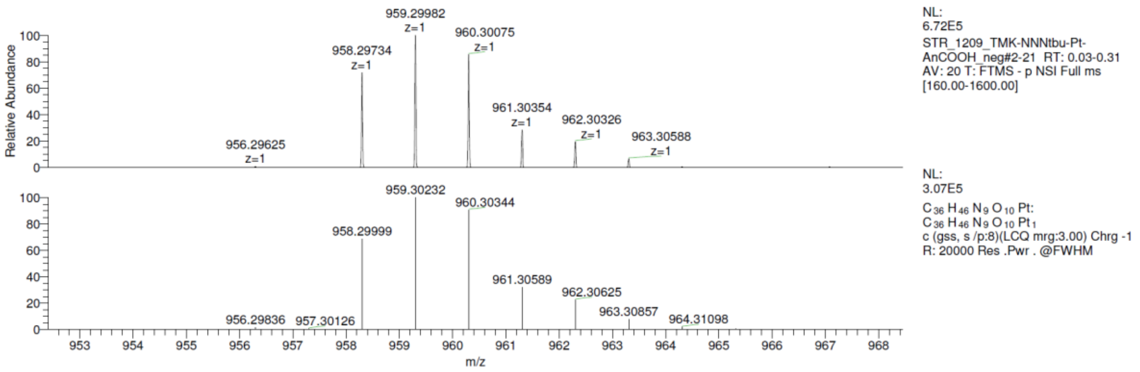

Fig S32. Mass spectrum of **PtCx2** (top). Additional simulation of the [**PtCx2**-H]$^-$ ion (bottom).

Section 7: Time resolved photoluminescence decays and fitting parameters

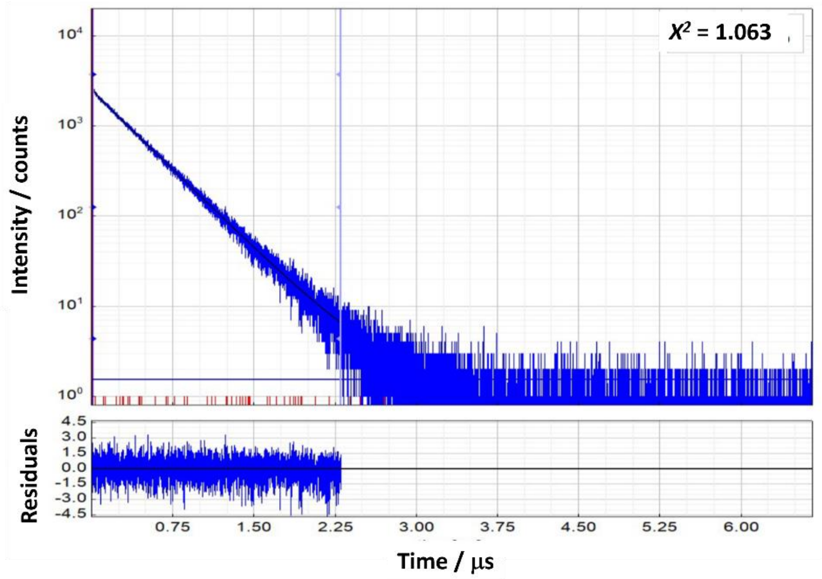

Parameter	Value	Error
A_1[kCnts/Chnl]	2.446	± 0.014
τ_1[ns]	371.71	± 0.66
I_1[kCnts]	1776	± 11
$A_{Rel\,1}$[%]	36.3	± 3.2
$I_{Rel\,1}$[%]	97.627	± 0.091
A_2[kCnts/Chnl]	0.46	± 0.12
τ_2[ns]	17.5	± 2.5
I_2[kCnts]	15.6	± 1.4
$A_{Rel\,2}$[%]	6.7	± 1.5
$I_{Rel\,2}$[%]	0.855	± 0.072
A_3[kCnts/Chnl]	3.85	± 0.59
τ_3[ns]	3.68	± 0.40
I_3[kCnts]	27.6	± 2.3
$A_{Rel\,3}$[%]	57.0	± 3.8
$I_{Rel\,3}$[%]	1.52	± 0.14
Bkgr$_{Dec}$[kCnts]	0.0015	± 0.0002
Bkgr$_{IRF}$[Cnts/Chnl]	-0.2	± 2.3
Shift$_{IRF}$[ps]	0.2	± 2.0
$\tau_{Av\,Int}$[ns]	363.09	± 0.87
$\tau_{Av\,Amp}$[ns]	138	± 13

Fig S33. Left: Raw (experimental) time-resolved photoluminescence decay of **PtCx1** in fluid air-equilibrated DCM solution at 298 K ($c = 10^{-5}$ M) and the instrument response function (red), including the residuals ($\lambda_{ex} = 376.7$ nm, $\lambda_{em} = 550$ nm). Right: Fitting parameters including pre-exponential factors and confidence limits.

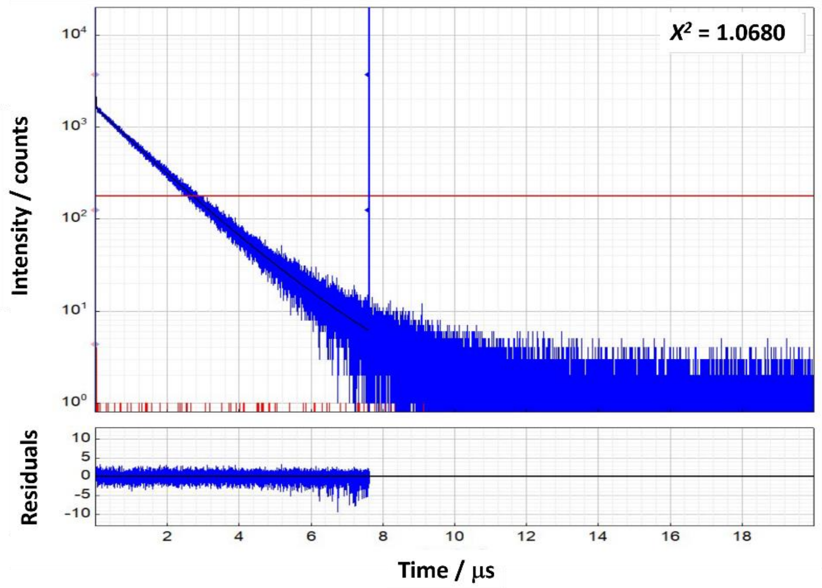

Parameter	Value	Error
A_1[kCnts/Chnl]	0.302	± 0.014
τ_1[ns]	1881	± 42
I_1[kCnts]	1111	± 69
$A_{Rel\,1}$[%]	4.27	± 0.42
$I_{Rel\,1}$[%]	20.2	± 1.2
A_2[kCnts/Chnl]	1.976	± 0.064
τ_2[ns]	1121.3	± 8.1
I_2[kCnts]	4328	± 152
$A_{Rel\,2}$[%]	27.9	± 2.6
$I_{Rel\,2}$[%]	78.7	± 1.3
A_3[kCnts/Chnl]	4.80	± 0.62
τ_3[ns]	6.42	± 0.75
I_3[kCnts]	60.2	± 3.3
$A_{Rel\,3}$[%]	67.8	± 2.9
$I_{Rel\,3}$[%]	1.095	± 0.042
Bkgr$_{Dec}$[kCnts]	0.0008	± 0.0005
Bkgr$_{IRF}$[Cnts/Chnl]	178	± 17
Shift$_{IRF}$[ps]	4.7	± 3.9
$\tau_{Av\,Int}$[ns]	1262.6	± 6.6
$\tau_{Av\,Amp}$[ns]	398	± 35

Fig S34. Left: Time-resolved photoluminescence decay of **PtCx1** in fluid argon-purged DCM solution at 298 K ($c = 10^{-5}$ M) and the instrument response function (red), including the residuals ($\lambda_{ex} = 376.7$ nm, $\lambda_{em} = 550$ nm). Right: Fitting parameters including pre-exponential factors and confidence limits.

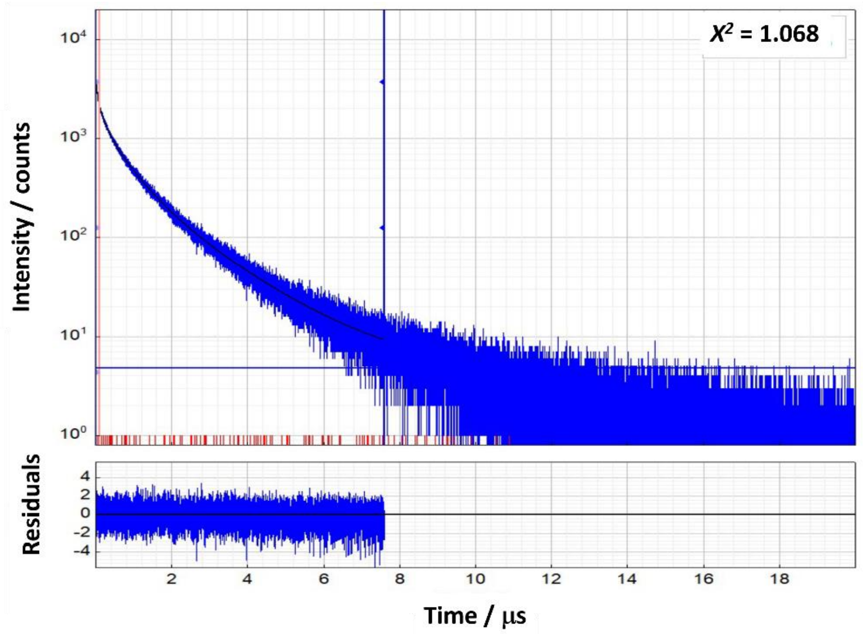

Parameter	Value	Error
A_1[kCnts/Chnl]	0.370	± 0.023
τ_1[ns]	1664	± 47
I_1[kCnts]	1202	± 38
$A_{Rel\,1}$[%]	10.17	± 0.56
$I_{Rel\,1}$[%]	43.1	± 2.2
A_2[kCnts/Chnl]	0.958	± 0.021
τ_2[ns]	622	± 26
I_2[kCnts]	1164	± 49
$A_{Rel\,2}$[%]	26.35	± 0.52
$I_{Rel\,2}$[%]	41.8	± 1.1
A_3[kCnts/Chnl]	0.960	± 0.030
τ_3[ns]	181	± 11
I_3[kCnts]	339	± 25
$A_{Rel\,3}$[%]	26.38	± 0.91
$I_{Rel\,3}$[%]	12.16	± 0.62
A_4[kCnts/Chnl]	1.350	± 0.050
τ_4[ns]	30.82	± 0.70
I_4[kCnts]	81.2	± 4.3
$A_{Rel\,4}$[%]	37.1	± 1.4
$I_{Rel\,4}$[%]	2.92	± 0.18
$Bkgr_{Dec}$[kCnts]	0.0048	± 0.0005
$Bkgr_{IRF}$[Cnts/Chnl]	-0.08	± 0.61
$Shift_{IRF}$[ps]	42.7	± 2.2
$\tau_{Av\,Int}$[ns]	1000.7	± 9.4
$\tau_{Av\,Amp}$[ns]	392.2	± 4.6

Fig S35. Left: Time-resolved photoluminescence decay of **PtCx1** in amorphous solid state (powder) at 298 K and the instrument response function (red), including the residuals (λ_{ex} = 376.7 nm, λ_{em} = 550 nm). Right: Fitting parameters including pre-exponential factors and confidence limits

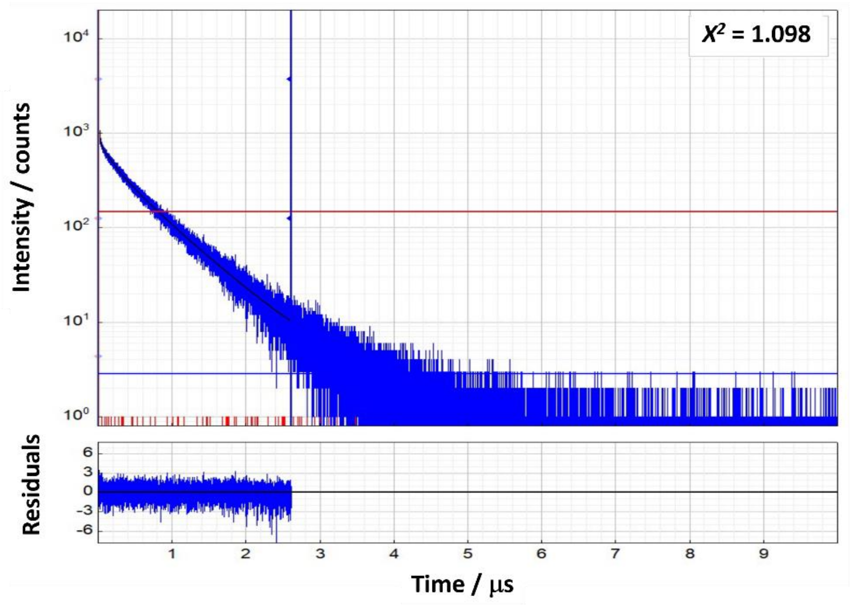

Parameter	Value	Error
A_1[kCnts/Chnl]	0.7436	± 0.0073
τ_1[ns]	611.4	± 3.6
I_1[kCnts]	1776	± 26
$A_{Rel\,1}$[%]	17.09	± 0.86
$I_{Rel\,1}$[%]	83.05	± 0.48
A_2[kCnts/Chnl]	0.3998	± 0.0060
τ_2[ns]	177.2	± 3.8
I_2[kCnts]	277	± 11
$A_{Rel\,2}$[%]	9.19	± 0.48
$I_{Rel\,2}$[%]	12.94	± 0.37
A_3[kCnts/Chnl]	3.21	± 0.24
τ_3[ns]	6.84	± 0.13
I_3[kCnts]	85.7	± 4.2
$A_{Rel\,3}$[%]	73.7	± 1.4
$I_{Rel\,3}$[%]	4.01	± 0.19
$Bkgr_{Dec}$[kCnts]	0.0028	± 0.0003
$Bkgr_{IRF}$[Cnts/Chnl]	147.7	± 2.9
$Shift_{IRF}$[ps]	2280	± 231
$\tau_{Av\,Int}$[ns]	530.9	± 2.0
$\tau_{Av\,Amp}$[ns]	125.8	± 5.6

Fig S36. Left: Time-resolved photoluminescence decay of **PtCx2** in fluid air-equilibrated DCM solution at 298 K (c = 10^{-5} M) and the instrument response function (red), including the residuals (λ_{ex} = 376.7 nm, λ_{em} = 550 nm). Right: Fitting parameters including pre-exponential factors and confidence limits.

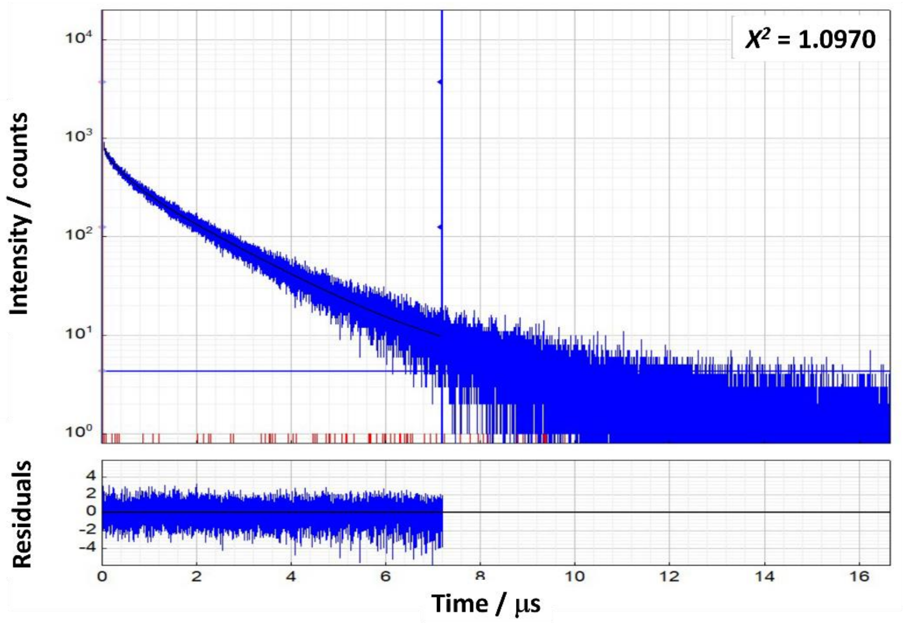

Parameter	Value	Error
A_1[kCnts/Chnl]	3.417	± 0.094
τ_1[ns]	1643	± 19
I_1[kCnts]	10964	± 290
$A_{Rel\,1}$[%]	12.40	± 0.74
$I_{Rel\,1}$[%]	82.58	± 0.80
A_2[kCnts/Chnl]	2.50	± 0.12
τ_2[ns]	408	± 18
I_2[kCnts]	1993	± 130
$A_{Rel\,2}$[%]	9.07	± 0.53
$I_{Rel\,2}$[%]	15.01	± 0.81
A_3[kCnts/Chnl]	1.43	± 0.15
τ_3[ns]	51	± 15
I_3[kCnts]	143	± 33
$A_{Rel\,3}$[%]	5.20	± 0.76
$I_{Rel\,3}$[%]	1.07	± 0.25
A_4[kCnts/Chnl]	20.2	± 1.3
τ_4[ns]	4.49	± 0.24
I_4[kCnts]	177.1	± 2.8
$A_{Rel\,4}$[%]	73.3	± 1.8
$I_{Rel\,4}$[%]	1.334	± 0.062
$Bkgr_{Dec}$[kCnts]	0.0043	± 0.0003
$Bkgr_{IRF}$[Cnts/Chnl]	-3.9	± 1.3
$Shift_{IRF}$[ps]	-39.8	± 1.4
$\tau_{Av\,Int}$[ns]	1418.6	± 9.3
$\tau_{Av\,Amp}$[ns]	247	± 13

Fig S37. Left: Time-resolved photoluminescence decay of **PtCx2** in fluid argon-purged DCM solution at 298 K (c = 10^{-5} M) and the instrument response function (red), including the residuals (λ_{ex} = 376.7 nm, λ_{em} = 550 nm). Right: Fitting parameters including pre-exponential factors and confidence limits.

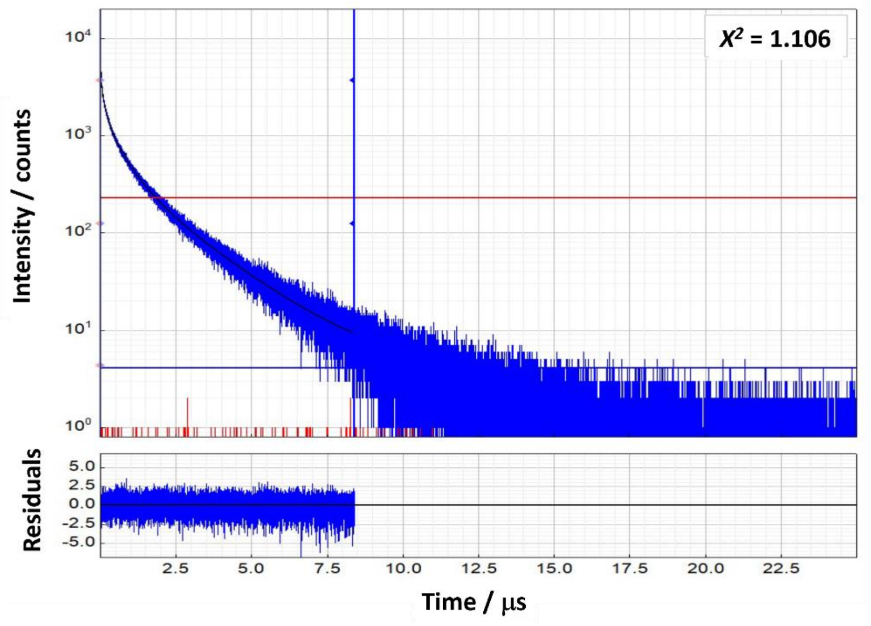

Parameter	Value	Error
A_1[kCnts/Chnl]	0.5587	± 0.0074
τ_1[ns]	1861	± 33
I_1[kCnts]	2031	± 36
$A_{Rel\,1}$[%]	7.78	± 0.16
$I_{Rel\,1}$[%]	47.06	± 0.50
A_2[kCnts/Chnl]	1.351	± 0.014
τ_2[ns]	578	± 14
I_2[kCnts]	1526	± 41
$A_{Rel\,2}$[%]	18.80	± 0.34
$I_{Rel\,2}$[%]	35.35	± 0.35
A_3[kCnts/Chnl]	2.057	± 0.028
τ_3[ns]	151.6	± 6.8
I_3[kCnts]	609	± 29
$A_{Rel\,3}$[%]	28.63	± 0.21
$I_{Rel\,3}$[%]	14.11	± 0.46
A_4[kCnts/Chnl]	3.219	± 0.076
τ_4[ns]	23.8	± 1.4
I_4[kCnts]	150	± 12
$A_{Rel\,4}$[%]	44.80	± 0.33
$I_{Rel\,4}$[%]	3.47	± 0.20
$Bkgr_{Dec}$[kCnts]	0.0041	± 0.0003
$Bkgr_{IRF}$[Cnts/Chnl]	228	± 12
$Shift_{IRF}$[ps]	36.8	± 2.1
$\tau_{Av\,Int}$[ns]	1102.6	± 8.1
$\tau_{Av\,Amp}$[ns]	307.5	± 3.0

Fig S38. Left: Time-resolved photoluminescence decay of **PtCx2** in amorphous solid state (powder) at 298 K and the instrument response function (red), including the residuals (λ_{ex} = 376.7 nm, λ_{em} = 550 nm). Right: Fitting parameters including pre-exponential factors and confidence limits.

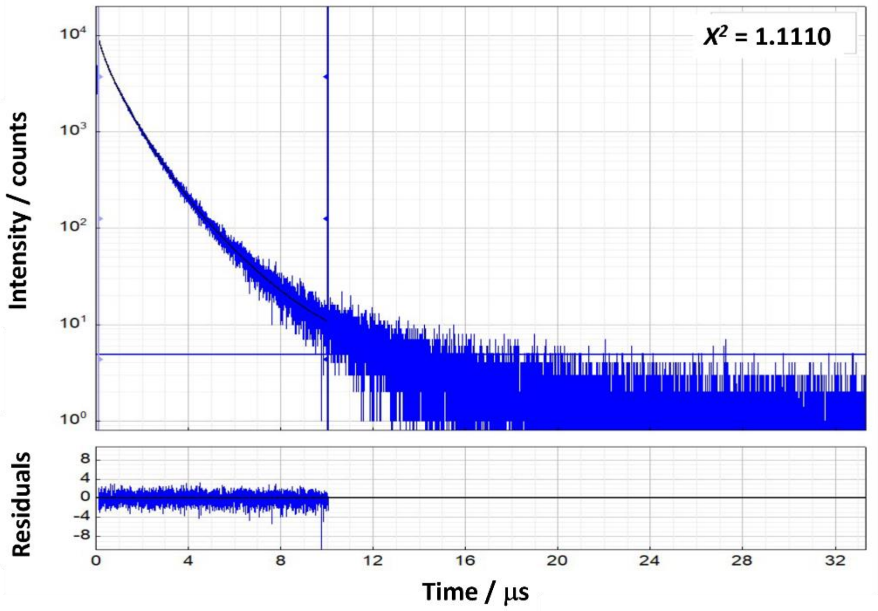

Parameter	Value	Error
A_1[kCnts/Chnl]	1.05	± 0.58
τ_1[ns]	1911	± 477
I_1[kCnts]	999	± 415
$A_{Rel\,1}$[%]	11.3	± 6.2
$I_{Rel\,1}$[%]	28	± 12
A_2[kCnts/Chnl]	5.12	± 0.41
τ_2[ns]	877	± 144
I_2[kCnts]	2246	± 97
$A_{Rel\,2}$[%]	55.4	± 4.4
$I_{Rel\,2}$[%]	62.2	± 2.5
A_3[kCnts/Chnl]	2.26	± 0.52
τ_3[ns]	302	± 126
I_3[kCnts]	342	± 272
$A_{Rel\,3}$[%]	24.5	± 5.9
$I_{Rel\,3}$[%]	9.5	± 7.5
A_4[kCnts/Chnl]	0.81	± 0.39
τ_4[ns]	61	± 29
I_4[kCnts]	25	± 29
$A_{Rel\,4}$[%]	8.8	± 4.2
$I_{Rel\,4}$[%]	0.69	± 0.78
$Bkgr_{Dec}$[kCnts]	0.0049	± 0.0022
$\tau_{Av\,Int}$[ns]	1103	± 24
$\tau_{Av\,Amp}$[ns]	781.6	± 9.0

Fig S39. Left: Time-resolved photoluminescence decay of MNC@PMAO-PtCx1 in air-equilibrated buffer SBB solution at 298 K, including the residuals (λ_{ex}= 376.7 nm, λ_{em} = 550 nm). Right: Fitting parameters including pre-exponential factors and confidence limits.

-

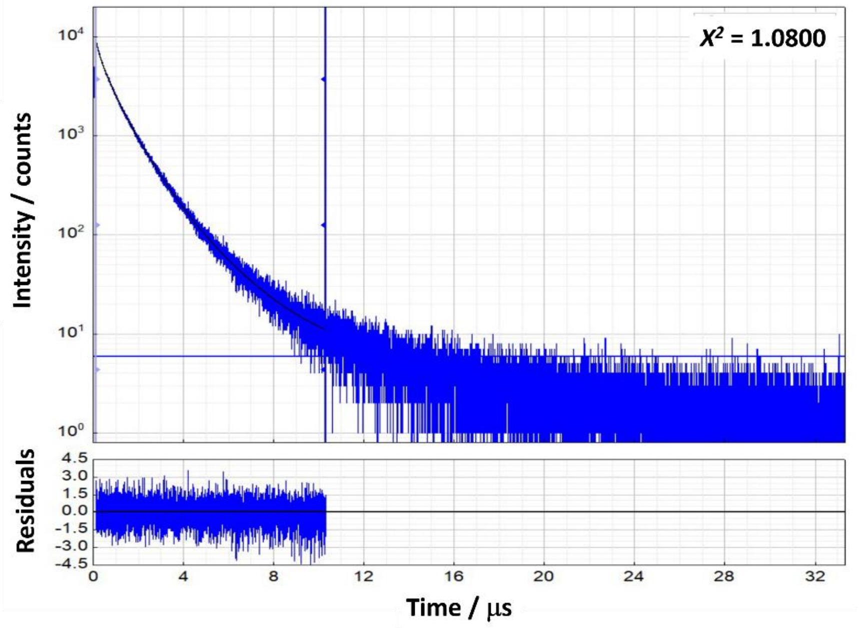

Parameter	Value	Error
A_1[kCnts/Chnl]	0.76	± 0.22
τ_1[ns]	2030	± 243
I_1[kCnts]	770	± 158
$A_{Rel\,1}$[%]	8.4	± 2.5
$I_{Rel\,1}$[%]	22.5	± 4.7
A_2[kCnts/Chnl]	4.78	± 0.33
τ_2[ns]	912	± 58
I_2[kCnts]	2178	± 90
$A_{Rel\,2}$[%]	53.1	± 3.7
$I_{Rel\,2}$[%]	63.6	± 2.5
A_3[kCnts/Chnl]	2.51	± 0.28
τ_3[ns]	350	± 58
I_3[kCnts]	438	± 136
$A_{Rel\,3}$[%]	27.9	± 3.3
$I_{Rel\,3}$[%]	12.8	± 4.0
A_4[kCnts/Chnl]	0.95	± 0.18
τ_4[ns]	76	± 16
I_4[kCnts]	36	± 14
$A_{Rel\,4}$[%]	10.6	± 2.0
$I_{Rel\,4}$[%]	1.05	± 0.41
$Bkgr_{Dec}$[kCnts]	0.0059	± 0.0014
$\tau_{Av\,Int}$[ns]	1083	± 16
$\tau_{Av\,Amp}$[ns]	761.1	± 1.8

Fig S40. Left: Time-resolved photoluminescence decay of MNC@PMAO-PtCx1 in argon-purged buffer SBB solution at 298 K, including the residuals (λ_{ex}= 376.7 nm, λ_{em} = 550 nm). Right: Fitting parameters including pre-exponential factors and confidence limits.

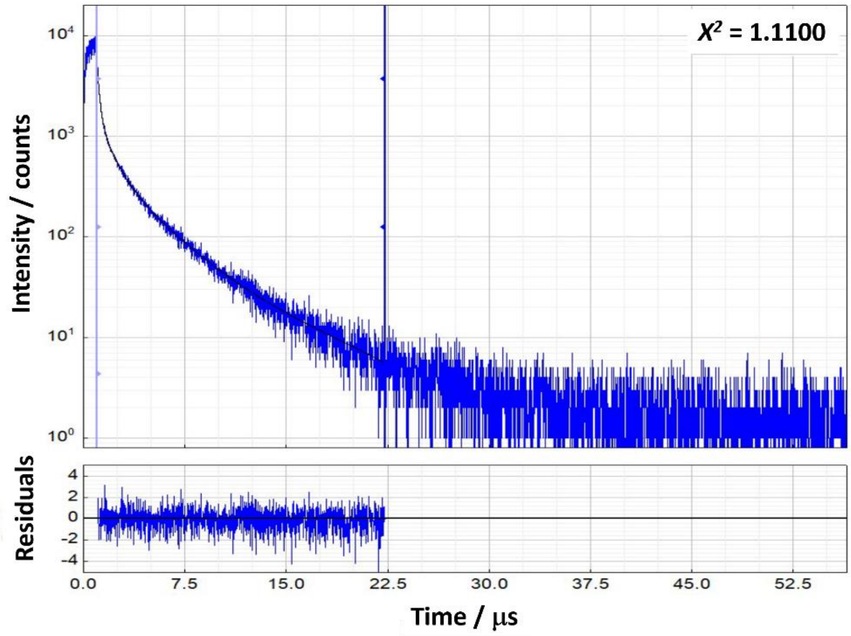

Parameter	Value	Error
A_1[kCnts/Chnl]	0.53	± 0.14
τ_1[ns]	3040	± 1335
I_1[kCnts]	100	± 31
$A_{Rel\,1}$[%]	11.7	± 2.8
$I_{Rel\,1}$[%]	44	± 11
A_2[kCnts/Chnl]	1.059	± 0.085
τ_2[ns]	827	± 287
I_2[kCnts]	55	± 21
$A_{Rel\,2}$[%]	23.5	± 1.4
$I_{Rel\,2}$[%]	24.2	± 8.3
A_3[kCnts/Chnl]	2.88	± 0.24
τ_3[ns]	152	± 18
I_3[kCnts]	27.3	± 5.2
$A_{Rel\,3}$[%]	63.8	± 6.0
$I_{Rel\,3}$[%]	12.1	± 3.9
A_4[kCnts/Chnl]	0.05	± 0.21
τ_4[ns]	14847	± 10644
I_4[kCnts]	43	± 52
$A_{Rel\,4}$[%]	1.0	± 4.6
$I_{Rel\,4}$[%]	19	± 26
$Bkgr_{Dec}$[kCnts]	-0.0060	± 0.0100
$\tau_{Av\,Int}$[ns]	4417	± 1970
$\tau_{Av\,Amp}$[ns]	801	± 87

Fig S41. Left: Time-resolved photoluminescence decay of MNC@OH-PtCx2 in air-equilibrated aqueous solution at 298 K, including the residuals (λ_{ex}= 376.7 nm, λ_{em} = 550 nm). Right: Fitting parameters including pre-exponential factors and confidence limits.

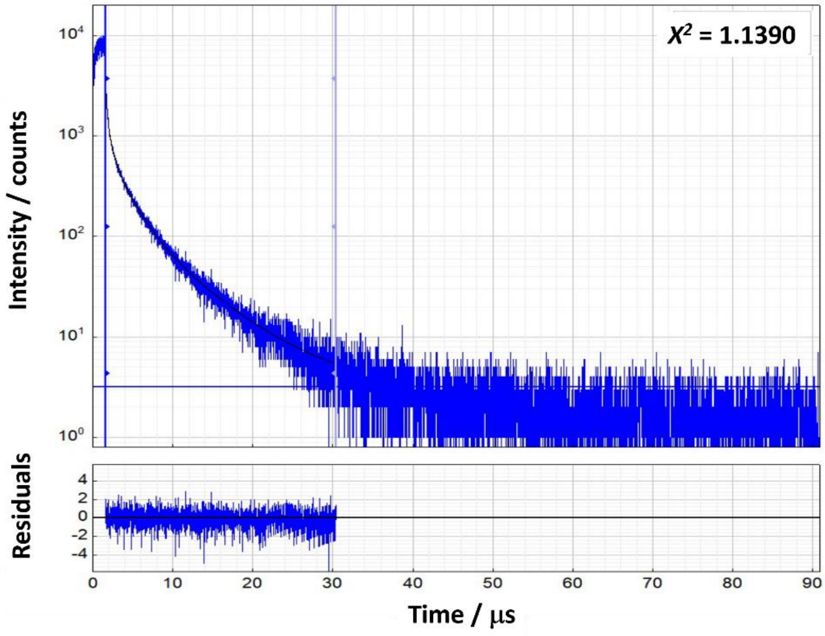

Parameter	Value	Error
A_1[kCnts/Chnl]	0.172	± 0.055
τ_1[ns]	6624	± 975
I_1[kCnts]	71	± 15
$A_{Rel\,1}$[%]	4.8	± 1.6
$I_{Rel\,1}$[%]	35.2	± 7.3
A_2[kCnts/Chnl]	0.797	± 0.082
τ_2[ns]	618	± 103
I_2[kCnts]	30.8	± 8.6
$A_{Rel\,2}$[%]	22.0	± 2.0
$I_{Rel\,2}$[%]	15.2	± 4.2
A_3[kCnts/Chnl]	2.091	± 0.098
τ_3[ns]	165	± 11
I_3[kCnts]	21.5	± 1.9
$A_{Rel\,3}$[%]	57.7	± 2.4
$I_{Rel\,3}$[%]	10.6	± 1.1
A_4[kCnts/Chnl]	0.565	± 0.042
τ_4[ns]	2239	± 435
I_4[kCnts]	79	± 11
$A_{Rel\,4}$[%]	15.6	± 1.1
$I_{Rel\,4}$[%]	39.0	± 5.0
$Bkgr_{Dec}$[kCnts]	0.0032	± 0.0010
$\tau_{Av\,Int}$[ns]	3316	± 171
$\tau_{Av\,Amp}$[ns]	895	± 17

Fig S42. Left: Time-resolved photoluminescence decay of MNC@PMAO-PtCx1 in fluid argon-purged aqueous solution at 298 K, including the residuals (λ_{ex}= 376.7 nm, λ_{em} = 550 nm). Right: Fitting parameters including pre-exponential factors and confidence limits.

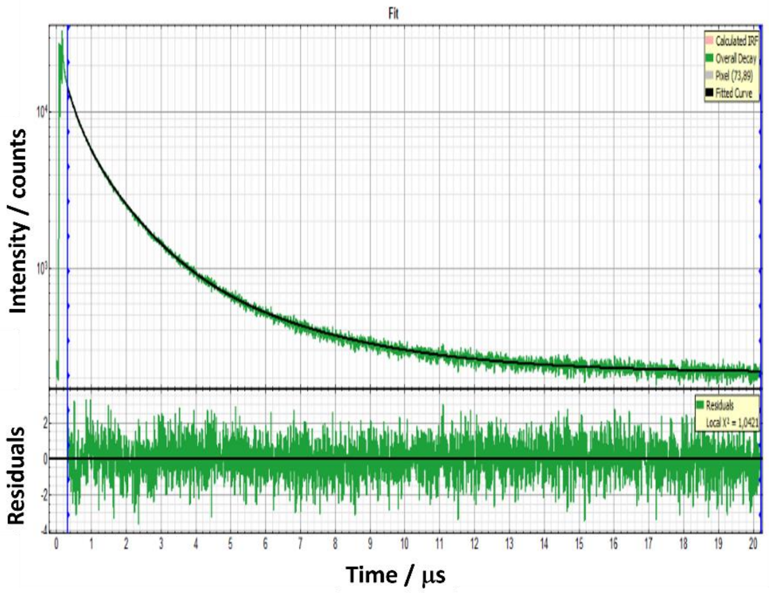

Parameter	Overall Decay
A_1 [kCnts]	1,420 ± 0,055
A_2 [kCnts]	6,930 ± 0,071
A_3 [kCnts]	5,790 ± 0,082
τ_1 [ns]	3416 ± 55
τ_2 [ns]	1073 ± 11
τ_3 [ns]	317,0 ± 6,6
$Bkgr_{Dec}$ [Cnts]	215,00 ± 0,65
I_1 [kCnts]	950 ± 13
I_2 [kCnts]	1452 ± 14
I_3 [kCnts]	359 ± 11
I_{Sum} [kCnts]	2761,0 ± 2,9
A_{Sum} [kCnts]	14,100 ± 0,026
$\tau_{Av Int}$ [ns]	1781 ± 14
$\tau_{Av Amp}$ [ns]	999,0 ± 2,7
$A_{rel 1}$	0,101 ± 0,004
$A_{rel 2}$	0,4898 ± 0,0042
$A_{rel 3}$	0,4095 ± 0,0052

Fig S43. Left: Time-resolved photoluminescence decay **MNC@PMAO-PtCx1** in in 1% agarose phantom at 298 K, including the residuals (λ_{ex}= 355 nm). Right: Fitting parameters**.** obtained by PLIM.

Section 8: References

1. R.K. Harris, E.D. Becker, S.M. Cabral de Menezes, R. Goodfellow, P. Granger, *Pure Appl. Chem.* **2001**, *73*, 1795–1818.

2. P. Mendoza Zélis, D. Muraca, J. S. Gonzalez, G. A. Pasquevich, V. A. Alvarez, K. R. Pirota, F. H. Sánchez, *J. Nanopart. Res.* **2013**, *15*, 1613.

3. G.C. Lavorato, J.C. Azcárate, M.B. Rivas Aiello, J.M. Orozco Henao, P. Mendoza Zélis, M. Ceolin, E. Winkler, M.H. Fonticelli, C. Vericat, *Appl. Surf. Sci.* **2021**, *570*, 151171.

4. M. von Gröning, I. de Feijter, M.C.A. Stuart, I.K. Voetsb, P. Besenius, *J. Mater. Chem. B* **2013**, *1*, 2008–2012.

5. J. Muro-Cruces, A.G. Roca, A. López-Ortega, E. Fantechi, D. del-Pozo-Bueno, S. Estradé, F. Peiró, B. Sepúlveda, F. Pineider, C. Sangregorio, J. Nogues, *ACS Nano* **2019**, *13*, 7716-7728.

6. W.W. Yu, E. Chang, C.M. Sayes, R. Drezek, V.L. Colvin, *Nanotechnology* **2006**, *17*, 4483–4487.

7. E.V. Shtykova, X. Huang, X. Gao, J.C. Dyke, A.L. Schmucker, B. Dragnea, N. Remmes, D.V. Baxter, B. Stein, P.V. Konarev, D.I. Svergun, L.M. Bronstein, *J. Phys. Chem. C* **2008**, *112*, 16809–16817.

8. V. Salgueiriño-Maceira, L.M. Liz-Marzán, M. Farle, *Langmuir* **2004**, *20*, 6946–6950.

9. G.C. Lavorato, R. Das, Y. Xing, J. Robles, F. Litterst, E. Baggio-Saitovitch, M.H. Phan, H. Srikanth, *ACS Appl. Nano Mater.* **2020**, *3*, 1755–1765.
